# External validation and recalibration of the psychosis metabolic risk calculator (PsyMetRiC) in young adults with chronic psychotic disorders in the Netherlands

**DOI:** 10.1192/j.eurpsy.2026.10179

**Published:** 2026-03-09

**Authors:** D.M.C. Quadackers, B.I. Perry, S.S. Gangadin, E. Visser, PHAMOUS investigators, H. Riese, D.C. Cath

**Affiliations:** 1 Department of Psychiatry, UMCG: Universitair Medisch Centrum, Groningen, Netherlands; 2 GGZ Drenthe Mental Health Services, Assen, The Netherlands; 3 University of Birmingham, UK

**Keywords:** cardiometabolic, risk-prediction, chronic, psychosis

## Abstract

**Background:**

People with psychotic disorders have high cardiometabolic risk, yet prediction tools are rarely validated outside early-intervention settings. We externally validated and recalibrated the UK Psychosis Metabolic Risk Calculator (PsyMetRiC) in a Dutch cohort of young adults with psychotic disorders in long-term care.

**Methods:**

We used data from the PHAMOUS registry. Individuals aged 16–35 years, without metabolic syndrome (MetS) at baseline (prevalence 21.2%), were included. MetS incidence over approximately 6 years (last assessment 1–6 years; cumulative incidence 29.1%) was defined using international criteria. Full (biochemical + clinical) and partial (clinical only) PsyMetRiC models were applied to 10 multiply imputed datasets. Discrimination, calibration, and decision-curve analysis (DCA) were assessed before and after logistic recalibration of intercept and slope.

**Results:**

In external validation, *C*-statistics were about 0.69 for the full and 0.67 for the partial model. Both systematically underpredicted MetS risk; recalibration yielded calibration intercepts near 0 and slopes near 1, while discrimination was unchanged. DCA suggested that, across risk thresholds of 0.10–0.35, using recalibrated PsyMetRiC could provide higher net benefit than “treat all” or “treat none.”

**Conclusions:**

In this chronic-care cohort, PsyMetRiC showed moderate discrimination and improved calibration after logistic recalibration. The recalibrated models may support more targeted metabolic monitoring and prevention, but interpretation is limited by registry design, variable follow-up times, reliance on multiple imputation, and modest power for subgroup analyses.

## Introduction

Compared to the general population, patients diagnosed with schizophrenia have more than double the risk of all-cause mortality (standardized mortality ratio 2.5, 95% CI: 2.2–2.4) [1]. These patients not only develop cardiovascular diseases (CVD) nearly twice as often, but are also more likely to die from them [2, 3]. Surviving a cardiovascular event often results in reduced physical activity, significantly diminished quality of life, increased social isolation, and worsening of negative symptoms [4]. Additionally, it results in higher societal costs, including increased healthcare expenditures and productivity losses [5].

In addition, the prevalence of diabetes is 3–10 times higher in this patient group compared to the general population [6, 7]. Worldwide, approximately 13 million individuals with schizophrenia develop diabetes mellitus [8]. In a study by Vinogradova and colleagues, more than one-fifth of this population (mean age 60.9 years) died within 5 years [9]. Patients diagnosed with both schizophrenia and diabetes have a 52% higher mortality risk compared to diabetic individuals without schizophrenia [9].

As a result, cardiometabolic diseases (i.e., CVD and diabetes) contribute significantly to the already substantially reduced life expectancy of patients with schizophrenia, which is shortened by 15–25 years [3, 10, 11]. Multiple factors synergistically explain the increased cardiometabolic morbidity and mortality in this population, including: i) patient- and illness-related factors (e.g., unhealthy lifestyle, cognitive impairments leading to healthcare avoidance), ii) treatment-related factors (e.g., cardiometabolic side effects of psychotropic medications), iii) physician-related factors (e.g., lack of knowledge or non-adherence to clinical guidelines, resulting in insufficient monitoring of physical health), and iv) service-related factors (e.g., unclear responsibility for physical health monitoring among healthcare providers) [12].

To address this health disparity, effective screening methods for cardiometabolic diseases are essential to initiate timely interventions and enable primary prevention. One such method is the assessment of Metabolic Syndrome (MetS), which is considered a precursor to both CVD and diabetes [13, 14]. Various definitions of MetS exist, but the most widely accepted are the criteria from the National Cholesterol Education Program Adult Treatment Panel III (NCEP ATP III) [15]. A diagnosis of MetS is made when at least three of the following parameters are abnormal: elevated fasting glucose, reduced HDL cholesterol, elevated triglycerides, increased waist circumference, and hypertension. If a patient is receiving medication for any of these parameters, the corresponding MetS criterion is considered fulfilled, regardless of current lab values.

Predicting the risk of MetS using validated models allows for earlier intervention, which is preferable to treating the condition after it has already developed. MetS is preferred over other cardiovascular risk assessment methods because: i) many models (e.g., Framingham, SCORE) were developed in the general population and do not adequately account for the elevated cardiovascular risk associated with psychiatric conditions or the use of psychopharmacological medication [16], and ii) even models developed for psychiatric populations (e.g., PRIMROSE) may underpredict risk, particularly in younger patients, due to a heavy weighting of age relative to other risk factors [17–19].

To the best of our knowledge, only the British PsyMetRiC models [20] have been developed specifically to predict the onset of MetS within 6 years in relatively young patients (aged 16–35) with early psychotic disorders. These models have been externally validated in Finland, Spain, Switzerland, Australia, and China [21–24]. However, due to differences in demographics, culture, and healthcare systems across countries, it is essential to verify whether the models maintain acceptable predictive performance and clinical utility before implementation [25].

Both the Netherlands and the United Kingdom are classified as high-income countries with well-developed social market economies [26]. While the Netherlands places relatively greater emphasis on social safety nets and government regulation, the United Kingdom has historically pursued a more liberal economic policy orientation. Supplementary Table 1 provides a detailed comparison of demographic, economic, and healthcare characteristics between the two countries. Notable inter-country differences include: (1) the population density in the Netherlands is approximately 1.5 times higher than in the United Kingdom; (2) linguistic diversity is greater in the Netherlands; (3) the Dutch gross domestic product per 1,000 inhabitants is nearly 1.5 times higher; (4) the Netherlands has a higher number of (psychiatric) hospital beds per capita; and (5) the rate of congestive heart failure hospitalizations per 100,000 population is substantially higher in the Netherlands compared to the United Kingdom (199.4 versus 99.4 in 2013).

Although both the incidence and prevalence of schizophrenia appear to be declining more rapidly in the Netherlands [27], the Dutch age-standardized incidence rate (approximately 22 per 100,000) and prevalence rate (approximately 376 per 100,000) remain higher than those reported in the United Kingdom (approximately 13 and 249 per 100,000, respectively; see Supplementary Table 6 in reference [27]).

The prevalence of metabolic syndrome among Dutch individuals with severe mental illness is estimated to be at least 50% when no age restrictions are applied [28, 29], with comparable rates reported in the United Kingdom [30]. Furthermore, although based on older studies, standardized mortality rates due to cardiovascular diseases among patients with schizophrenia appear to be comparable between the two countries [31, 32].

In addition, it remains unclear whether PsyMetRiC could be flexible and appropriate for use in younger psychosis populations with more chronic illness courses. Our main research question was to verify whether the MetS prediction tools derived in early-intervention services for first-episode psychosis can be transported to, and recalibrated for, a chronic-care setting with longer illness duration and higher baseline metabolic burden. The research objectives of the present study were: (1) to externally validate the full and partial PsyMetRiC models using the Dutch PHAMOUS database; (2) to assess whether simple logistic recalibration (adjusting intercept and slope) can restore calibration; and (3) to examine potential clinical usefulness using decision curve analysis. Our a priori expectations were that discrimination would likely be lower than in the derivation samples, due to reduced heterogeneity and greater comorbidity in chronic care, and that miscalibration-in-the-large would occur, necessitating recalibration.

## Methods

### Data sources

#### PHAMOUS

We used the PHAMOUS database to externally validate the PsyMetRiC prediction models. PHAMOUS was initiated in 2006 by four major mental healthcare institutions in the northern Netherlands (i.e., Lentis Mental Health Institution, GGZ Friesland Mental Health Institution, GGZ Drenthe Mental Health Institution, and the University Centre for Psychiatry of the University Medical Centre Groningen). These institutions deliver a broad spectrum of specialized mental healthcare, with a catchment area of 1.7 million inhabitants in 2015 [33]. Its initial aim was to systematically monitor the physical, mental, and social health of patients with a severe mental illness receiving long-term psychiatric care, as well as to assess the effects of chronic psychotropic medication use [33]. To be included in PHAMOUS, patients aged 18 years and older, only had to fulfil DSM criteria for schizophrenia, schizoaffective disorder, or other psychotic disorders.

Each year, during a PHAMOUS screening, various aspects of patient well-being and treatment are assessed. These include the use of psychotropic medication, quality of life and subjective well-being over the past month, perceived side effects, satisfaction across different life domains, psychosocial functioning, and experiences with mental healthcare services. Additionally, the severity of positive and negative symptoms is evaluated. Physical examinations are also conducted [28], including measurements of weight, height, waist circumference (with a flexible measuring tape between the lower rib and the upper edge of the hip bone), and blood pressure (twice with a manometer, with an interval of 15 seconds), as well as assessments for potential movement disorders. Blood samples are collected to evaluate metabolic parameters, including those required to diagnose MetS. Participants are asked to refrain from caloric intake for 8 hours before blood sample collection, but fasting status is not always reported.

Research on the PHAMOUS dataset has been deemed exempt from the Medical Research Involving Human Subjects Act (WMO) by the Medical Ethical Committee of the University Medical Centre Groningen (METc approval number 2015/347). This exemption was granted because no additional burden is placed on service users, the research involves pre-existing routine healthcare data, and research on the data serves a broad public interest.

As of April 2024, the PHAMOUS database contained data from 2,496 unique patients who met both inclusion criteria: a diagnosis of a psychosis-spectrum disorder and an age between 16 and 35 years at baseline. All of the analyses were conducted using R version 4.4.2.

#### Inclusion and exclusion criteria

Inclusion criteria for the present study were as follows [20]: individuals aged between 16 and 35 years at baseline (i.e., the latest PHAMOUS metabolic screening at which the individual had the least missing data on PsyMetRiC predictors and did *not* meet criteria for MetS); a minimum follow-up duration of one year, and a maximum of 6 years; and a diagnosis of a psychotic spectrum disorder according to ICD-10 codes F06.0–2, F20–F31, F32.3, F33.3, or F53.1. The exclusion criterion was presence of missing data for all predictor variables or for the outcome variable. Patients with pre-diabetes or diabetes were not excluded, because these conditions are captured in the MetS outcome definition. Supplementary Figure 1 presents a flowchart illustrating the selection process of the study participants. One notable difference between the present study and the original PsyMetRiC study is the latter included individuals newly enrolled into psychosis early intervention services (i.e., first episode psychosis). In this study, we included all individuals with psychosis-spectrum disorders, in order to for the first time appraise model performance in patients with more chronic illness trajectories.

#### Outcome

Following the approach used in the original PsyMetRiC study [20], we applied the harmonized International Diabetes Federation/AHA-NHLBI criteria for defining metabolic syndrome as a binary outcome [15]. This definition includes either an ethnicity-specific waist circumference (≥94 cm for Caucasian men and ≥ 80 cm for Caucasian women; ≥90 cm for men and ≥ 80 cm for women of other ethnic backgrounds) or a body mass index (BMI) exceeding 29.9 kg/m^2^, in combination with at least two of the following: triglyceride levels ≥1.70 mmol/L; HDL cholesterol <1.03 mmol/L for men or < 1.29 mmol/L for women; systolic blood pressure > 130 mmHg; or fasting plasma glucose >5.60 mmol/L. For participants with multiple follow-up assessments, we selected the most recent follow-up occurring between 1 and 6 years after baseline, prioritizing the observation with the fewest missing data points.

#### The PsyMetRiC algorithms

Two multivariable prediction algorithms were developed in the original paper to estimate the 6-year risk of incident metabolic syndrome in young individuals aged 16 to 35 years with psychosis or at risk of developing psychosis.

To construct the PsyMetRiC models, the authors [20] employed a forced-entry penalized logistic regression approach, instead of time-to-event models. The simultaneous inclusion of all pre-specified predictors were selected based on clinical relevance, prior research, and input from a young persons’ advisory group. The prespecified predictor set of the original PsyMetRiC development study did not include fasting glucose or HbA1c, because the combination of HDL-cholesterol and triglycerides is a more sensitive marker of early insulin resistance compared to glycaemic indices [34].

Two versions of the PsyMetRiC algorithm were developed: the “full model,” and the “partial model.” The full model includes both clinical and biochemical predictors: age, sex, ethnicity, body mass index (BMI), smoking status, use of metabolically active antipsychotic medication, HDL cholesterol concentration, and triglyceride concentration. The partial model excludes the biochemical variables (HDL and triglycerides), allowing for use in settings where blood test results are unavailable. Both models use the harmonized international definition of metabolic syndrome as the predicted binary outcome. Antipsychotic medication (including olanzapine, quetiapine, risperidone, and clozapine) was considered metabolically active based on previous research [20, 35, 36]. We refer to Supplementary Table 4 of the original development study for more information about this classification process [20]. In the development study, only antipsychotic treatment was included as a medication predictor; other concomitant drugs were not part of the predictor set. In addition, the definition of metabolic syndrome used in our study already incorporates the effect of somatic medications. Consistent with international criteria, treatment for elevated blood pressure or dyslipidaemia counts as meeting the corresponding metabolic syndrome component. For example, the use of antihypertensive medication is sufficient to fulfil the hypertension criterion, and lipid lowering medication contributes to the dyslipidaemia criteria.

### Statistical analysis

#### Sample preparation and estimation of analytic precision

All predictor and outcome variables in the PHAMOUS and original PsyMetRiC datasets were expressed in identical units, eliminating the need for unit conversion. The PHAMOUS ethnicity groups were originally coded into seven categories (i.e., Caucasian/White, Negroid/Black, Asian, Indian/Latin-American, Turkish, Maroccan, and other). If a patient belonged to one of the last four categories, their ethnicity was recoded to the “Asian/other” group. To address missing data, we performed multiple imputation using the MICE package in R [37], yielding 10 completed datasets using 5 iterations. We used MICEs “quickpred” function, with a mincor of 0.06 and a minpuc of 0.05, to select suitable predictors from baseline, outcome, and auxiliary variables. Rubin’s rules [38] were then applied to pool estimates across imputations for subsequent analyses. The resulting model coefficients already reflect optimal use of incomplete data; when the models are subsequently applied, the most reliable risk estimate is obtained when all predictors for the new individual are observed [39]. To evaluate the consistency of graphical representations between PHAMOUS and the development sample – such as frequency distributions, calibration plots, and decision curve analysis plots – we conducted visual inspections across all imputed datasets. One dataset was randomly selected for illustrative purposes. The full set of graphical outputs is provided in the Supplementary Materials. To assess baseline comparability between the original and external validation sample, we conducted independent samples *t*-tests for continuous variables and Chi-square tests for categorical variables.

#### Primary external validation analysis and exploratory subgroup analysis

To evaluate the distribution of predicted probabilities for the outcome, histograms were utilized. The model’s performance was primarily evaluated in terms of discrimination and calibration. Discriminative ability was quantified using the C-statistic. Calibration was assessed through graphical calibration curves. Additional performance metrics included the Nagelkerke R^2^ index, the calibration intercept, the calibration slope, and the Brier score.

We also perform an exploratory subgroup analysis stratified by sex. Only this demographic subgroup analysis is performed, due to limited sample sizes in the other subgroups (such as ethnicity).

#### Recalibration and generation of PsyMetRiC-NL models

To adjust for baseline differences between the Dutch (NL) and UK samples, we recalibrated the original logistic regression equations of PsyMetRiC to this Dutch, more chronic setting, using logistic calibration. This method involves modifying both the intercept and the overall calibration slope of the linear predictor, resulting in the development of a ‘full’ and a ‘partial’ PsyMetRiC-NL model [40].

#### Clinical usefulness

Decision curve analysis (DCA) is a method used to evaluate the clinical utility of prediction models by quantifying their net benefit relative to default strategies, such as treating all patients or none. Net benefit is assessed across a continuum of threshold probabilities, which represent the minimum predicted risk at which clinical intervention is considered appropriate. At each threshold, individuals are classified as test-positive if their predicted probability equals or exceeds that value. Intervention approaches should be based on economic evaluation, with stakeholder input on acceptability, and flexibility to align with local care pathways. Unlike traditional performance metrics such as discrimination or calibration, DCA explicitly accounts for the clinical consequences of decisions based on model predictions [41]. In our analysis, we used the same upper limit for the threshold probability of developing metabolic syndrome as the original paper, namely 0.35.

## Results

### Sample

The Dutch PHAMOUS sample, being more chronic in illness course, exhibited significant differences compared to the UK development sample: patients were significantly older: in the full cohort with available data (*n* = 2104), the mean age of onset of psychotic symptoms was 21.9 years (SD 5.3; median 21; IQR 8), illness duration at the first PHAMOUS assessment had a median of 4 years (IQR 8), they were predominantly White, more likely to smoke, used less metabolically active antipsychotics (at the first PHAMOUS screening, the distribution of antipsychotic use was as follows: clozapine 16.5% (*n* = 412), olanzapine 25.6% (*n* = 640), risperidone 13.6% (*n* = 340), quetiapine 8.9% (*n* = 223), aripiprazole 13.3% (*n* = 332), and haloperidol 3.8% (*n* = 95)), and presented with less favourable metabolic syndrome parameters, including lower HDL cholesterol, higher BMI, and elevated systolic blood pressure. The distribution of the follow up showed prior to the application of inclusion criteria a median of 3.5 years, and an IQR of 7.3 years. Additionally, the prevalence of MetS was higher both at baseline (21.2%) and at follow-up (29.1%) (see Table 1). A total of *n* = 1,364 patients met all inclusion criteria (see Supplementary Figure 1, which also includes a summary of the diagnostic mix at first PHAMOUS assessment).

**Table 1.** Characteristics of the original PsyMetRiC sample, and external validation PHAMOUS sample

	Original PsyMetRiC development sample (UK)	External validation sample PHAMOUS (Netherlands)	Between-group differences
Included sample size, *N*	651	1364	–
Age in years, mean (SD)	24.52 (4.91)	27.5 (4.74)	*t* = 13.04; *p* < 0.0001
White European/NR Ethnicity, *N* (%)	360 (55.3)	1155 (84.7)	**χ**^2^ = 204.149; *p* < 0.0001
Black/African-Caribbean ethnicity, *N* (%)	109 (16.74)	90 (6.6)	**χ**^2^ = 50.881; *p* < 0.0001
Asian/other ethnicity, *N* (%)	181 (27.80)	119 (8.7)	**χ**^2^ = 126.929; *p* < 0.0001
Male sex, *N* (%)	440 (67.59)	983 (72.1)	**χ**^2^ = 4.320; *p* = 0.0377
HDL at baseline, mmol/L, mean (SD)	1.88 (0.57)	1.29 (0.357)	*t* = 28.32; *p* < 0.0001
Triglycerides at baseline, mmol/L, mean (SD)	1.39 (1.06)	1.32 (0.792)	*t* = 1.66; *p* = 0.0979
BMI at baseline, kg/m^2^, mean (SD)	23.63 (5.43)	24.9 (4.39)	*t* = 5.61; *p* < 0.0001
FPG at baseline, mmol/L, mean (SD)	5.19 (1.28)	5.11 (0.694)	*t* = 1.82; *p* = 0.0695
Systolic BP at baseline, mmHg, mean (SD)	120.65 (11.68)	122 (13.6)	*t* = 2.18; *p* = 0.0295
Prescribed a more-metabolically-active antipsychotic, *N* (%)	455 (69.89)	792 (58.1)	**χ**^2^ = 25.963; *p* < 0.0001
Smoking at baseline, *N* (%)	315 (48.39)	955 (70.0)	**χ**^2^ = 88.259; *p* < 0.0001
Follow-up time, years, mean (SD)	1.86 (1.32)	3.43 (1.55)	*t* = 22.27; *p* < 0.0001
Metabolic syndrome at baseline, *N* (%)	49 (6.58)	368 (21.2)	**χ**^2^ = 68.411; *p* < 0.0001
Metabolic syndrome at follow-up, N (%)	109 (16.74)	397 (29.1)	**χ**^2^ = 35.786; *p* < 0.0001

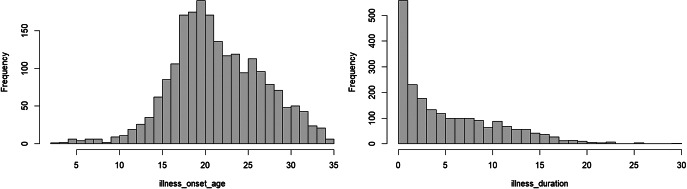
 *Note*: Mean age of onset of psychotic symptoms: 21.9 years (SD 5.3; median 21; IQR 8), illness duration at first PHAMOUS assessment had a median of 4 years (IQR 8).

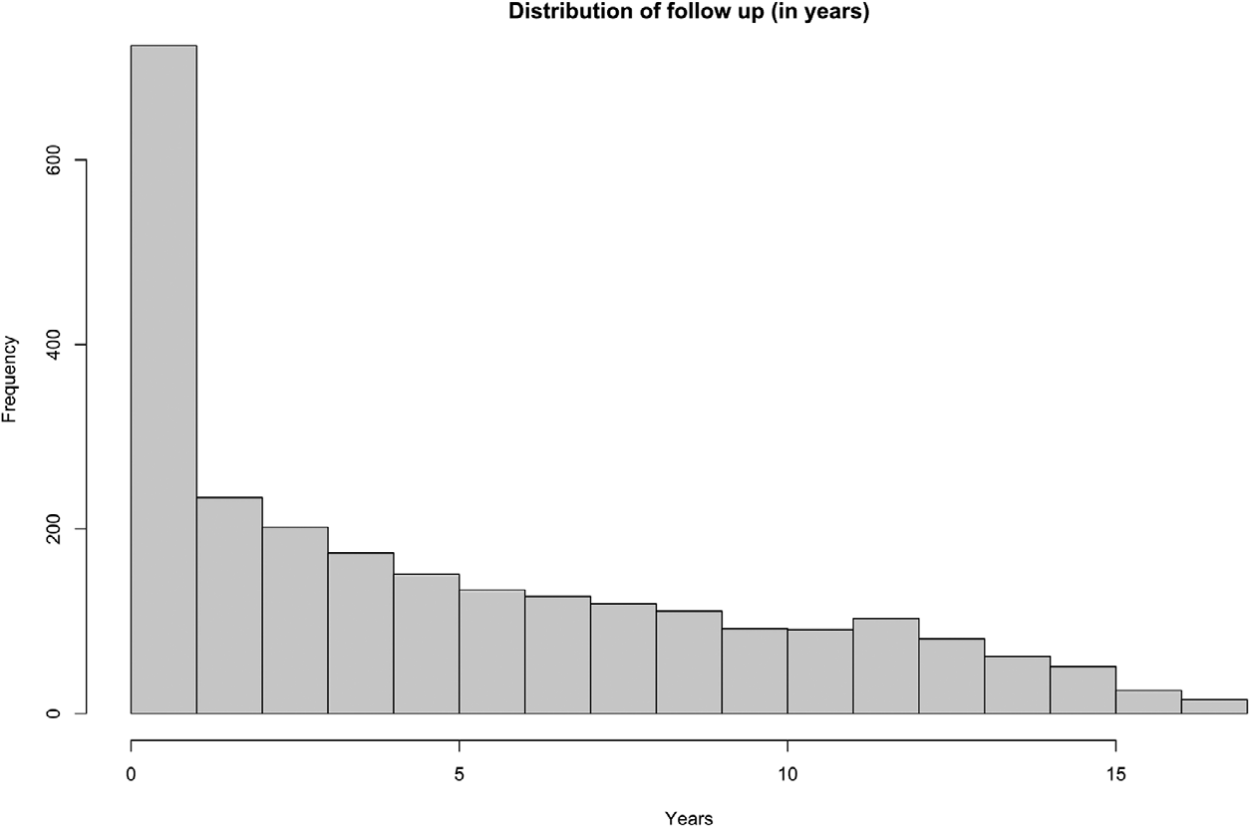
 *Note*: Distribution of follow-up (before applying inclusion criteria), with a median of 3.5 years, and an IQR of 7.3 years.

#### Primary external validation analysis

**Figure 1.**
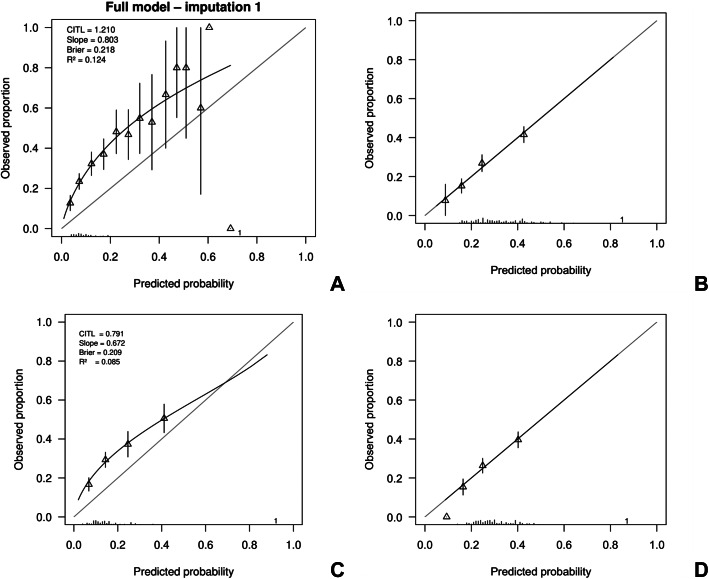
Calibration plots of PsyMetRiC in PHAMOUS sample before and after logistic calibration. *Note*: Panel A represents the initial analysis using the full model; Panel B shows the full model after applying logistic calibration. Panel C corresponds to the initial analysis with the partial model, while Panel D displays the calibrated version of the partial model. The calibration plots compare predicted probabilities (x-axis) with actual outcomes (y-axis). A perfect match between prediction and reality would follow the red diagonal line. The black line represents the model’s actual calibration performance. Triangles indicate grouped participant data at each decile of predicted risk, with vertical black lines showing 95% confidence intervals. Logistic calibration adjusts for potential differences in baseline risk between populations by recalculating the model’s intercept. It also updates the slope, assuming that while the relative influence of predictors remains consistent, their absolute impact may vary.

The highest frequency of predicted probabilities using the UK full model occurred between 0.05 and 0.10 (before calibration). For the partial model, the highest frequency of predicted probabilities occurred between 0.10 and 0.20 before calibration (Supplementary Figure 2). Both samples showed similarly right-skewed distributions in their plots, indicating a concentration of lower predicted probabilities with a long tail toward higher values. Table 2 presents the predictive performance of both the full and partial PsyMetRiC models – before and after logistic recalibration (PsyMetRiC-NL) – as evaluated within the PHAMOUS cohort. Application of the original UK models generally resulted in underprediction within the PHAMOUS population (Figure 1; Supplementary Figure 3). Only for the highest predicted probabilities, the partial models tended to overpredict the observed risk.

**Table 2.** Assessment of the predictive accuracy of the full and partial PsyMetRiC models, both prior to and following logistic calibration (PsyMetRiC-NL), within the PHAMOUS cohort

Measure of predictive performance in PHAMOUS	Primary analysis (estimated 95% CI)		After logistic calibration (estimated 95% CI)	
	Full model	Partial model	PsyMetRiC-NL full model	PsyMetRiC-NL partial model
*C*-statistic	0.6932 (0.6797–0.7064)	0.6666 (0.6472–0.6854)	0.6932 (0.6797–0.7064)	0.6666 (0.6472–0.6854)
R^2^	0.1338 (0.1170–0.1507)	0.1008 (0.0780–0.1236)	0.1338 (0.1224–0.1453)	0.1008 (0.0780–0.1236)
Calibration intercept	1.2154 (1.1608–1.2700)	0.79241 (0.7416–0.8432)	0 (0–0)*	0 (0–0)*
Calibration slope	0.8377 (0.7826–0.8929)	0.7436 (0.6456–0.8417)	1 (1–1)*	1 (1–1)*
Brier score	0.2177 (0.2115–0.2239)	0.2070 (0.2019–0.2121)	0.1864 (0.1830–0.1898)	0.1913 (0.1877–0.1949)

*Note*: To evaluate how well the model distinguishes between individuals with and without the outcome, we used the *C*-statistic. This metric reflects the likelihood that a randomly chosen individual with the condition will receive a higher predicted risk score than someone without it. Model calibration – how closely predicted risks align with actual outcomes – is assessed using the calibration intercept and the calibration slope. The Brier score, which combines aspects of both discrimination and calibration, offers a summary measure of prediction accuracy; lower values are better. For more comprehensive information about the predictive performance of the original models, we refer to [20]. *By design, as the same data is used to derive *and* evaluate logistic recalibration.

#### Exploratory subgroup analysis

In exploratory sex-stratified analyses, we evaluated the performance of both PsyMetRiC models separately in men and women. For the full model, the pooled *C*-statistic was 0.68 (95% CI 0.64–0.72) in men and 0.72 (95% CI 0.66–0.78) in women. For the partial model, the corresponding C-statistics were 0.64 (95% CI 0.60–0.69) and 0.71 (95% CI 0.64–0.78), respectively. Calibration-in-the-large was positive in both sexes, with CITL estimates of 1.23 (SE 0.08) in men and 1.17 (SE 0.13) in women for the full model, and 0.84 (SE 0.08) and 0.64 (SE 0.13) for the partial model. Calibration slopes were below 1 in both sexes but of similar magnitude (full model: 0.80 [SE 0.10] in men vs 0.91 [SE 0.16] in women; partial model: 0.69 [SE 0.11] vs 0.83 [SE 0.16]). Brier scores were slightly lower in women than in men (e.g., 0.19 vs 0.23 for the full model). Overall, these analyses suggest comparable discrimination and broadly similar calibration patterns in males and females, although uncertainty around female-specific estimates was larger. Detailed results are provided in Supplementary Table 2, and sex-specific calibration plots for all imputations are shown in Supplementary Figure 4.

### Algorithm recalibration and generation of PsyMetRiC-NL versions

After applying the new linear predictor intercept and coefficients (as specified in Supplementary Figure 2), we obtained the recalibrated full model, called the PsyMetRiC-NL full model. Similarly, the recalibrated partial model, referred to as the PsyMetRiC-NL partial model, was derived by applying new linear predictor intercept and coefficients. The probability that a Dutch PHAMOUS patient will develop MetS within 6 years can be calculated using the formulas given below.

• LP_original PsyMetRiC UK full model_ = −6.439813 + 0.006233226 ∙ Age + 0.004258861 ∙ Black + 0.211217746 ∙ Asian/other + 0.222300765 ∙ Male + 0.141186241 ∙ BMI + 0.153691193 ∙ Smoker + 0.497552758 ∙ Metab. active AP − 0.399013329 ∙ HDL + 0.343528440 ∙ Triglycerides




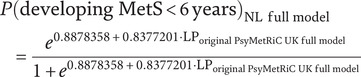



• LP_original PsyMetRiC UK partial model_ = −6.973829 + 0.00633115 ∙ Age + 0.07548129 ∙ Black + 0.29285950 ∙ Asian/other + 0.31460036 ∙ Male + 0.16912161 ∙ BMI + 0.24751854 ∙ Smoker + 0.60013558 ∙ Metab. active AP




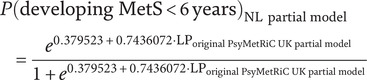



After calibration, the highest frequency of predicted probabilities for the full model occurred between 0.15 and 0.20. For the partial model, the highest frequency of predicted probabilities occurred around 0.20 (Supplementary Figure 2). The recalibrated predicted probabilities closely aligned with the observed outcome proportions (Figure 1; Supplementary Figure 3). By design, both recalibrated models exhibited calibration intercepts around 0, and calibration slopes around 1.

#### Clinical usefulness

**Figure 2.**
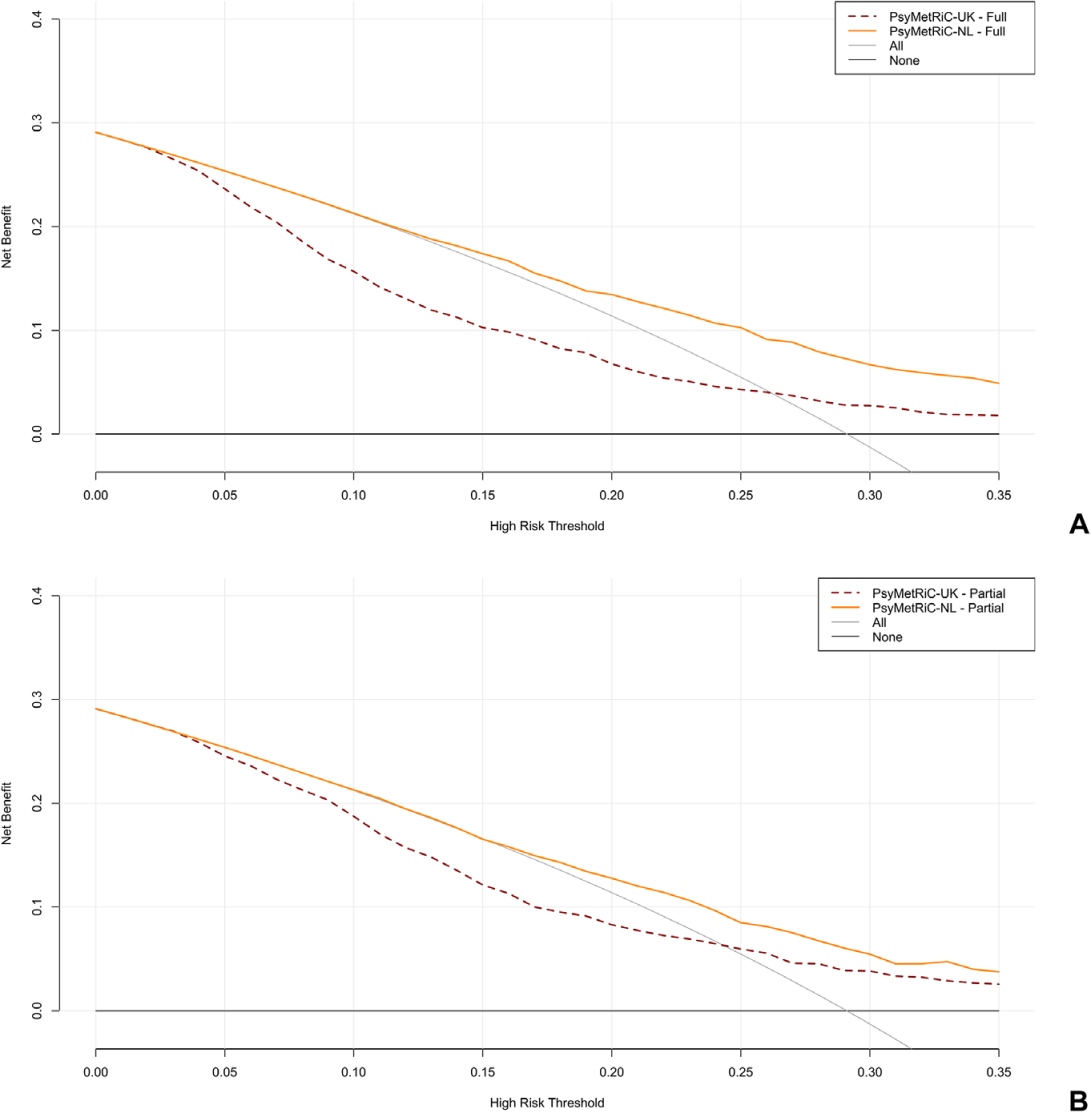
Clinical usefulness of PsyMetRiC in PHAMOUS sample before (PsyMetRiC-UK) and after (PsyMetRiC-NL) logistic calibration. *Note*: Panel A shows the full model applied to the PHAMOUS cohort, while Panel B presents the partial model results. The graph illustrates the net benefit (*y*-axis) of both the full and partial PsyMetRiC models across various risk thresholds (*x*-axis). The red dotted line represents the original model applied directly to the data, while the orange solid line shows the recalibrated version tailored to the Netherlands. For comparison, the grey solid line reflects the strategy of treating all individuals, and the black solid line represents treating none. Decision curve analysis (DCA) focuses on clinically relevant risk thresholds. In this case, the upper limit of 0.35 corresponds to a roughly 1-in-3 chance of developing metabolic syndrome without intervention – thresholds beyond this are unlikely to be acceptable in practice. A curve falling below the zero line (y < 0) indicates net harm, meaning more individuals are unnecessarily treated than correctly identified.

Figure 2 shows that applying a risk threshold of at least 0.17 to the PsyMetRiC NL models outperformed default strategies of treating all or none, yielding higher net benefit. The PsyMetRiC-UK full and partial models outperformed default strategies at higher risk thresholds (i.e., risk thresholds ≥0.25), but had lower clinical utility compared to the recalibrated NL-models. For a risk threshold of 0.25, the pooled net benefit of PsyMetRiC-NL full was 0.106 (95% CI: 0.081–0.132), which is equivalent to 106 net true positives per 1000 patients, under the threshold-specific weighting of false positives. This value indicates that the model is superior to both the ‘treat-all’ and ‘treat-none’ strategies. DCA-plots for the other nine imputed datasets are displayed in Supplementary Figure 5.

## Discussion

This study presents the first external validation and recalibration of the Psychosis Metabolic Risk Calculator (PsyMetRiC) in a Dutch cohort of young individuals with psychotic disorders. Our findings align with recent syntheses demonstrating that metabolic syndrome is highly prevalent and shows both shared and disorder-specific features in psychotic disorders [42, 43]. It is the first study to appraise the performance of PsyMetRiC in predicting the incidence of MetS in a population of young people with psychosis-spectrum disorders with more chronic illness trajectories. Our findings are consistent with previous international validations conducted in Finland [21], Spain and Switzerland [22], Australia [23], and Hong Kong [24], and contribute to the generalizability process of PsyMetRiC across diverse healthcare systems and populations.

### Model performance and calibration

The c-statistic values of the PsyMetRiC models were modest, around 0.69, which indicates substantial overlap in predicted risk between individuals who do and do not develop MetS. The reduced discriminative ability compared to the original UK development sample may be attributed to several factors.

First, the Dutch PHAMOUS cohort differed demographically and clinically from the UK development sample. These differences likely influenced the distribution of risk factors and may have attenuated the model’s ability to distinguish between individuals who did and did not develop MetS [19]. Patients in the PHAMOUS cohort were, on average, older, more likely to smoke, had higher baseline BMI and systolic blood pressure levels, and lower HDL cholesterol levels. In addition, men were overrepresented in our validation sample, which resulted in substantially fewer MetS events among women. Sex-stratified external validation suggested that discrimination and overall calibration patterns of PsyMetRiC were broadly comparable in males and females: for example, in PHAMOUS, the pooled C-statistics were 0.68 in men and 0.72 in women for the full model, and 0.64 and 0.71, respectively for the partial model, with calibration slopes of 0.80 vs 0.91 (full) and 0.69 vs 0.83 (partial). PsyMetRiC should therefore be applied in women with awareness that sex-specific performance is less precisely estimated, and further validation in larger, more gender-balanced cohorts is needed to confirm – and, if necessary, refine – its performance in women.

Second, the higher prevalence of MetS at follow-up in the Dutch cohort (29.1%) compared to the UK sample (16.7%) may have affected model calibration. In addition, the PHAMOUS cohort may show less variability in key predictors or measurement error in registry variables, which could attenuate associations. Calibration analysis revealed systematic underprediction of risk by the original UK models, particularly at lower predicted probabilities. This pattern is consistent with prior external validations of PsyMetRiC, where miscalibration was frequently observed and attributed to population-specific differences in baseline risk and predictor distributions [20].

To address this, we applied logistic recalibration to adjust the intercept and slope of the original models, resulting in the Dutch-specific PsyMetRiC-NL versions. Following recalibration, both models demonstrated by design excellent calibration, with calibration intercepts near zero and slopes close to one. These findings underscore the importance of recalibrating prediction models before clinical implementation in new populations, particularly when baseline risk and predictor distributions differ from the development sample [44].

### Clinical utility

Beyond statistical performance, the clinical utility of a prediction model is a critical consideration for implementation [45]. Decision curve analysis (DCA) provides a framework for evaluating the net benefit of using a model across a range of risk thresholds, accounting for the trade-off between true and false positives. In our study, both the original and recalibrated PsyMetRiC models demonstrated improved net benefit compared to default strategies of treating all or no patients. This finding is particularly relevant in psychiatric populations, where overtreatment and undertreatment both carry significant risks. For example, unnecessary pharmacological interventions may exacerbate side effects or reduce adherence [46], while missed opportunities for early intervention may lead to irreversible cardiometabolic damage [47–49]. With regard to thresholds, at present, there are no empirically established risk cut-offs for metabolic syndrome that define when preventive pharmacological treatment (e.g., co-initiating metformin) should be started in first-episode psychosis. Any such threshold would need to be developed and validated in prospective studies, and carefully balanced against potential adverse effects and patient preferences. We therefore see PsyMetRiC primarily as a decision-support tool that can inform shared decision-making rather than dictate a single risk threshold for pharmacological prevention.

### Limitations

Our findings should be interpreted in light of several limitations. First, although we applied the original inclusion and exclusion criteria, the PHAMOUS cohort is a naturalistic registry of young people in long-term psychosis care from a single Dutch region, rather than an early-intervention sample; selection into long-term services, the regional scope of the registry, and the exclusion of individuals with MetS at baseline may limit generalizability to other treatment settings, healthcare systems, and illness stages. Second, follow-up assessments occurred at varying intervals between 1 and 6 years, and we defined the outcome using the last available measurement within this window; we therefore did not model time-to-event, competing risks, or informative censoring, and our validation pertains to an approximate 6-year risk under these pragmatic assumptions rather than to a strictly fixed prediction horizon. Third, several predictors and MetS components had missing values, which we addressed using multiple imputation under a missing-at-random assumption; however, it is plausible that more metabolically unwell patients were less likely to have complete laboratory data, so some residual bias due to missing-not-at-random mechanisms cannot be excluded. Fourth, although PHAMOUS uses standardized protocols, we could not fully characterize potential changes in assay methods, fasting status, or measurement conditions over time, which may have introduced additional measurement error and attenuated model performance. Fifth, our recalibration strategy deliberately restricted itself to simple logistic calibration (updating a single intercept and slope), and we did not re-estimate individual predictor coefficients or undertake more extensive model revision, so any differences in the relative effects of predictors between the UK and Dutch settings may remain partly unaccounted for. Sixth, despite the large overall sample, some clinically important subgroups were relatively small: even after sex-stratified analyses, performance estimates for women were imprecise, and we were underpowered to provide stable, fully stratified estimates by ethnicity, diagnosis, or follow-up duration; PsyMetRiC-NL should therefore be used in these subgroups with appropriate caution and confirmed in future work. Finally, our decision-curve analyses necessarily rely on assumptions about plausible risk thresholds and the nature and effectiveness of the preventive interventions triggered by the model; furthermore, at the moment, there are no established MetS probability thresholds that guide preventive action. The projected clinical utility should thus be regarded as illustrative rather than as proof of benefit, and prospective implementation studies will be needed to determine real-world impact.

### Implications for clinical practice

Before any risk prediction model, including PsyMetRiC, can be routinely implemented in clinical practice, it must obtain regulatory approval, undergo health economic evaluation, and be assessed by relevant stakeholders to ensure adaptable integration within heterogeneous local care pathways. Given the high prevalence of MetS in this population, as documented in recent psychosis-specific reviews and meta-analyses [42, 43], and the well-documented underdiagnosis and undertreatment of cardiometabolic risk factors [28], the implementation of a validated, easy-to-use prediction tool could facilitate earlier identification and targeted intervention.

PsyMetRiC provides individualized risk estimates, which may support shared decision-making between clinicians and patients. Personalized risk communication has been shown to improve patient engagement and adherence to preventive strategies [50]. These personalized predictions can facilitate clinicians to initiate primary prevention strategies for MetS, rather than waiting until the condition manifests clinically. In young individuals with psychosis, this is particularly relevant: timely lifestyle interventions – such as dietary changes or increased physical activity in cases of overweight – can help prevent substantial burden later in life. Additionally, PsyMetRiC may support clinicians in making informed decisions about switching to antipsychotic medications with a lower metabolic risk profile, thereby reducing the likelihood of iatrogenic metabolic deterioration. However, it is important to note that no trial to date has demonstrated that using PsyMetRiC (or any similar psychosis-specific MetS risk tool) improves clinical outcomes, so at present the model should be viewed as a decision-support aid rather than a stand-alone basis for treatment escalation or de-escalation.

However, successful implementation will require more than technical validation. Integration into electronic health records, clinician training, regulatory approval, and the development of clear clinical pathways for high-risk individuals are essential next steps [51]. Additionally, stakeholder engagement – including input from patients, clinicians, and policymakers – will be critical to ensure that the tool is acceptable, feasible, and aligned with clinical priorities [52].

### Future research directions

While recalibration improved model performance in our sample, future efforts should focus on dynamic model updating using larger, more diverse datasets. This may include incorporating additional predictors such as family history of cardiometabolic disease, physical activity levels, dietary habits, and socioeconomic status [19]. These variables may capture important dimensions of risk not currently included in the model and could enhance predictive accuracy. Centre-specific multiple-imputation strategies to enable safe use of PsyMetRiC when some predictors are systematically missing could also be considered. With regard to modelling strategy, more complex non-linear approaches such as machine-learning algorithms should only be pursued if they offer clear, externally validated gains in discrimination and calibration over simpler models. Many machine-learning based prediction tools fail to outperform conventional regression models on external validation and often show poorer calibration; any additional complexity would therefore need to be justified by demonstrable improvements in performance while preserving sufficient interpretability for clinical use. PsyMetRiC’s current logistic regression framework offers transparency and ease of implementation, which are important advantages in clinical settings.

Another important avenue for future research is the evaluation of PsyMetRiC’s impact on clinical outcomes. While predictive performance is a necessary first step, the ultimate goal is to improve patient care [53]. Prospective impact studies -such as cluster-randomized or stepped-wedge implementation trials comparing usual care with PsyMetRiC-guided care- are needed to determine whether use of the tool leads to earlier detection of MetS, improved management of cardiometabolic risk factors, and ultimately reductions in morbidity and mortality.

Given the transdiagnostic nature of MetS [54], further validation is warranted in other Dutch psychiatric populations, including individuals with severe anxiety and/or mood disorders, comorbid substance use disorders, or those receiving care in forensic or inpatient settings. Extending PsyMetRiC to older adults (e.g., within 5 years of psychosis onset, irrespective of age) could also be valuable. Such an extension would require model redevelopment or formal model updating (e.g. re-estimation of age effects and recalibration) in datasets that include sufficient numbers of older patients, which was beyond the scope of the present study. In addition, hormonal contraceptive use, pregnancy/postpartum status, and menopausal stage are clinically important determinants of metabolic risk in women and could be considered in future development or updating of cardiometabolic risk tools for people with psychosis. However, the addition of predictors has to be balanced against, e.g., the risk of overfitting. In line with good practice for external validation, we did not introduce new predictors that were absent from the original model. Future studies will hopefully help to firmly establish the generalizability/transportability of PsyMetRiC-NL and inform potential adaptations for specific subgroups [55].

## Conclusion

This study provides evidence that, after logistic recalibration, the PsyMetRiC full and partial models achieve moderate discrimination and substantially improved calibration when predicting 6-year risk of metabolic syndrome in a Dutch chronic-care cohort of young adults with psychotic disorders. In this context, PsyMetRiC-NL may help clinicians to prioritize and tailor metabolic monitoring and preventive interventions, rather than relying solely on routine, non-stratified screening.

However, our findings should be viewed as an important step towards clinical implementation. Further external validations in other healthcare systems and service configurations, more detailed evaluation in key subgroups, and prospective impact studies (e.g., implementation or cluster-randomized trials) *after* regulatory certification, economic evaluation, and stakeholder input, are needed to determine whether using PsyMetRiC in routine practice improves detection and management of cardiometabolic risk and is acceptable to patients and clinicians. Only after such work will it be appropriate to consider widespread adoption of PsyMetRiC-based risk stratification in psychosis services.

## Supporting information

10.1192/j.eurpsy.2026.10179.sm001 Quadackers et al. supplementary material Quadackers et al. supplementary material

## Data Availability

In accordance with the General Data Protection Regulation (GDPR), the dataset is classified as pseudonymized rather than fully anonymized, and thus still qualifies as personal data. Since participants did not provide explicit consent for public sharing of their information, legal and ethical standards prevent us from making the dataset openly accessible. Researchers may request access via the Rob Giel Research Center (Data Science Center), subject to privacy safeguards and ethical review.

## Supplementary material

The supplementary material for this article can be found at http://doi.org/10.1192/j.eurpsy.2026.10179.

## PHAMOUS investigators

Frederike Jörg (Department of Psychiatry, Interdisciplinary Center Psychopathology and Emotion Regulation, University of Groningen, University Medical Center Groningen, Groningen, the Netherlands; Department of Education and Research, Friesland Mental Health Care Services, Leeuwarden, the Netherlands), Stynke Castelein (Lentis Research, Lentis Psychiatric Institute, Groningen, the Netherlands; Clinical Psychology and Experimental Psychopathology, Faculty of Behavioural and Social Sciences, University of Groningen, Groningen, the Netherlands), Edith Liemburg (GGZ Friesland, Department of Research, Leeuwarden, the Netherlands; Department of Psychiatry, Rob Giel Research Center, University of Groningen, University Medical Center Groningen, Groningen, the Netherlands), and Lisette van der Meer (Department of Rehabilitation, Lentis Psychiatric Institute, Zuidlaren, the Netherlands; Department of Clinical and Developmental Neuropsychology, University of Groningen, Groningen, the Netherlands).
