# Supplementary material for: External validation and recalibration of the psychosis metabolic risk calculator (PsyMetRiC) in young adults with chronic psychotic disorders in the Netherlands
Source: Eur Psychiatry. 2026 Mar 9;69(1):e44. doi: 10.1192/j.eurpsy.2026.10179 (PMC13122530; doi:10.1192/j.eurpsy.2026.10179)
Supplement: Quadackers et al. supplementary material [file S0924933826101795sup001.zip › Supplementary Table 2.docx]

**Supplementary Table 2**

| Imputa- tion nr. | model | gender | n | events | AUC (SE) | CITL  (SE) | Slope (SE) | Brier (SE) | Nagelkerke R^2^ (SE) |
| --- | --- | --- | --- | --- | --- | --- | --- | --- | --- |
| 1 | Full | Men | 983 | 302 | 0.66 (0.02) | 1.22 (0.07) | 0.74 (0.09) | 0.23 (0.01) | 0.10 (0.03) |
| 1 | Partial | Men | 983 | 302 | 0.63 (0.02) | 0.84 (0.07) | 0.59 (0.09) | 0.22 (0.01) | 0.06 (0.02) |
| 1 | Full | Females | 381 | 95 | 0.72 (0.03) | 1.18 (0.13) | 0.94 (0.15) | 0.19 (0.02) | 0.18 (0.05) |
| 1 | Partial | Females | 381 | 95 | 0.71 (0.03) | 0.65 (0.13) | 0.82 (0.14) | 0.17 (0.01) | 0.15 (0.04) |
| 2 | Full | Men | 983 | 306 | 0.68 (0.02) | 1.25 (0.07) | 0.80 (0.09) | 0.23 (0.01) | 0.12 (0.02) |
| 2 | Partial | Men | 983 | 306 | 0.64 (0.02) | 0.86 (0.07) | 0.69 (0.10) | 0.22 (0.01) | 0.08 (0.02) |
| 2 | Full | Females | 381 | 95 | 0.70 (0.03) | 1.20 (0.13) | 0.81 (0.14) | 0.19 (0.02) | 0.14 (0.04) |
| 2 | Partial | Females | 381 | 95 | 0.69 (0.03) | 0.66 (0.13) | 0.74 (0.13) | 0.18 (0.01) | 0.13 (0.04) |
| 3 | Full | Men | 983 | 305 | 0.67 (0.02) | 1.25 (0.07) | 0.79 (0.09) | 0.23 (0.01) | 0.11 (0.02) |
| 3 | Partial | Men | 983 | 305 | 0.64 (0.02) | 0.85 (0.07) | 0.68 (0.10) | 0.22 (0.01) | 0.08 (0.02) |
| 3 | Full | Females | 381 | 95 | 0.72 (0.03) | 1.19 (0.13) | 0.87 (0.14) | 0.19 (0.02) | 0.16 (0.04) |
| 3 | Partial | Females | 381 | 95 | 0.70 (0.03) | 0.66 (0.13) | 0.79 (0.14) | 0.18 (0.01) | 0.14 (0.04) |
| 4 | Full | Men | 983 | 295 | 0.68 (0.02) | 1.20 (0.07) | 0.80 (0.09) | 0.23 (0.01) | 0.11 (0.02) |
| 4 | Partial | Men | 983 | 295 | 0.64 (0.02) | 0.80 (0.07) | 0.64 (0.10) | 0.22 (0.01) | 0.07 (0.02) |
| 4 | Full | Females | 381 | 94 | 0.72 (0.03) | 1.17 (0.13) | 0.90 (0.14) | 0.19 (0.02) | 0.17 (0.04) |
| 4 | Partial | Females | 381 | 94 | 0.70 (0.03) | 0.64 (0.13) | 0.75 (0.13) | 0.18 (0.01) | 0.13 (0.04) |
| 5 | Full | Men | 983 | 298 | 0.68 (0.02) | 1.20 (0.07) | 0.76 (0.09) | 0.23 (0.01) | 0.11 (0.02) |
| 5 | Partial | Men | 983 | 298 | 0.64 (0.02) | 0.82 (0.07) | 0.68 (0.10) | 0.22 (0.01) | 0.08 (0.02) |
| 5 | Full | Females | 381 | 96 | 0.73 (0.03) | 1.20 (0.13) | 1.00 (0.15) | 0.19 (0.02) | 0.19 (0.04) |
| 5 | Partial | Females | 381 | 96 | 0.73 (0.03) | 0.67 (0.13) | 0.95 (0.14) | 0.17 (0.01) | 0.19 (0.05) |
| 6 | Full | Men | 983 | 302 | 0.69 (0.02) | 1.21 (0.07) | 0.85 (0.09) | 0.23 (0.01) | 0.13 (0.02) |
| 6 | Partial | Men | 983 | 302 | 0.65 (0.02) | 0.83 (0.07) | 0.74 (0.10) | 0.22 (0.01) | 0.09 (0.02) |
| 6 | Full | Females | 381 | 90 | 0.71 (0.03) | 1.08 (0.13) | 0.83 (0.14) | 0.18 (0.02) | 0.15 (0.04) |
| 6 | Partial | Females | 381 | 90 | 0.70 (0.03) | 0.57 (0.13) | 0.76 (0.14) | 0.17 (0.01) | 0.13 (0.04) |
| 7 | Full | Men | 983 | 299 | 0.68 (0.02) | 1.20 (0.07) | 0.81 (0.09) | 0.23 (0.01) | 0.12 (0.03) |
| 7 | Partial | Men | 983 | 299 | 0.65 (0.02) | 0.82 (0.07) | 0.72 (0.10) | 0.22 (0.01) | 0.09 (0.02) |
| 7 | Full | Females | 381 | 94 | 0.72 (0.03) | 1.15 (0.13) | 0.87 (0.14) | 0.19 (0.02) | 0.16 (0.04) |
| 7 | Partial | Females | 381 | 94 | 0.71 (0.03) | 0.64 (0.13) | 0.83 (0.14) | 0.17 (0.01) | 0.15 (0.05) |
| 8 | Full | Men | 983 | 312 | 0.68 (0.02) | 1.27 (0.07) | 0.80 (0.09) | 0.24 (0.01) | 0.12 (0.02) |
| 8 | Partial | Men | 983 | 312 | 0.65 (0.02) | 0.89 (0.07) | 0.74 (0.10) | 0.22 (0.01) | 0.09 (0.02) |
| 8 | Full | Females | 381 | 95 | 0.74 (0.03) | 1.19 (0.13) | 0.98 (0.15) | 0.19 (0.02) | 0.19 (0.05) |
| 8 | Partial | Females | 381 | 95 | 0.72 (0.03) | 0.65 (0.13) | 0.87 (0.14) | 0.17 (0.01) | 0.17 (0.05) |
| 9 | Full | Men | 983 | 310 | 0.68 (0.02) | 1.27 (0.07) | 0.83 (0.09) | 0.23 (0.01) | 0.13 (0.02) |
| 9 | Partial | Men | 983 | 310 | 0.65 (0.02) | 0.88 (0.07) | 0.75 (0.10) | 0.22 (0.01) | 0.09 (0.02) |
| 9 | Full | Females | 381 | 95 | 0.73 (0.03) | 1.18 (0.13) | 1.01 (0.15) | 0.18 (0.02) | 0.20 (0.05) |
| 9 | Partial | Females | 381 | 95 | 0.72 (0.03) | 0.66 (0.13) | 0.93 (0.14) | 0.17 (0.01) | 0.18 (0.04) |
| 10 | Full | Men | 983 | 304 | 0.68 (0.02) | 1.23 (0.07) | 0.80 (0.09) | 0.23 (0.01) | 0.12 (0.02) |
| 10 | Partial | Men | 983 | 304 | 0.64 (0.02) | 0.84 (0.07) | 0.68 (0.10) | 0.22 (0.01) | 0.08 (0.02) |
| 10 | Full | Females | 381 | 92 | 0.72 (0.03) | 1.14 (0.13) | 0.93 (0.15) | 0.18 (0.02) | 0.17 (0.05) |
| 10 | Partial | Females | 381 | 92 | 0.71 (0.03) | 0.60 (0.13) | 0.85 (0.14) | 0.17 (0.01) | 0.16 (0.05) |

## **Pooled metrics by gender (Rubin’s rules)**

| model | gender | AUC (SE) [95% CI] | CITL (SE) [95% CI] | Slope (SE) [95% CI] | Brier (SE) [95% CI] | Nagelkerke R^2^ (SE) [95% CI] |
| --- | --- | --- | --- | --- | --- | --- |
| Full | Men | 0.68 (0.02) [0.64; 0.72] | 1.23 (0.08) [1.07; 1.39] | 0.80 (0.10) [0.61; 0.99] | 0.23 (0.01) [0.21; 0.25] | 0.12 (0.03) [0.07; 0.16] |
| Partial | Men | 0.64 (0.02) [0.60; 0.69] | 0.84 (0.08) [0.69; 1.00] | 0.69 (0.11) [0.47; 0.91] | 0.22 (0.01) [0.20; 0.24] | 0.08 (0.02) [0.03; 0.12] |
| Full | Females | 0.72 (0.03) [0.66; 0.78] | 1.17 (0.13) [0.91; 1.43] | 0.91 (0.16) [0.59; 1.23] | 0.19 (0.02) [0.15; 0.22] | 0.17 (0.05) [0.07; 0.27] |
| Partial | Females | 0.71 (0.03) [0.64; 0.78] | 0.64 (0.13) [0.38; 0.90] | 0.83 (0.16) [0.52; 1.14] | 0.17 (0.01) [0.14; 0.20] | 0.15 (0.05) [0.05; 0.25] |

**Supplementary Table 2.** Sex-stratified predictive model performances, including pooled metrics using Rubin’s rules. AUC = Area under the Curve (= c-statistic), CITL = calibration-in-the-large. SE = standard error. CI = confidence interval.
