# Supplementary material for: External validation and recalibration of the psychosis metabolic risk calculator (PsyMetRiC) in young adults with chronic psychotic disorders in the Netherlands
Source: Eur Psychiatry. 2026 Mar 9;69(1):e44. doi: 10.1192/j.eurpsy.2026.10179 (PMC13122530; doi:10.1192/j.eurpsy.2026.10179)
Supplement: Quadackers et al. supplementary material [file S0924933826101795sup001.zip › Supplementary Figure 2.docx]

**Supplementary Figure 2**

| **A. PHAMOUS** | Before calibration (primary analysis) | After calibration (PsyMetRiC-NL)* |
| --- | --- | --- |
| Full  model | 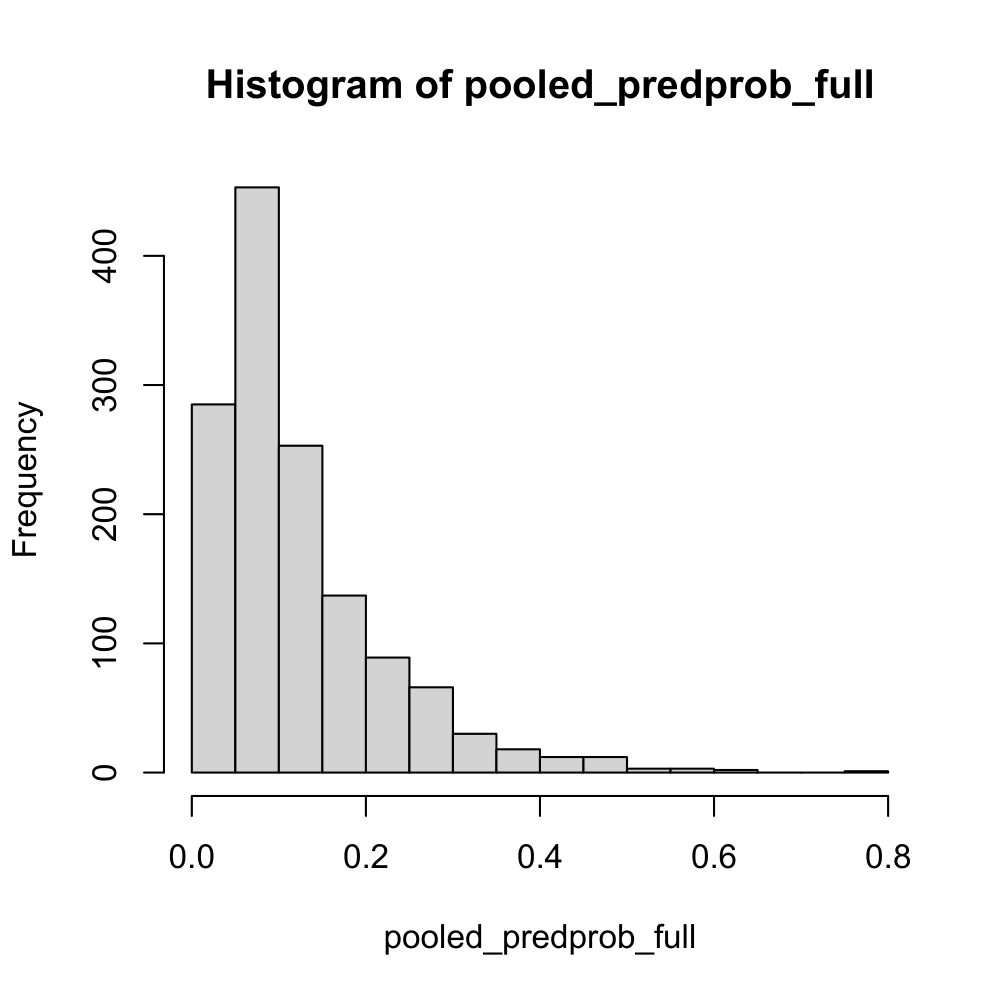 | 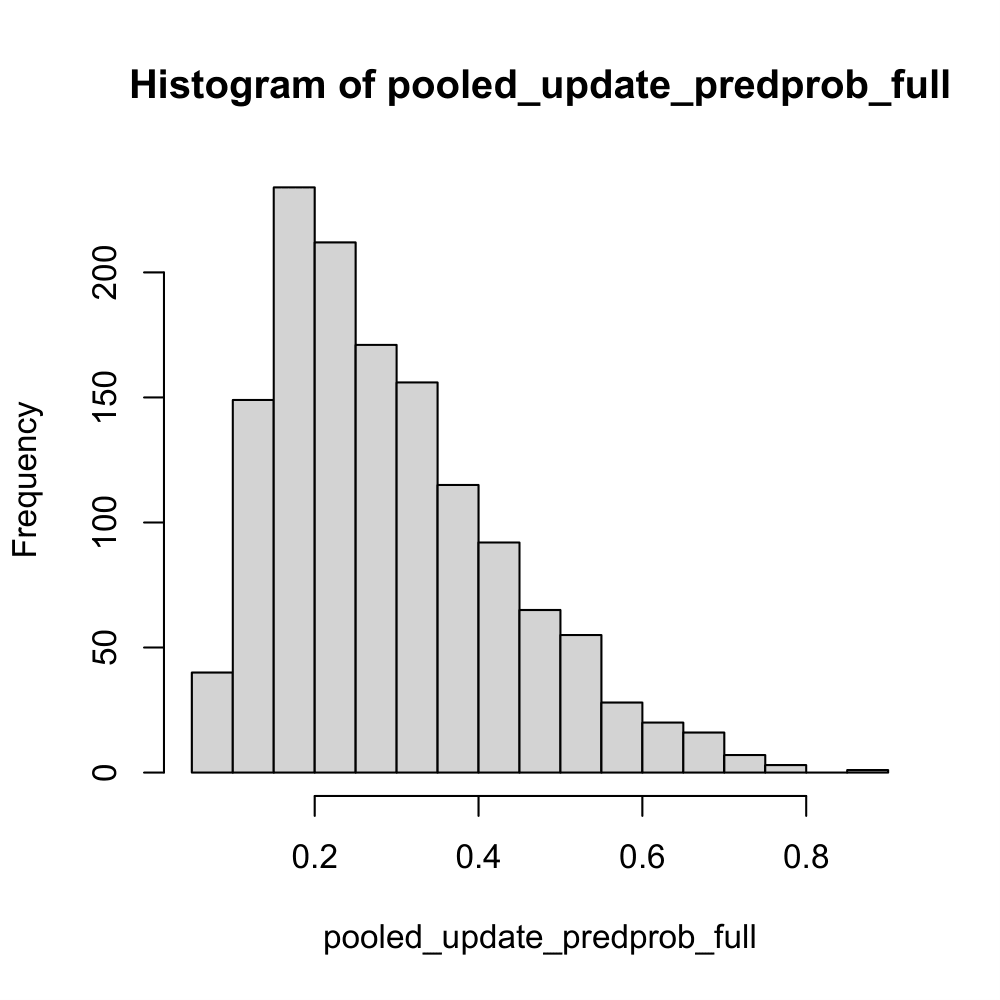 |
| Partial model | 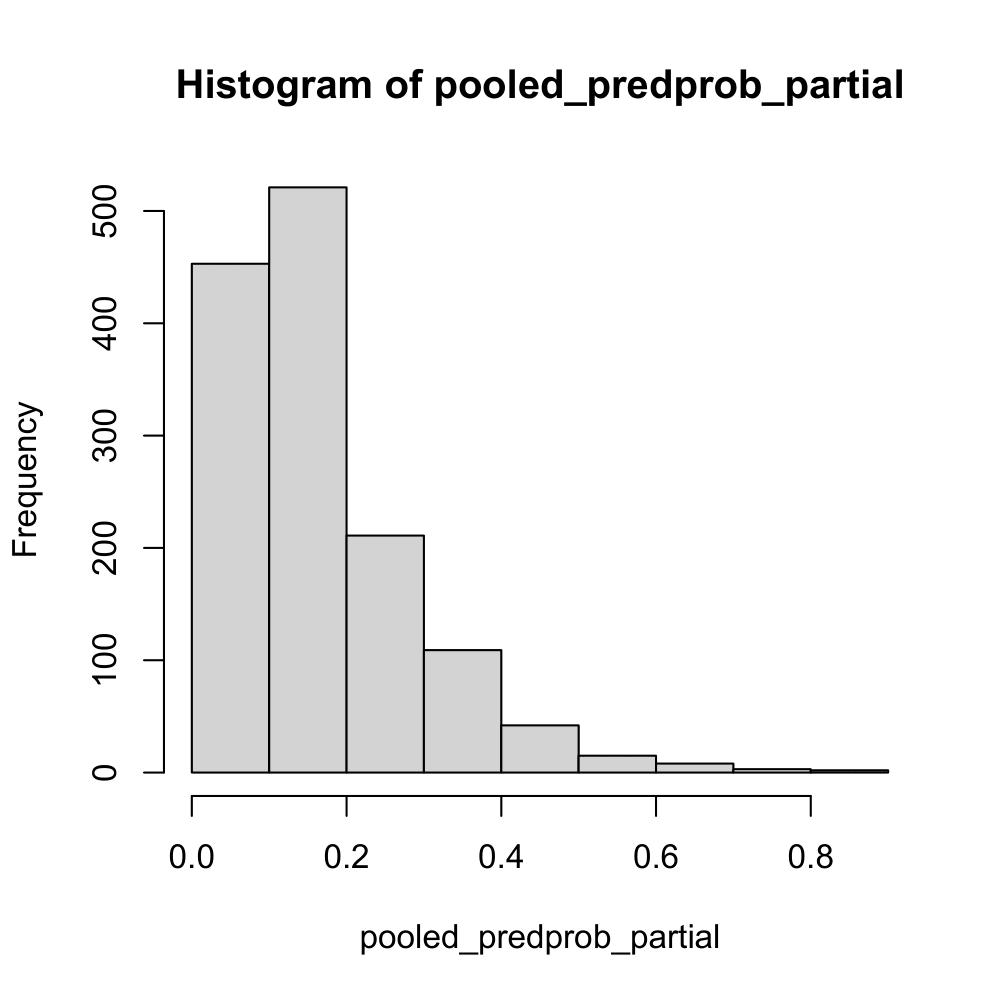 | 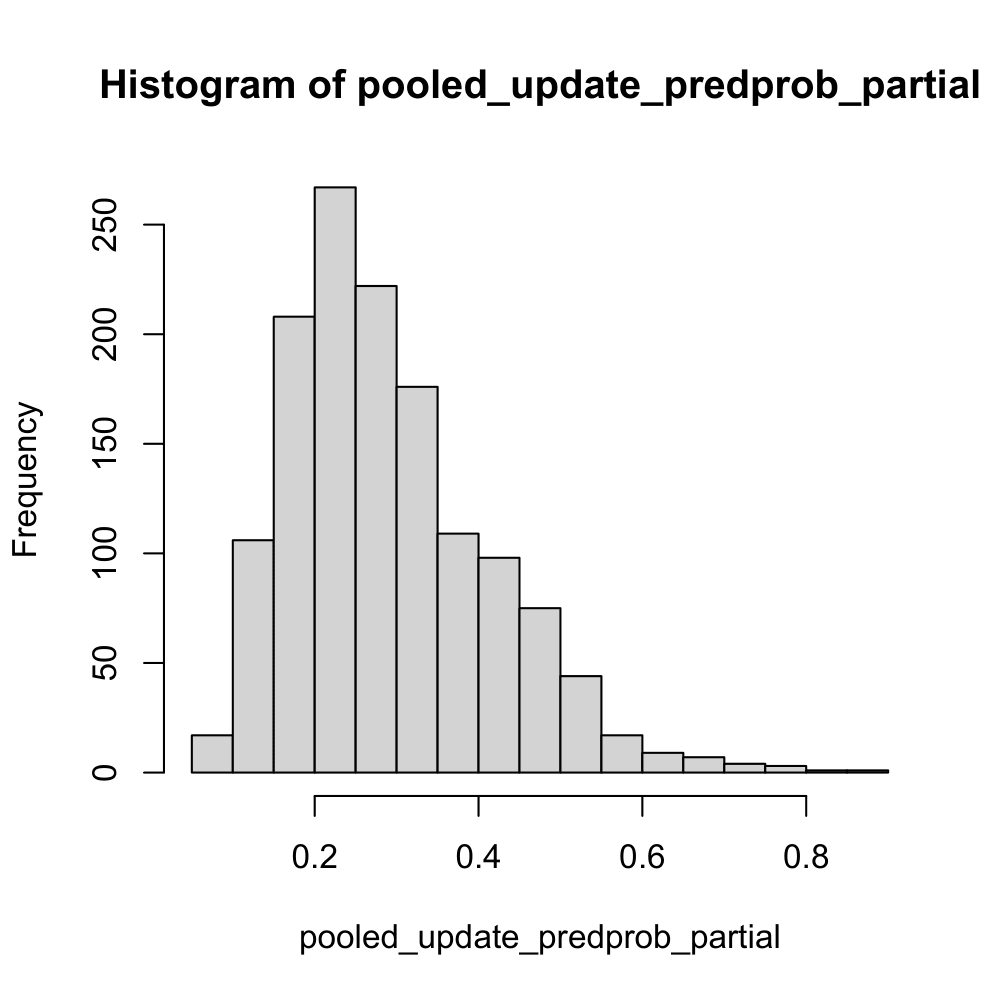 |

*After applying the updated LP intercept of -**4.506925**, and multiplying the ßs of the original model by **0.8377201** for the ‘full model’, respectively applying the updated LP intercept of **-4.806266** and original ß multiplication by **0.7436072** for the ‘partial model’, as explained in the table here below.

| **B. Development sample** | After coefficient shrinkage |
| --- | --- |
| Full  model | 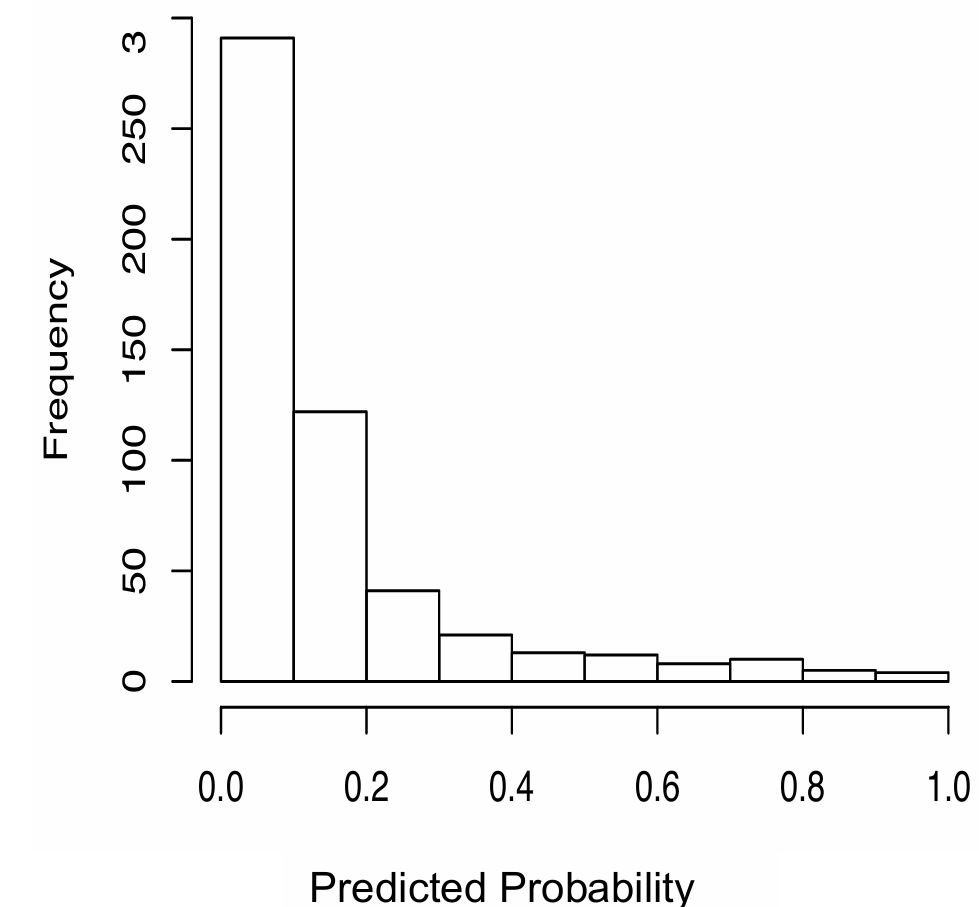 |
| Partial  model | 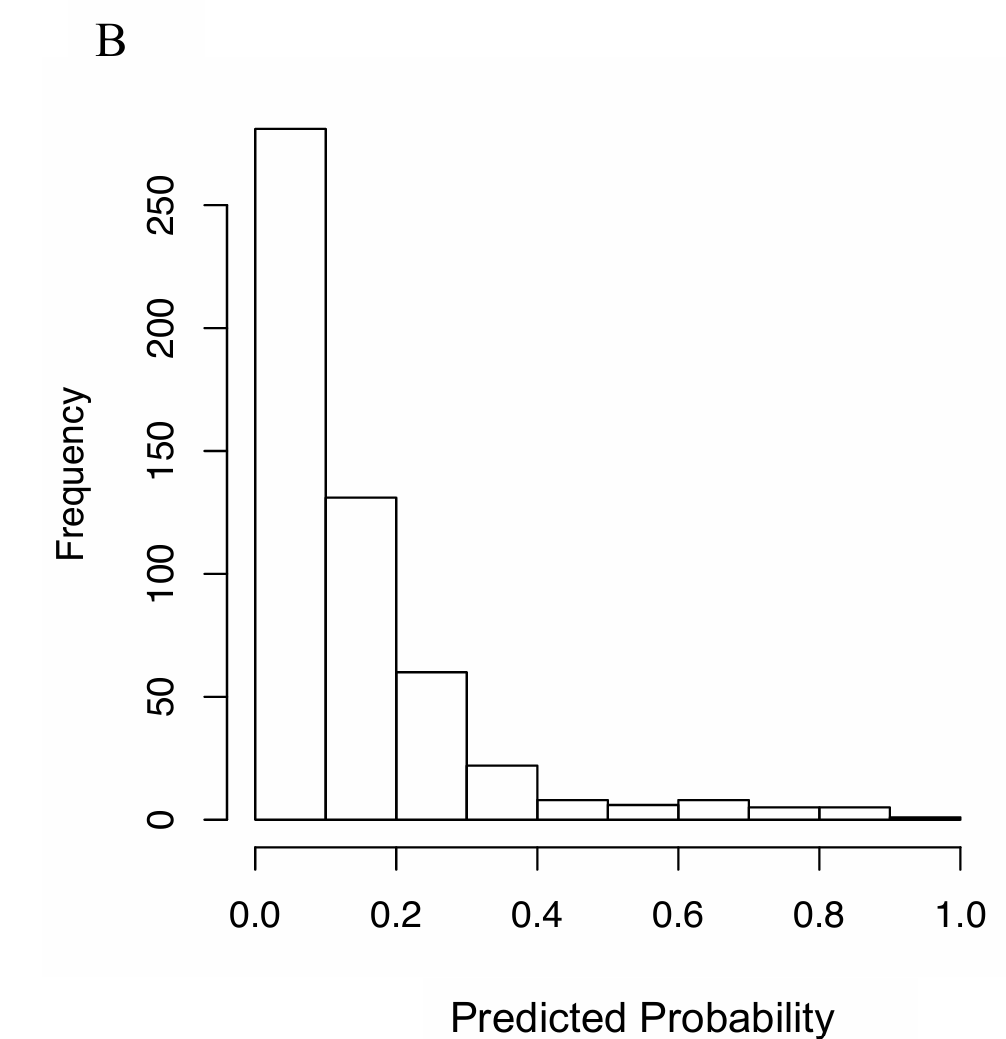 |

**Supplementary Figure 2** Pooled predicted probabilities and linear predictors for PsyMetRiC in PHAMOUS (A), and development sample (B) before and after calibration.
