# Supplementary material for: External validation and recalibration of the psychosis metabolic risk calculator (PsyMetRiC) in young adults with chronic psychotic disorders in the Netherlands
Source: Eur Psychiatry. 2026 Mar 9;69(1):e44. doi: 10.1192/j.eurpsy.2026.10179 (PMC13122530; doi:10.1192/j.eurpsy.2026.10179)
Supplement: Quadackers et al. supplementary material [file S0924933826101795sup001.zip › Supplementary Figure 3.docx]

**Supplementary Figure 3** (first 3 x 3-panel shows the full model before recalibration in the other 9 imputed datasets; the second 3 x 3-panel the partial model before recalibration, the third 3 x 3-panel the full model after recalibration, and the fourth 3 x 3-panel the partial model after recalibration)

**Before recalibration, full model**

| 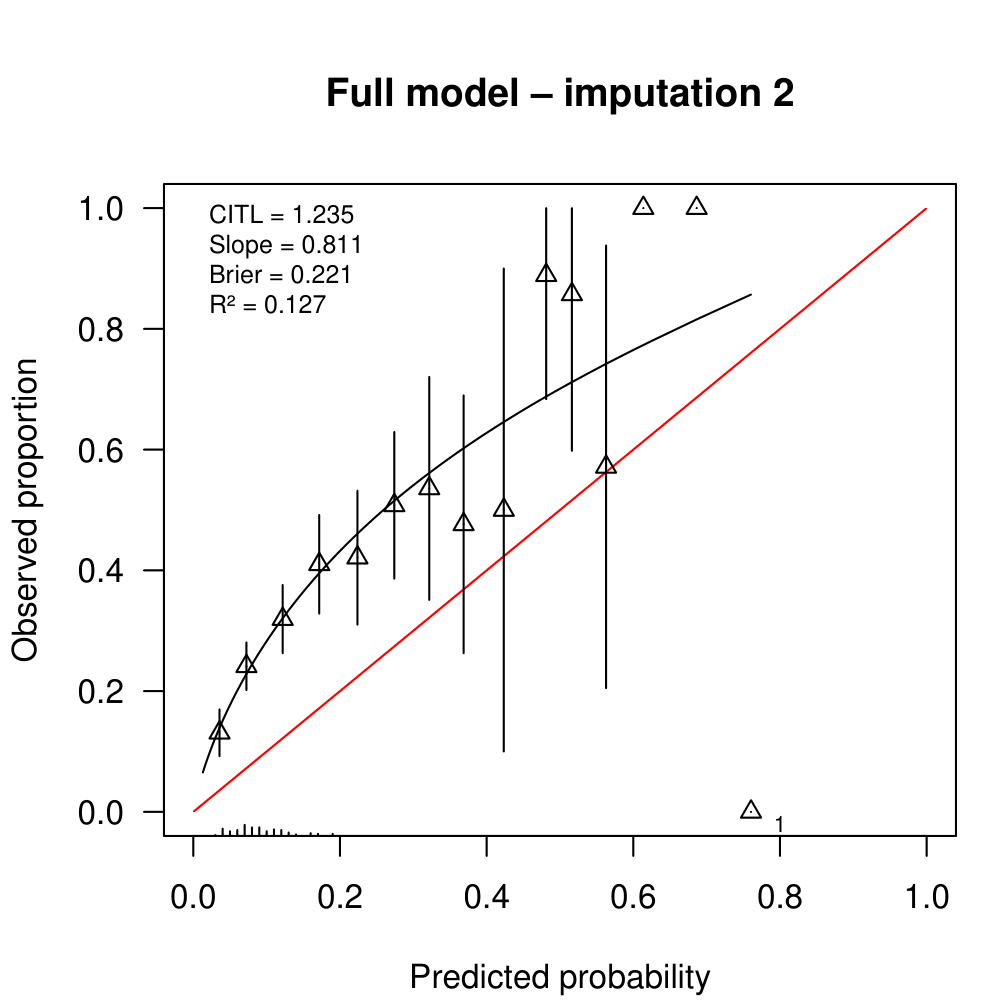 | 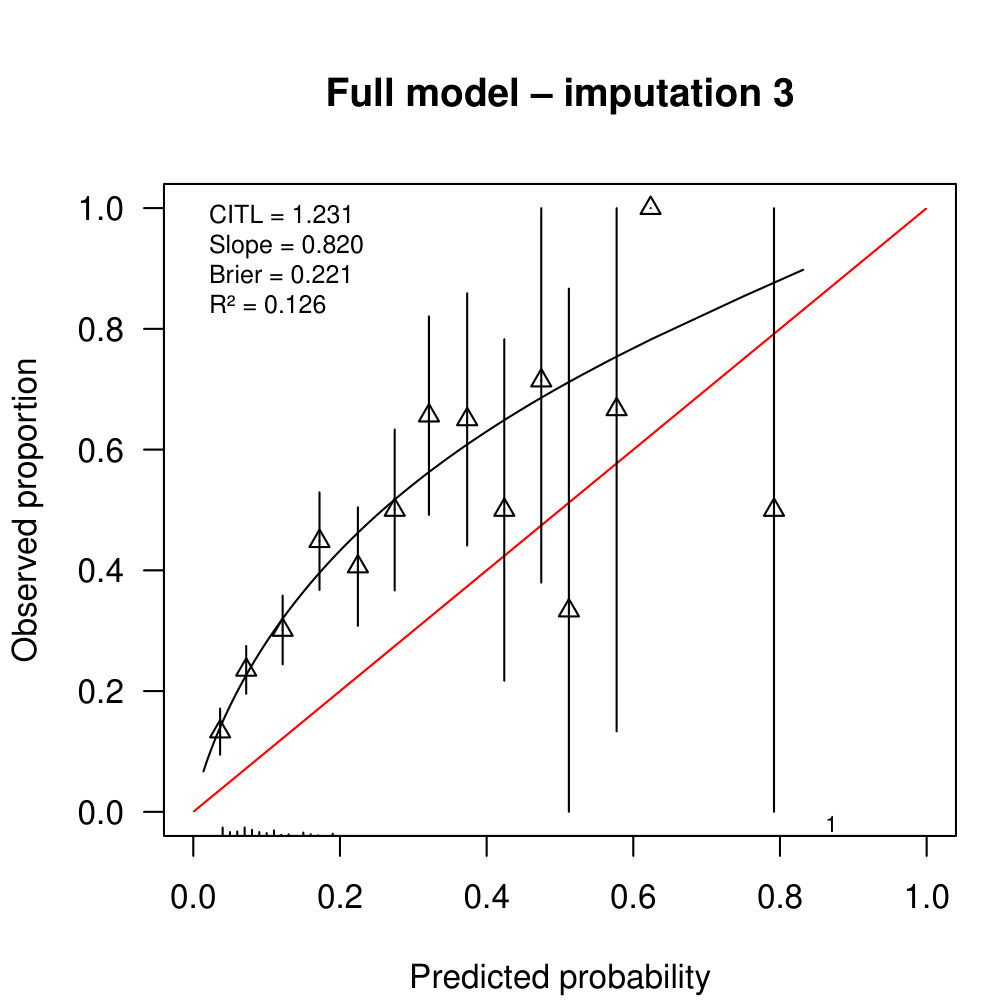 | 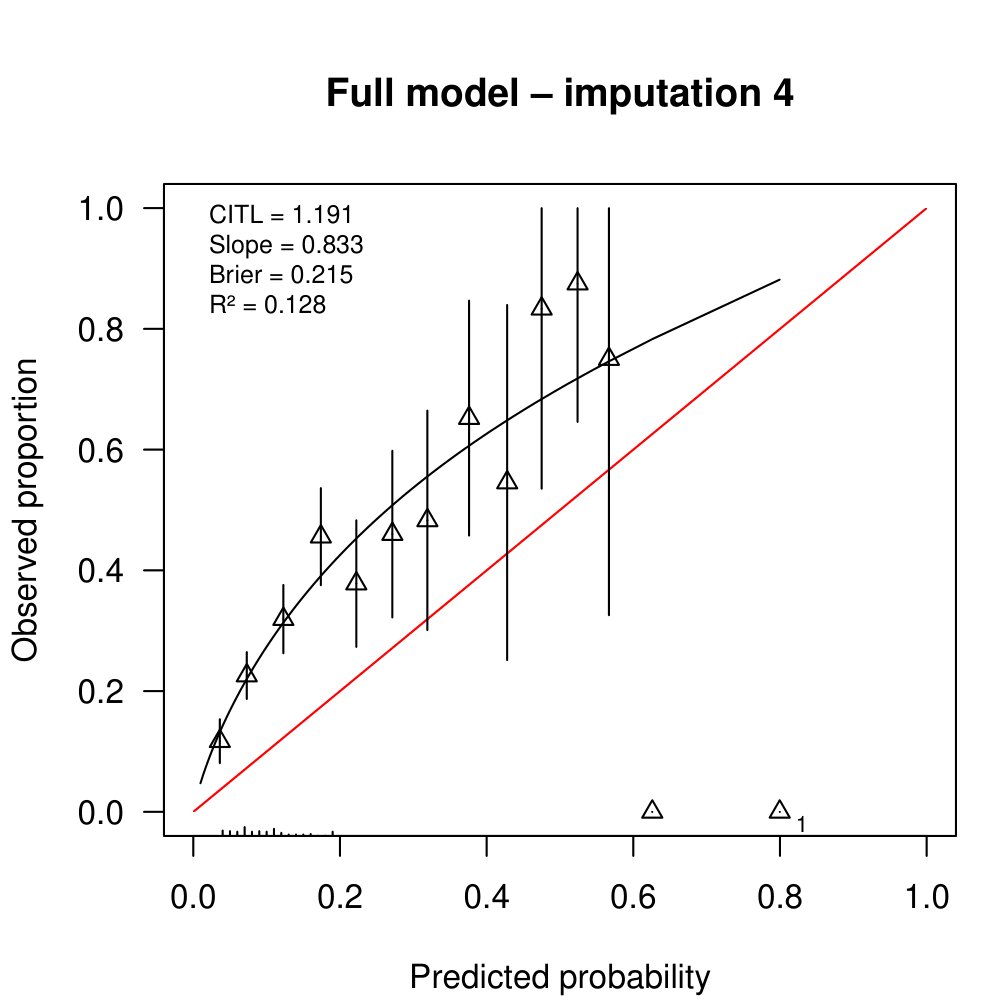 |
| --- | --- | --- |
| 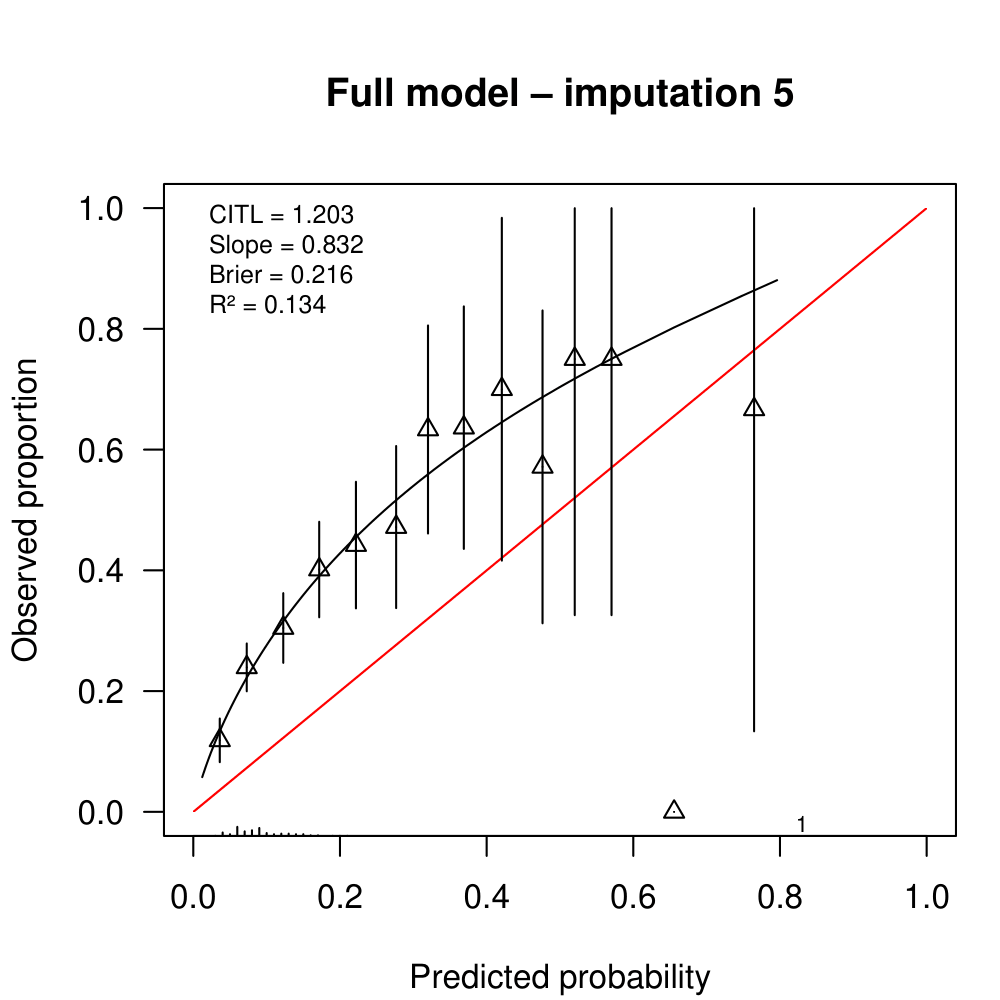 | 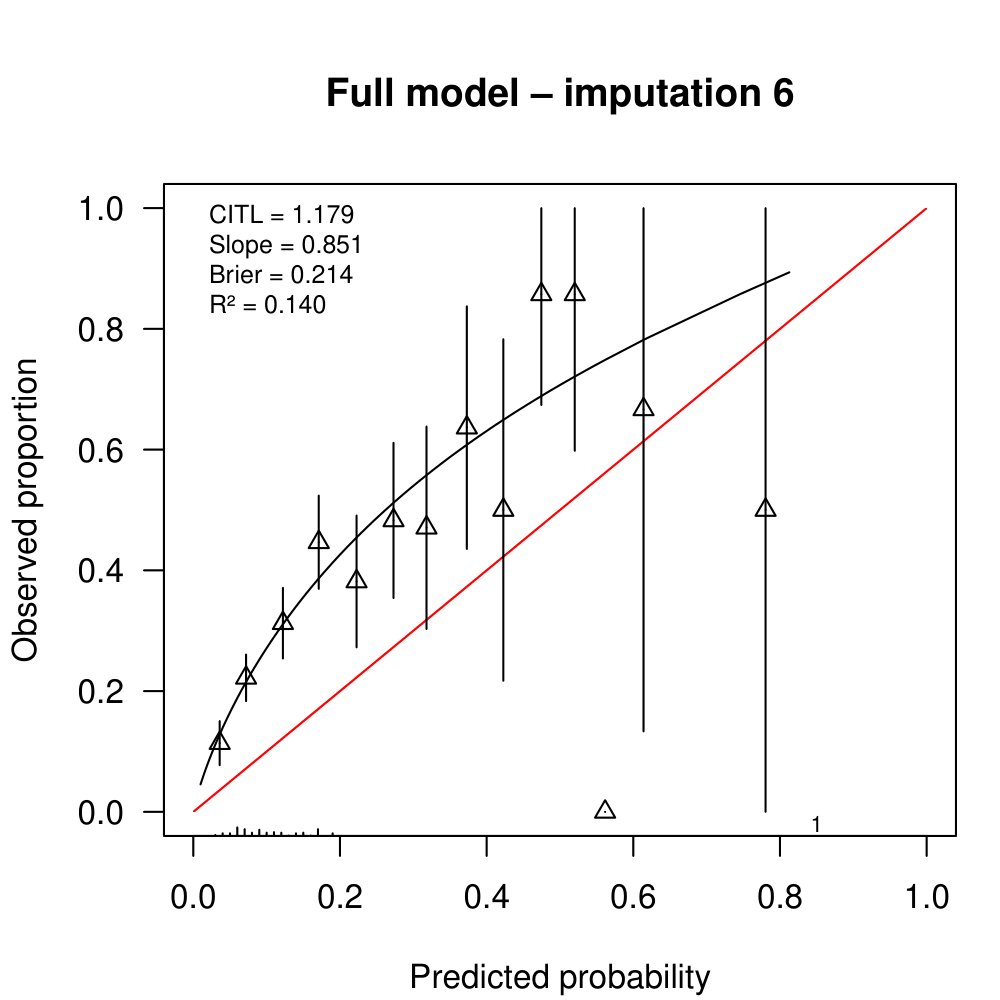 | 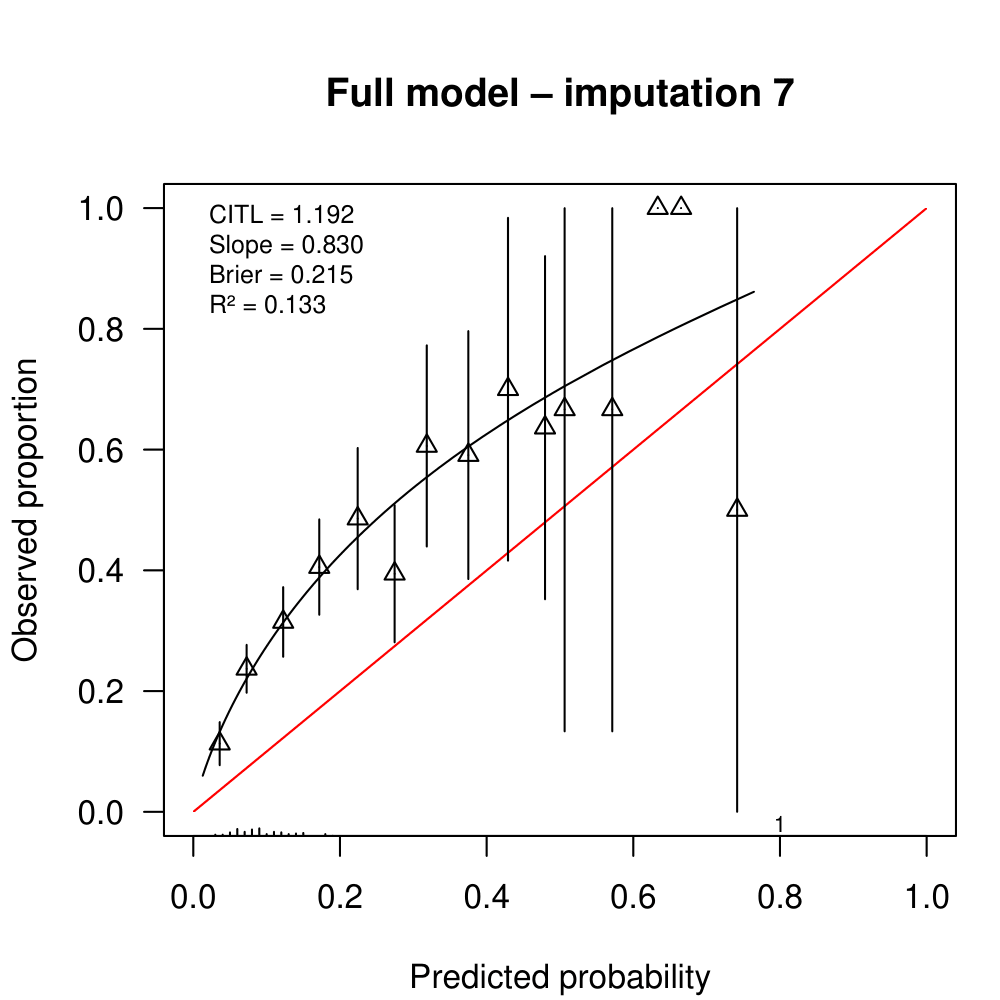 |
| 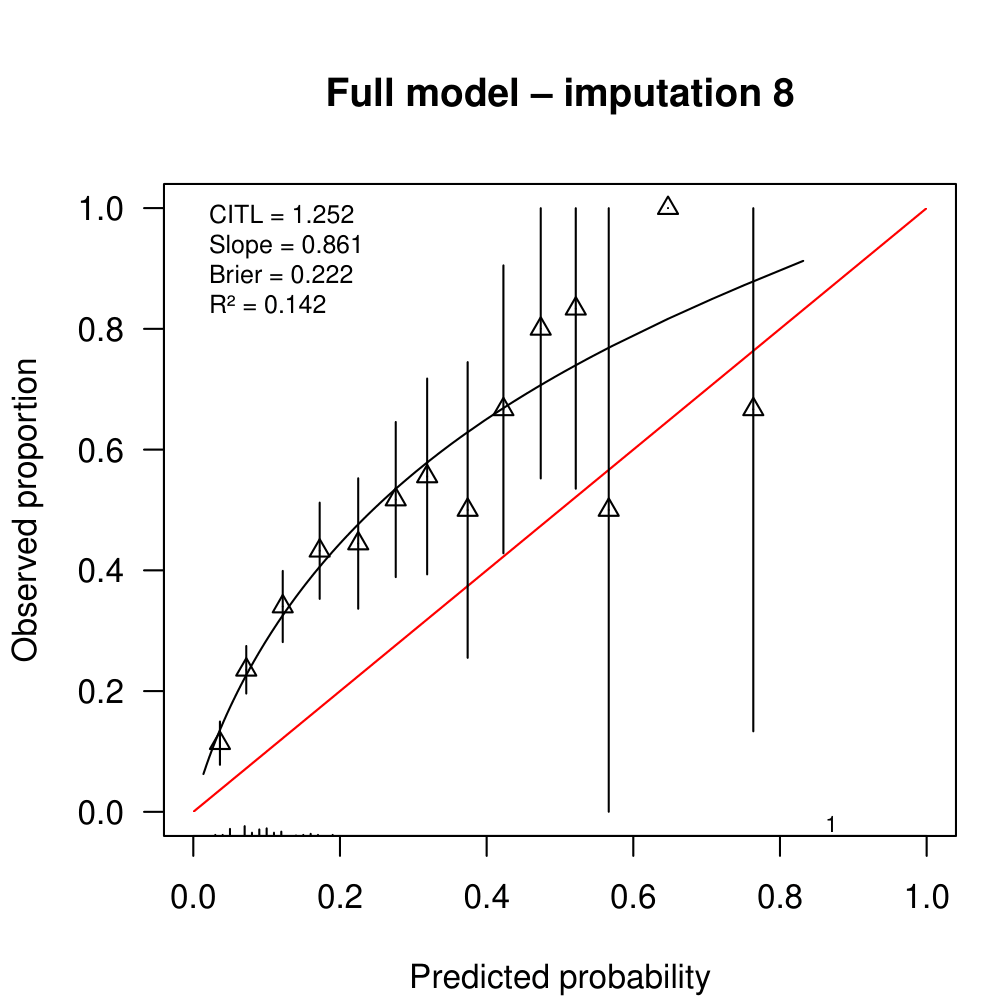 | 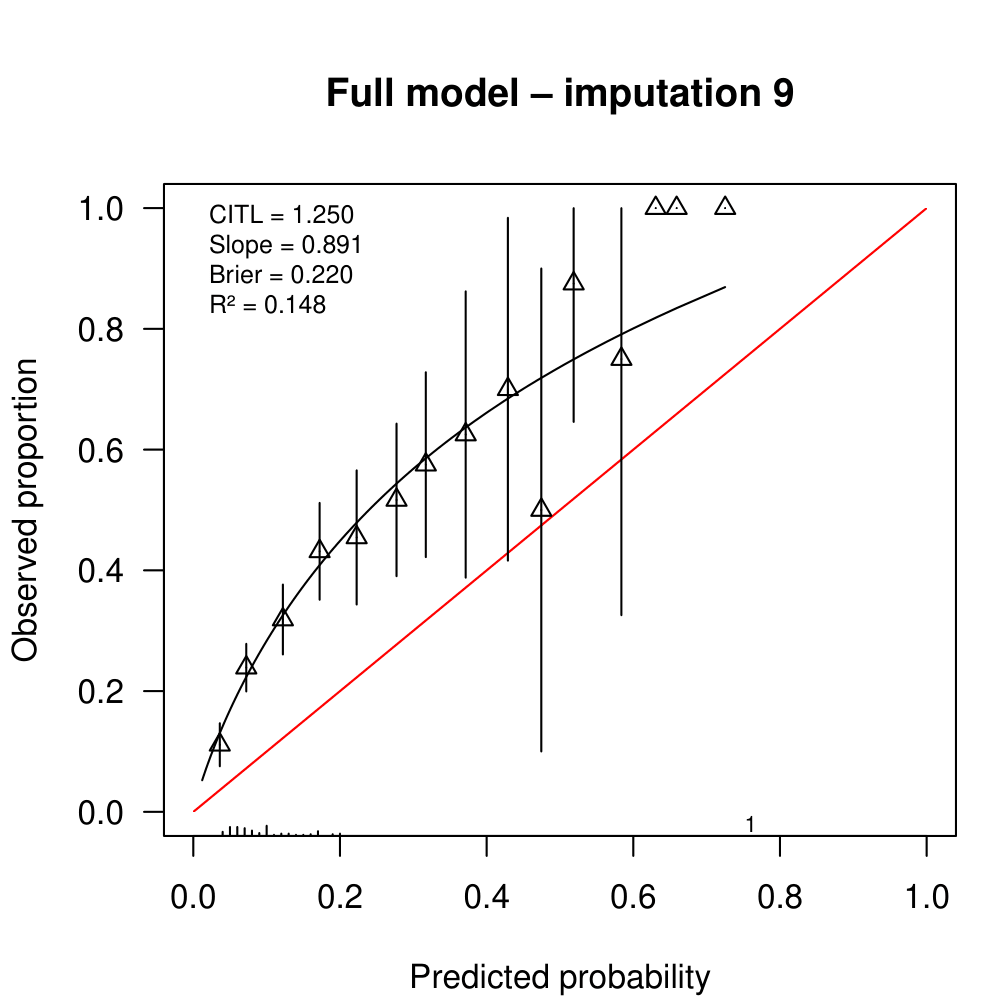 | 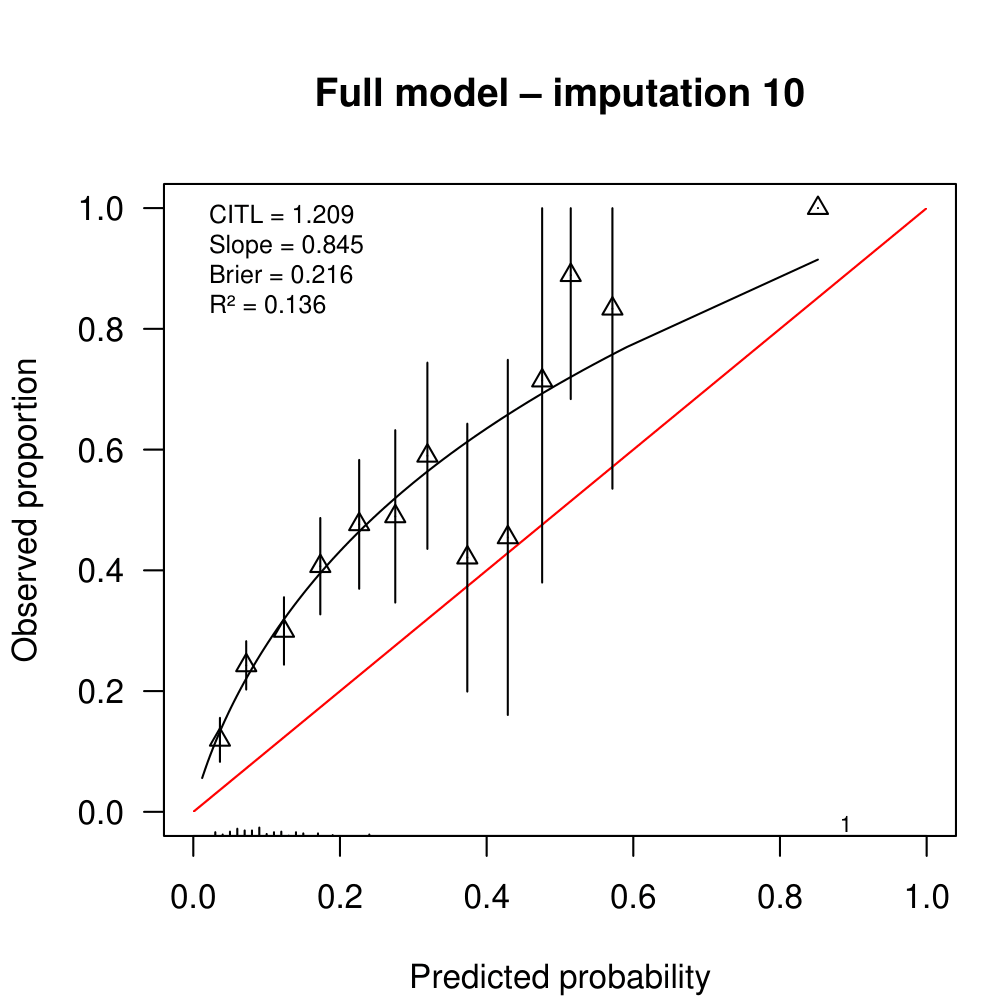 |

**Before recalibration, partial model**

| 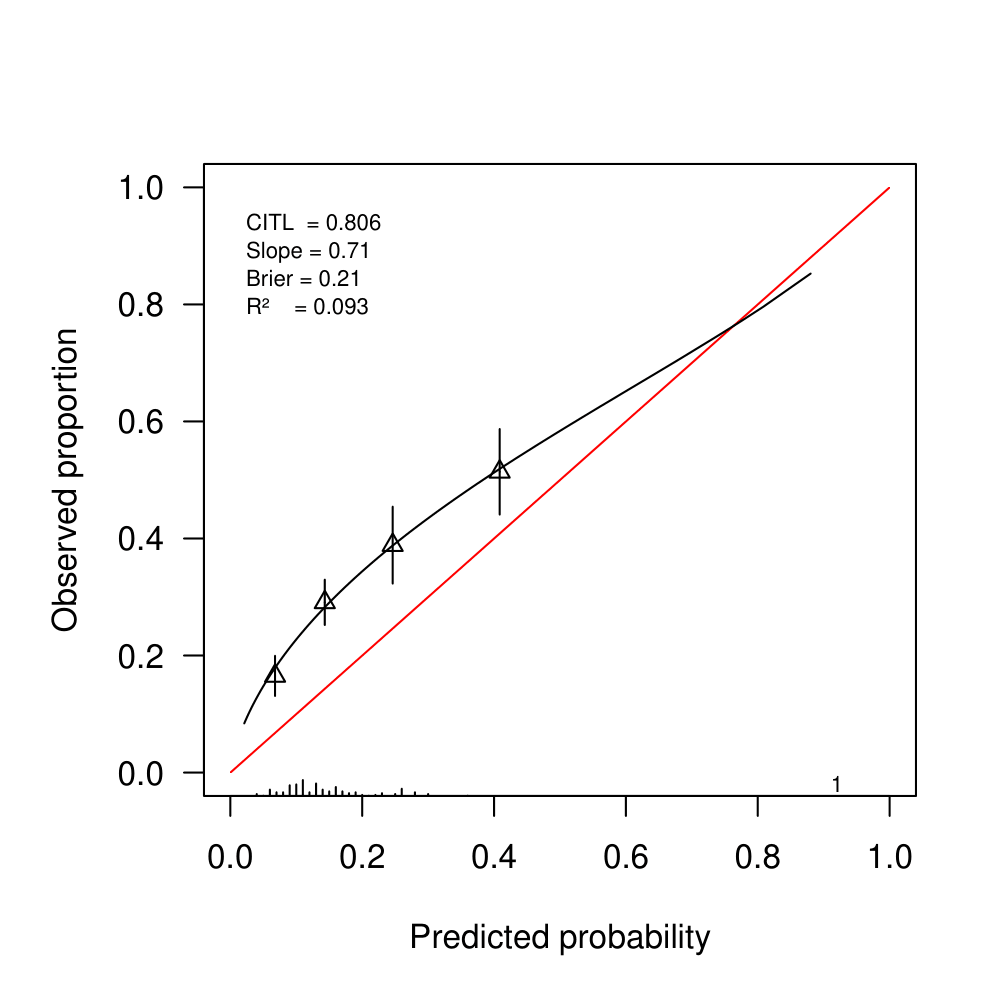 | 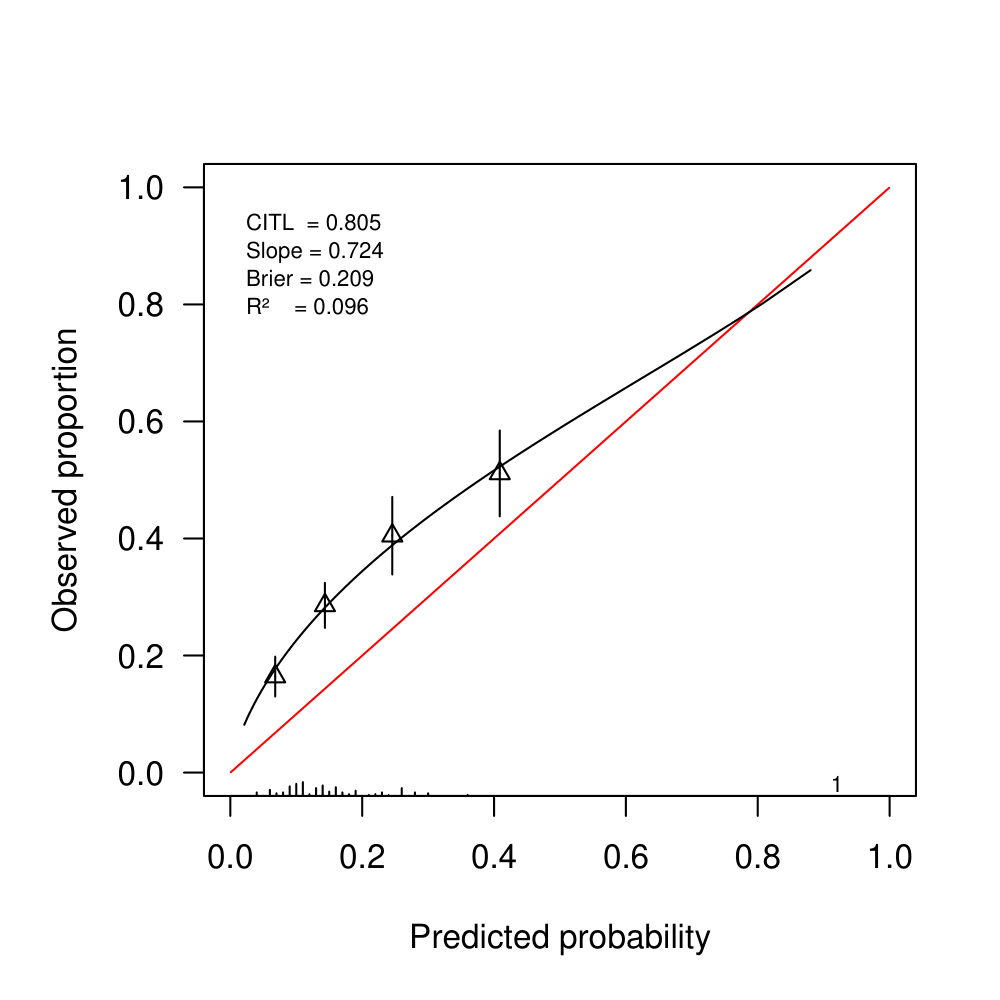 | 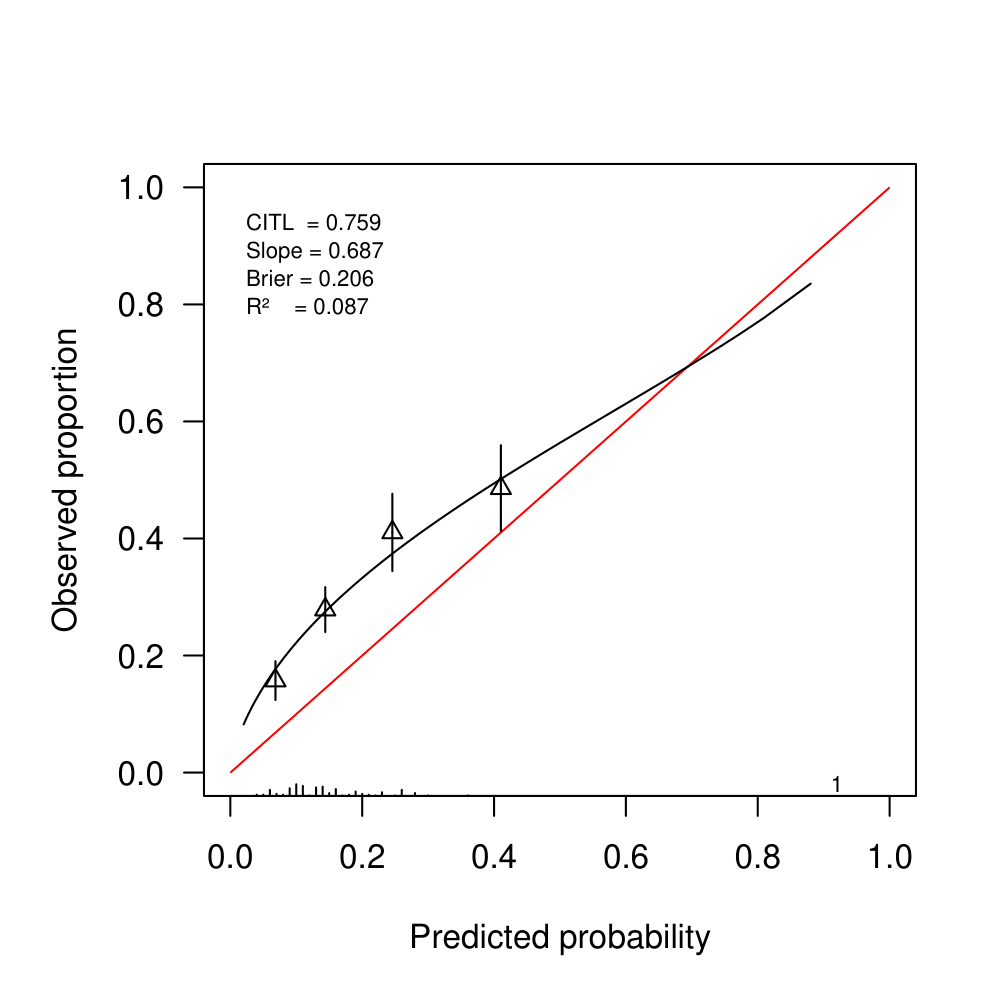 |
| --- | --- | --- |
| 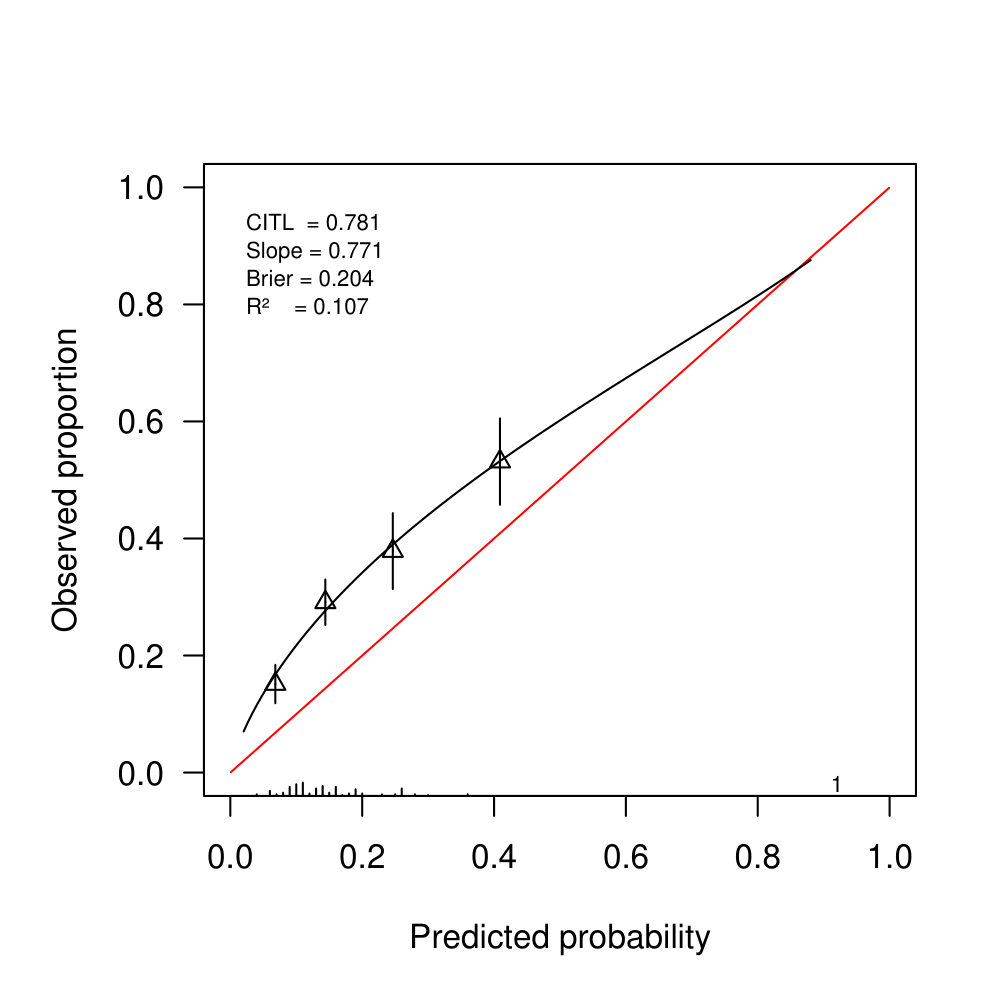 | 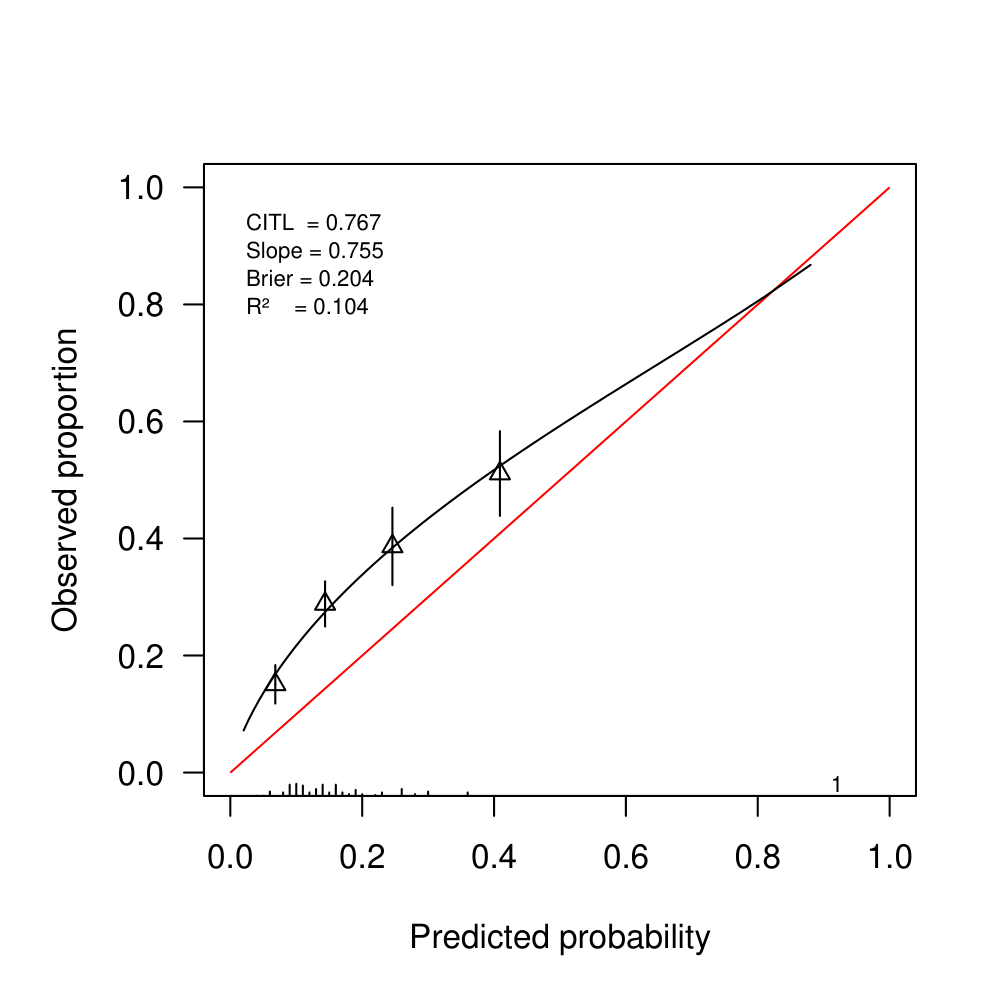 | 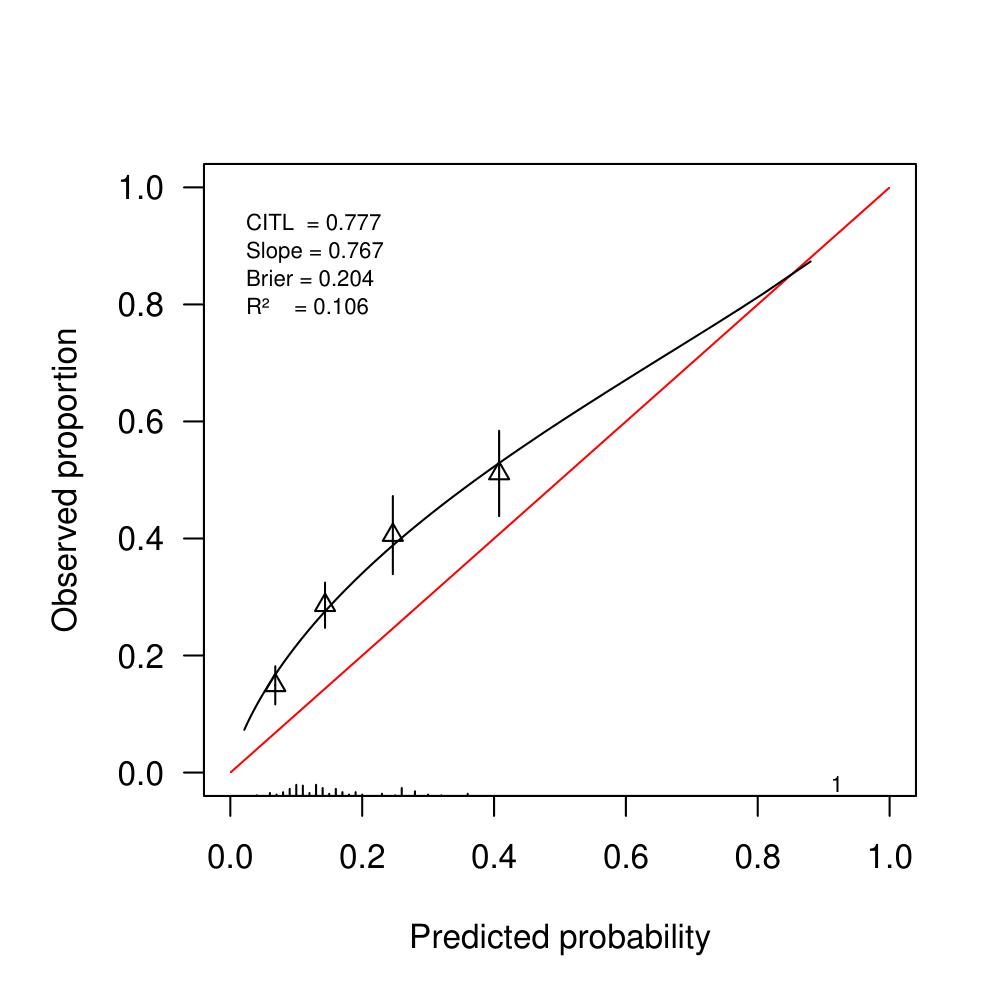 |
| 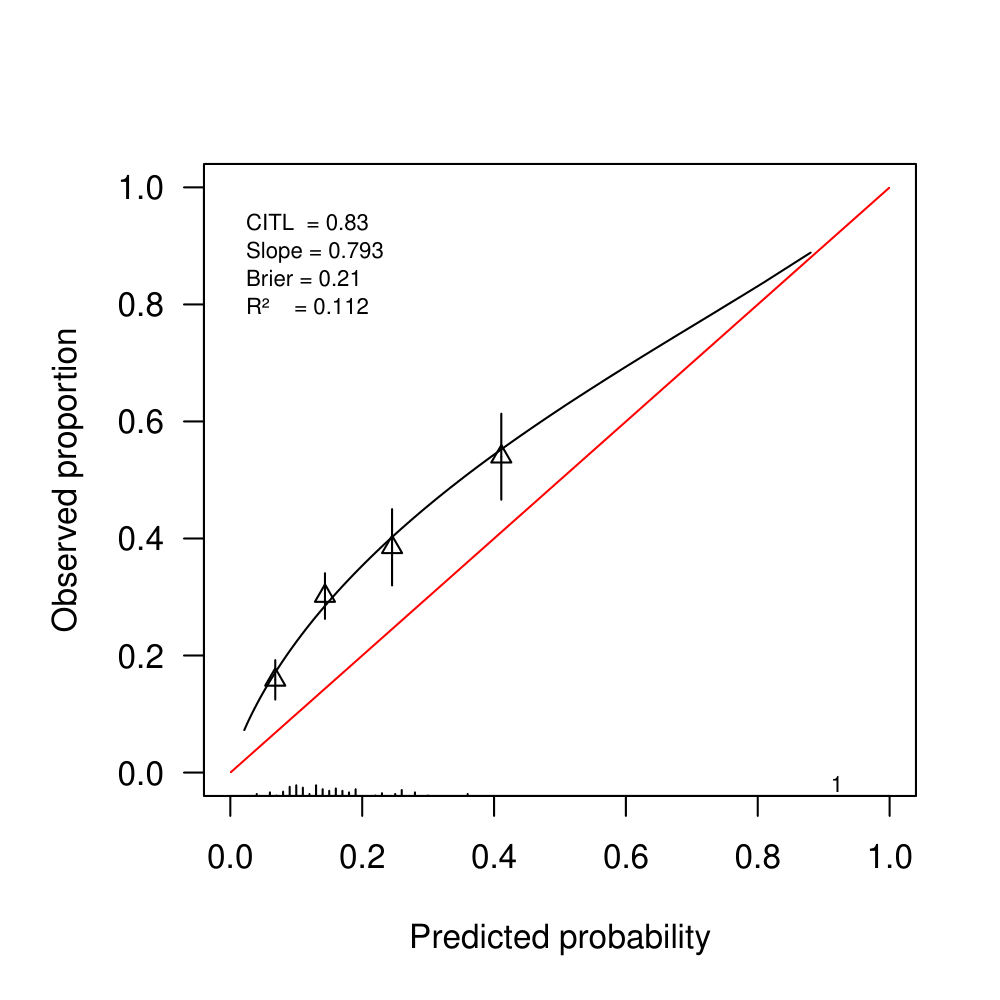 | 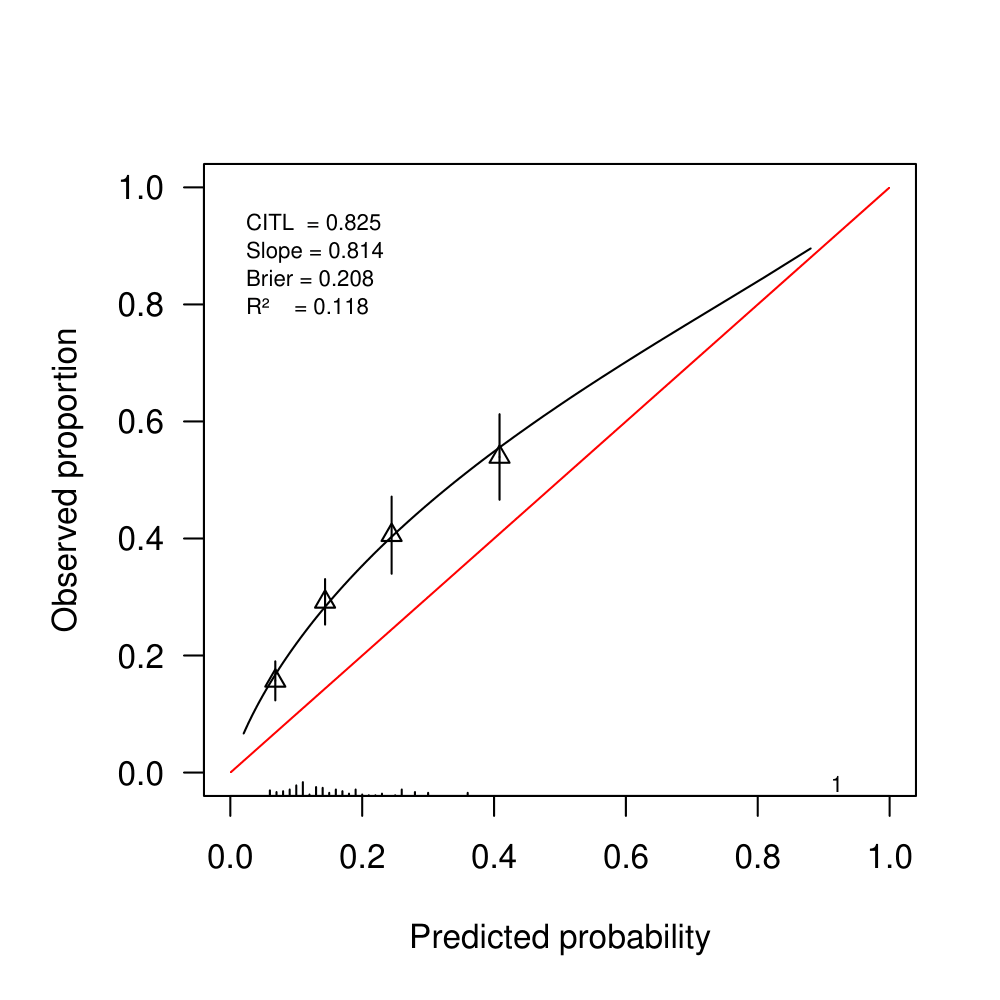 | 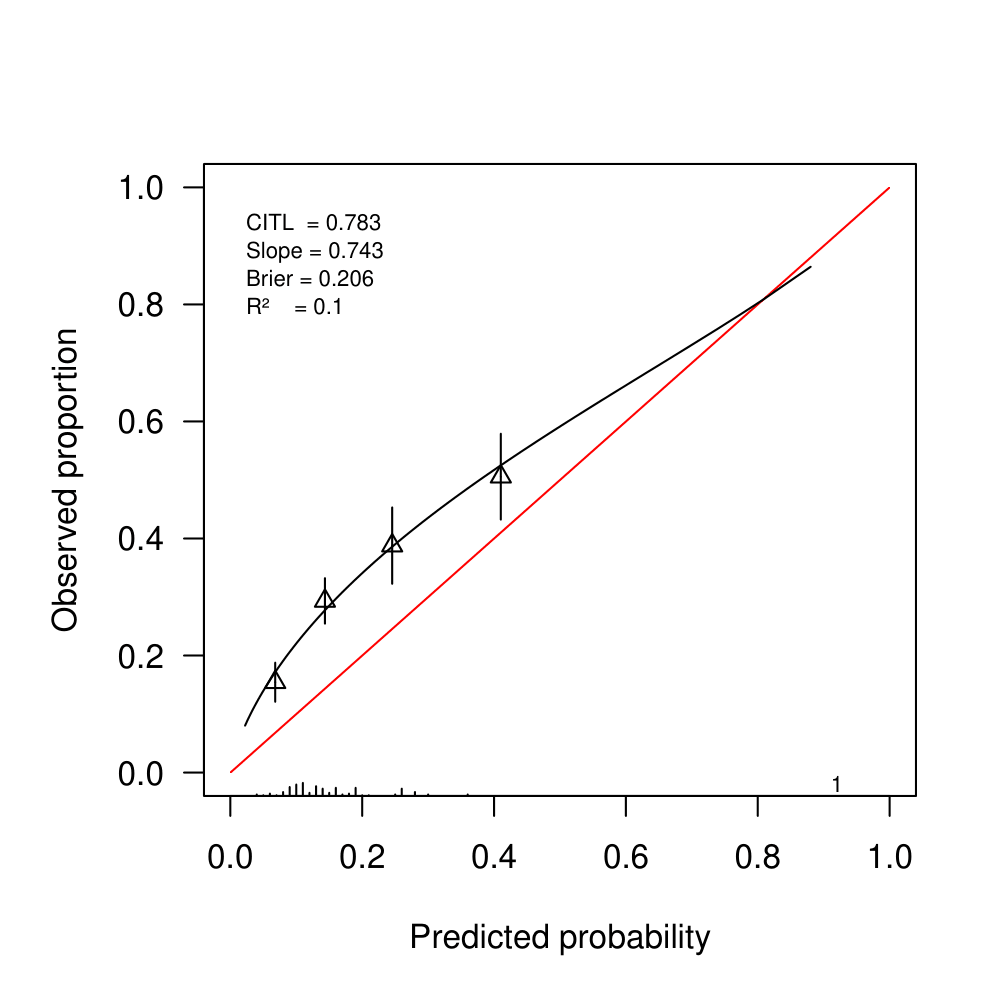 |

**After recalibration, full model**

| 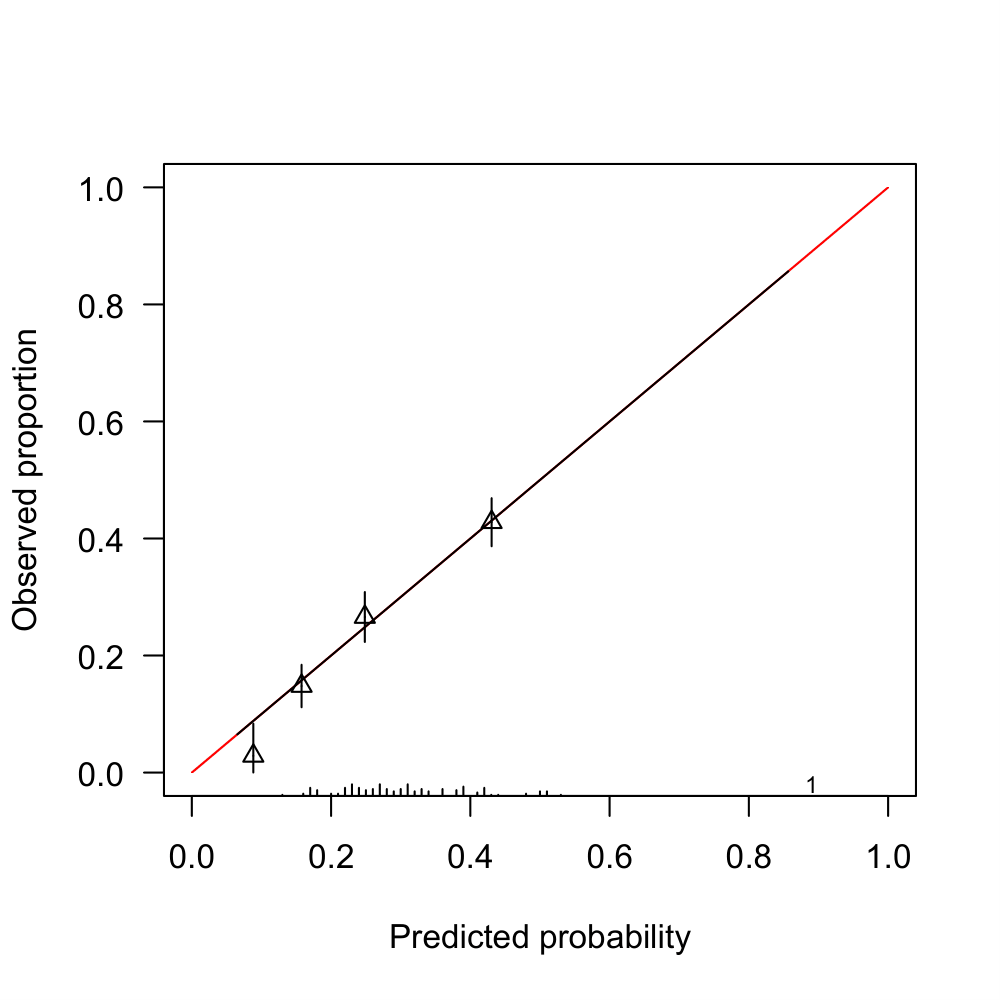 | 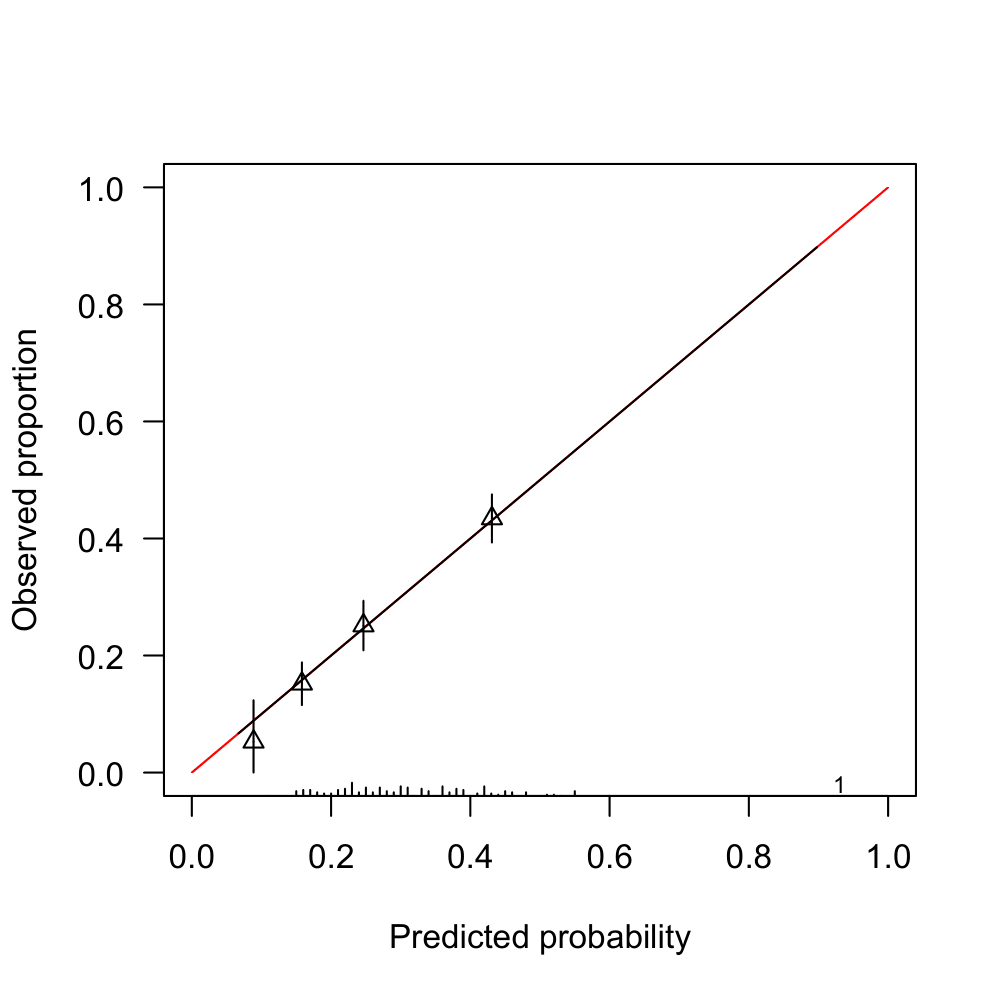 | 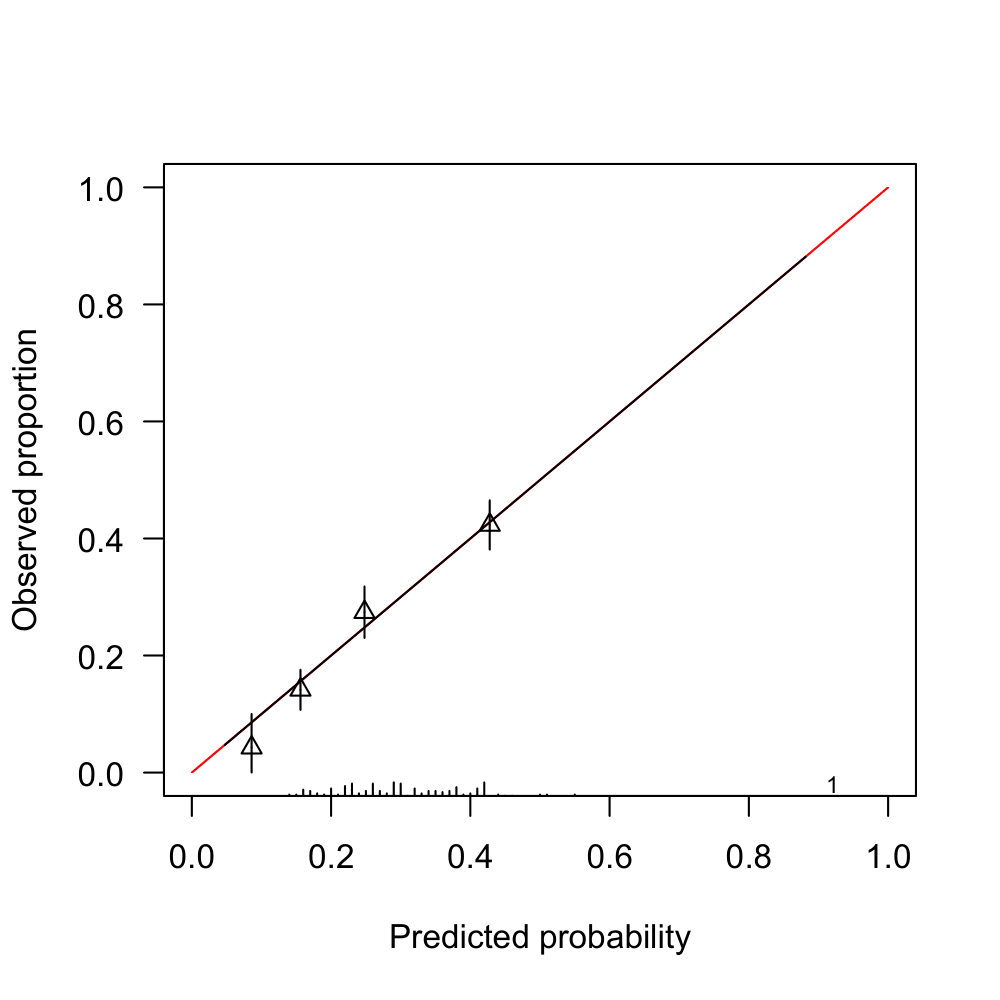 |
| --- | --- | --- |
| 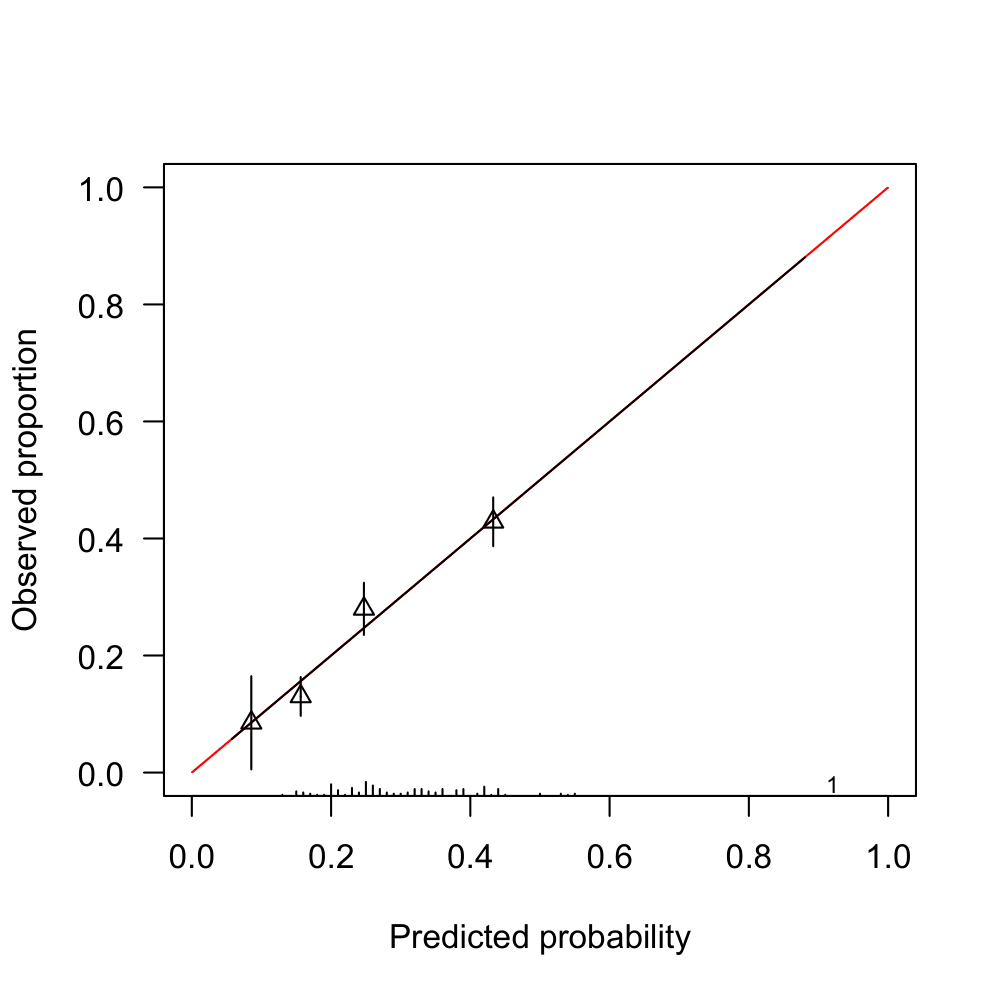 | 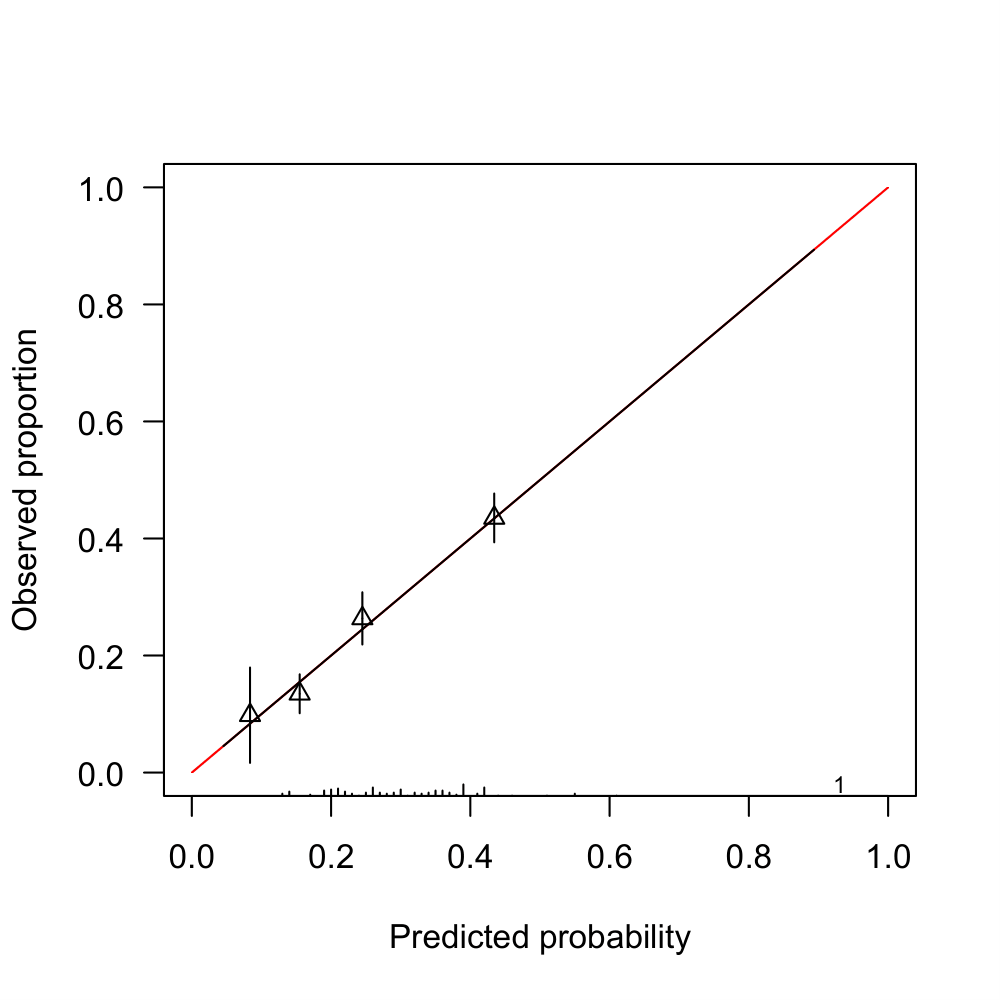 | 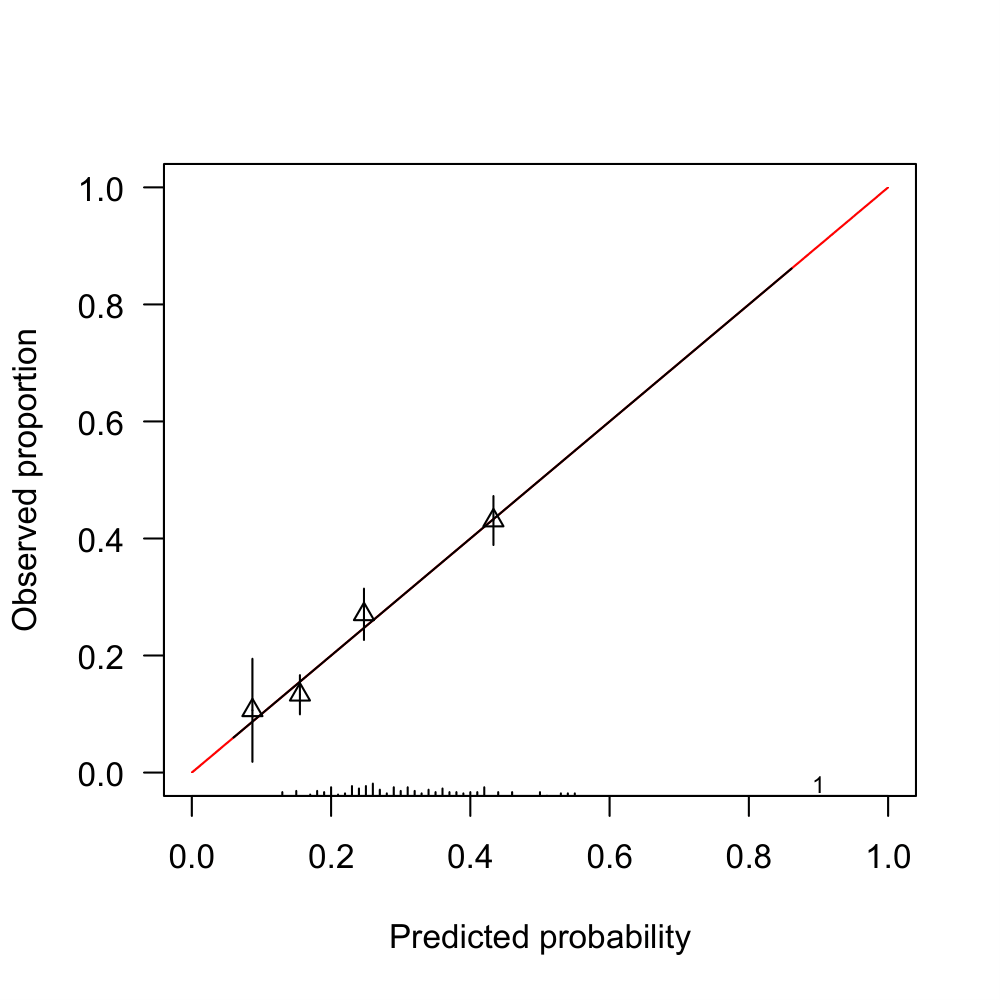 |
| 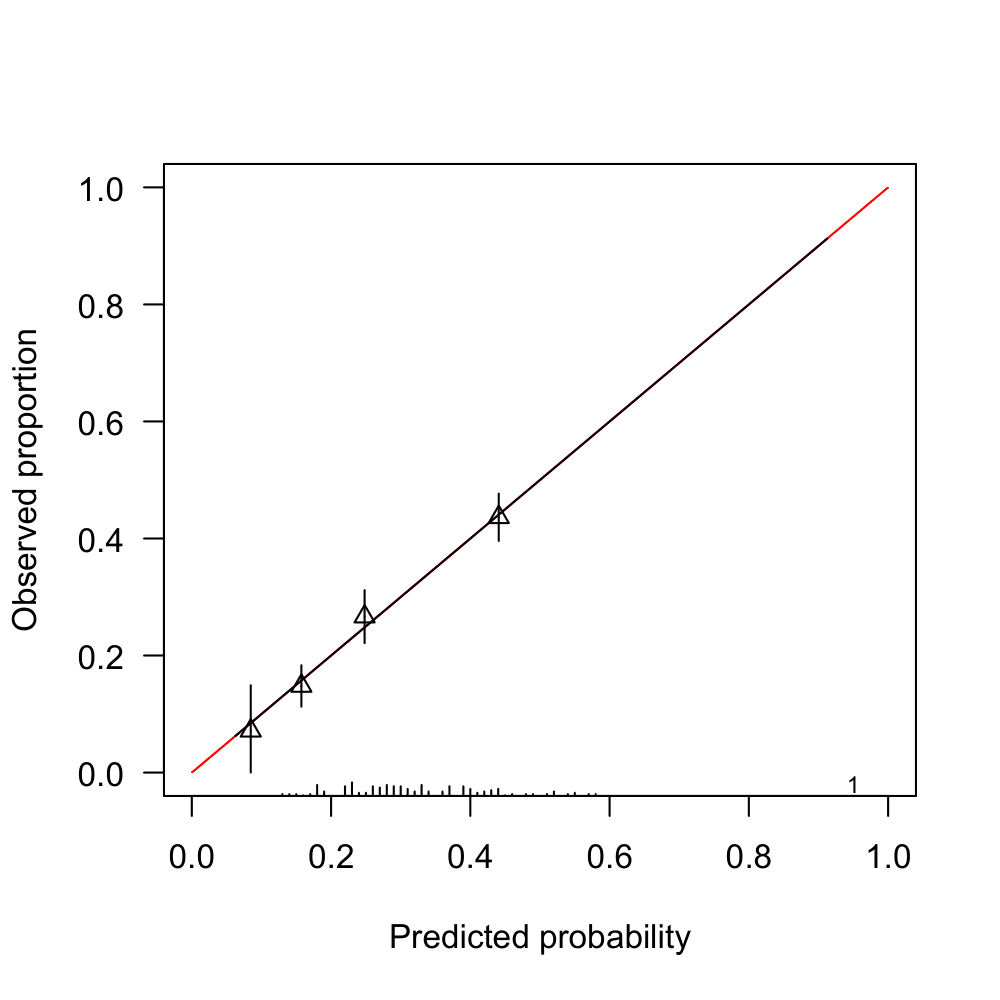 | 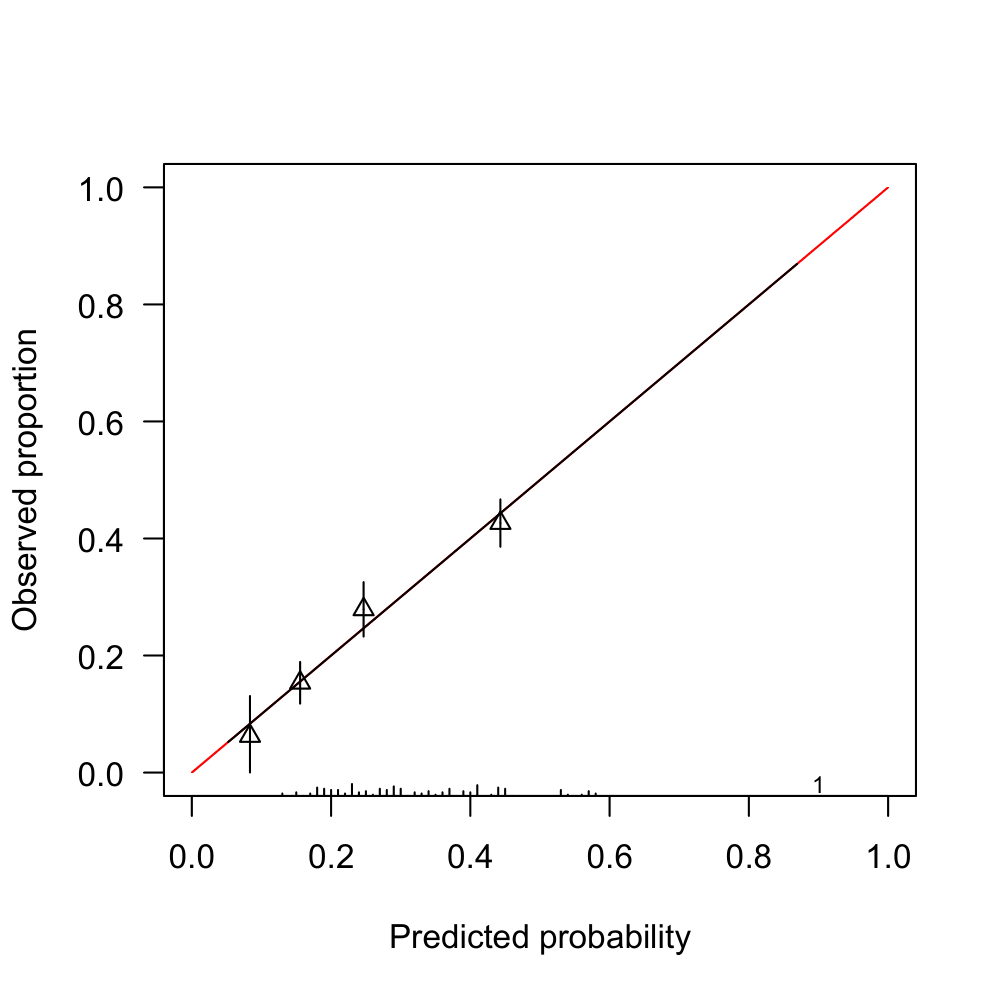 | 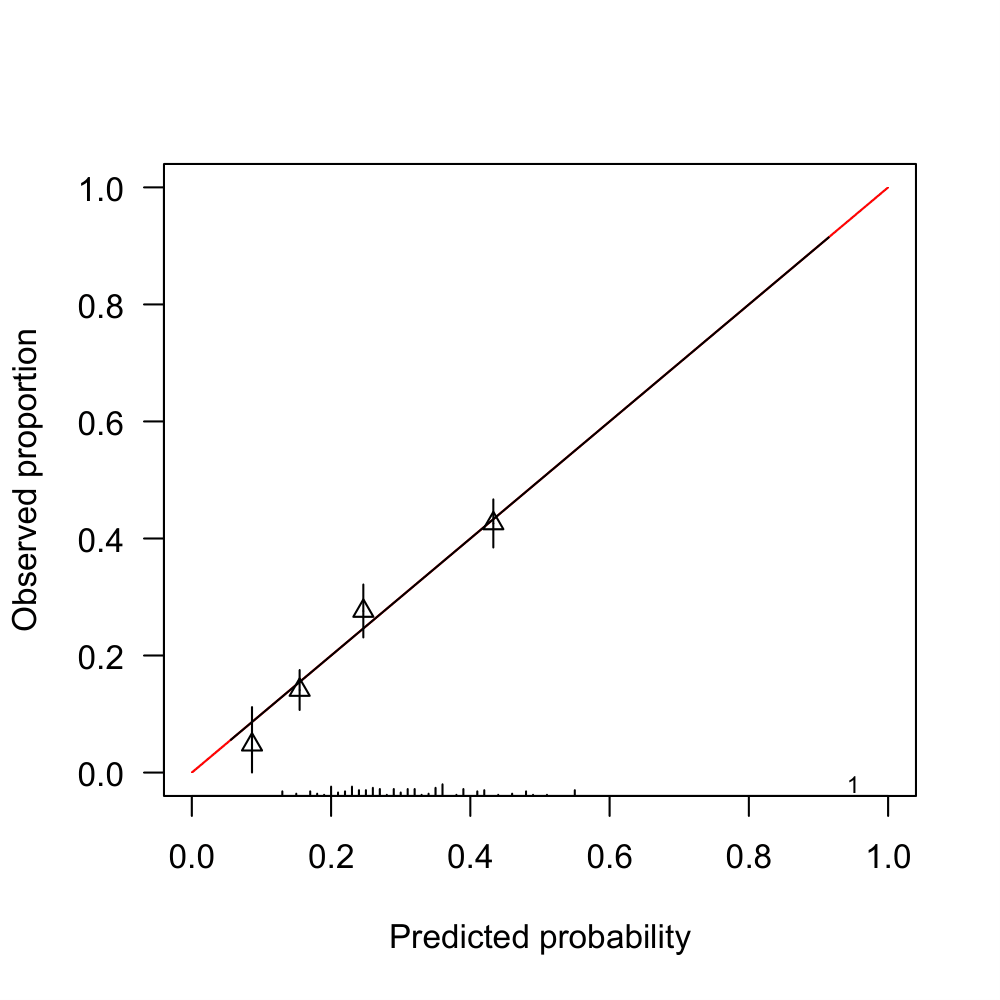 |

**After recalibration, partial model**

| 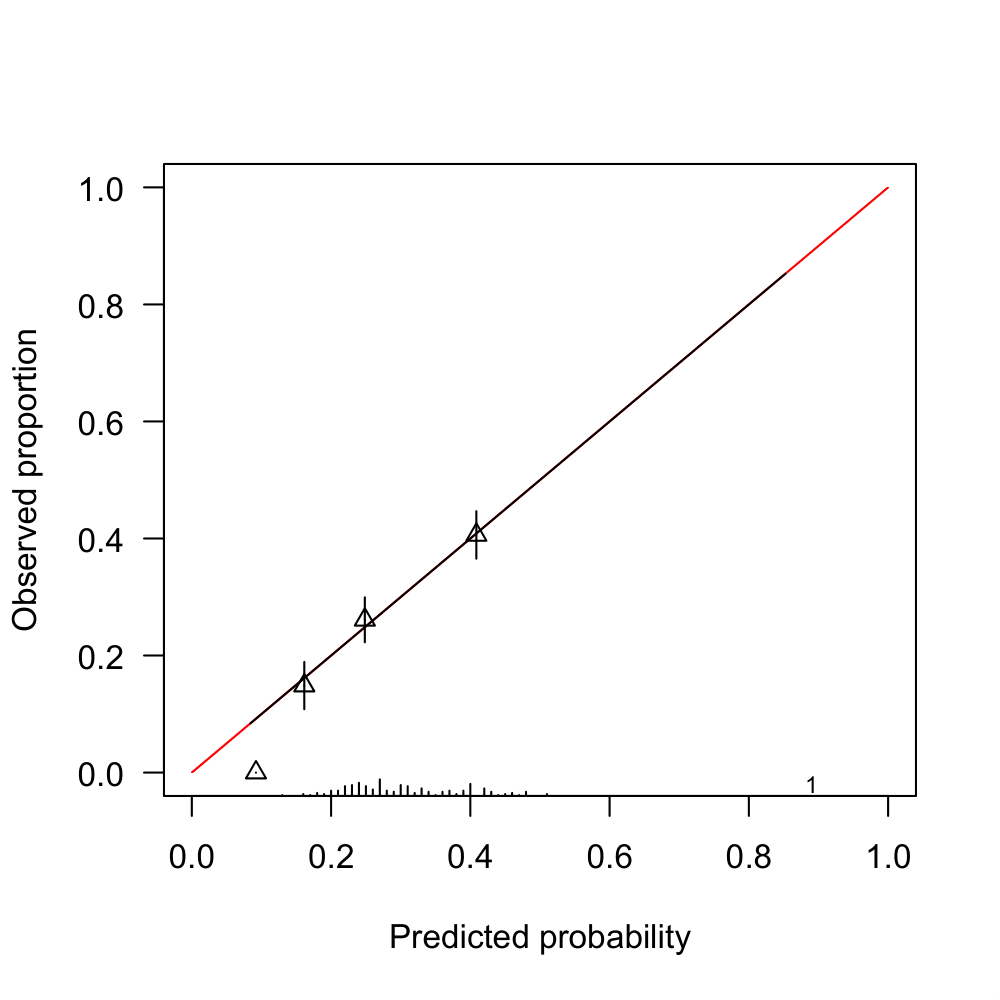 | 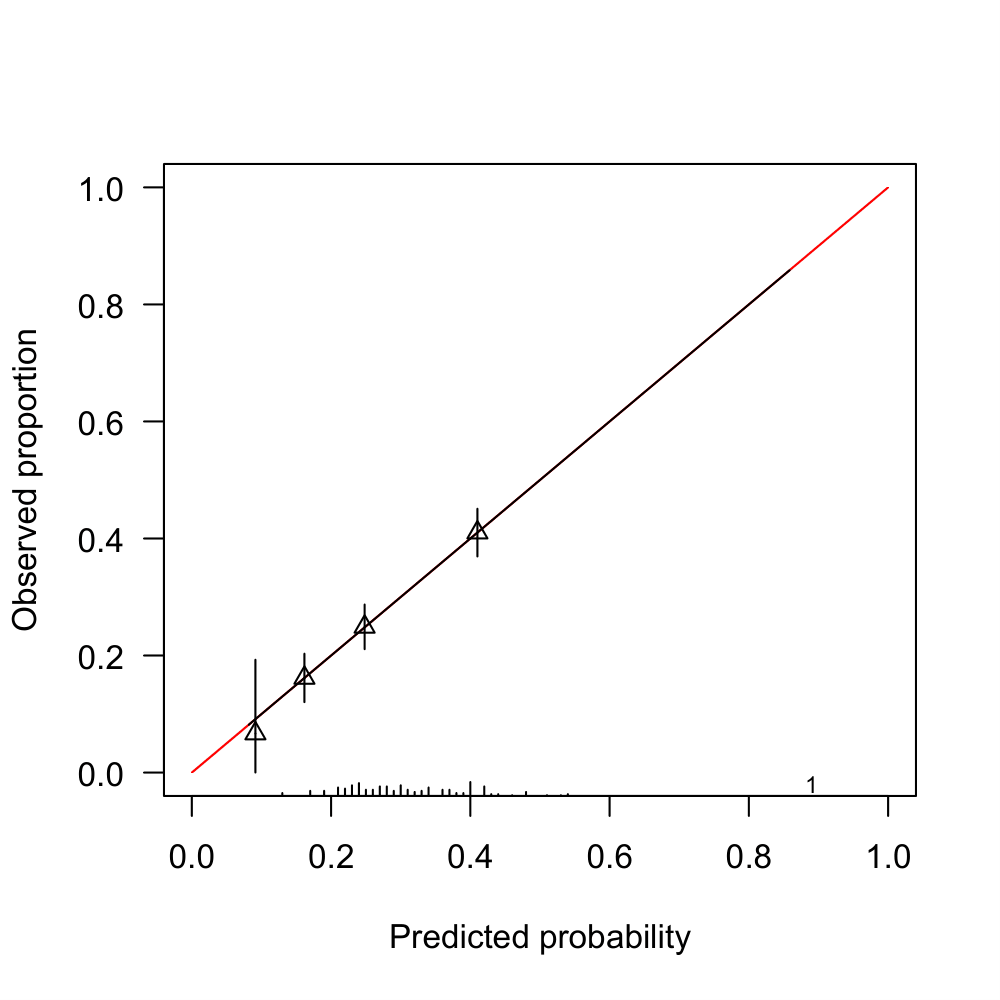 | 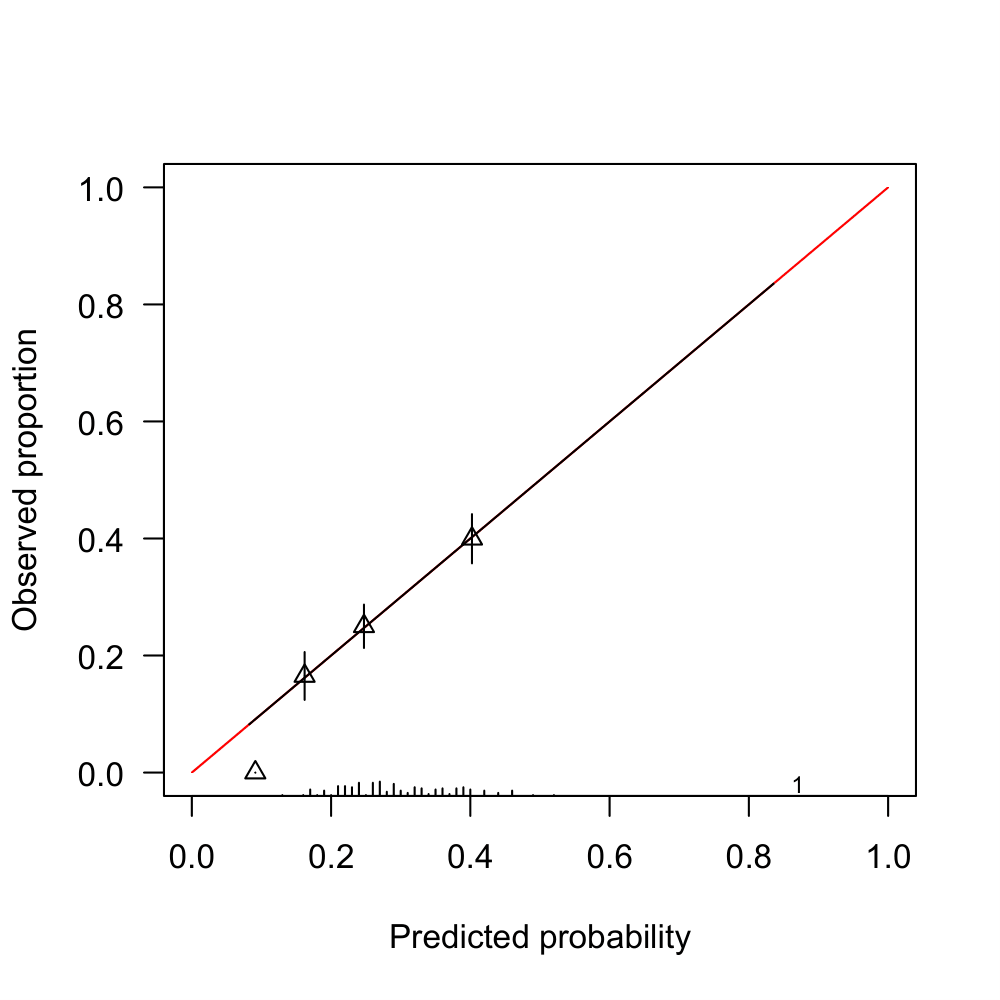 |
| --- | --- | --- |
| 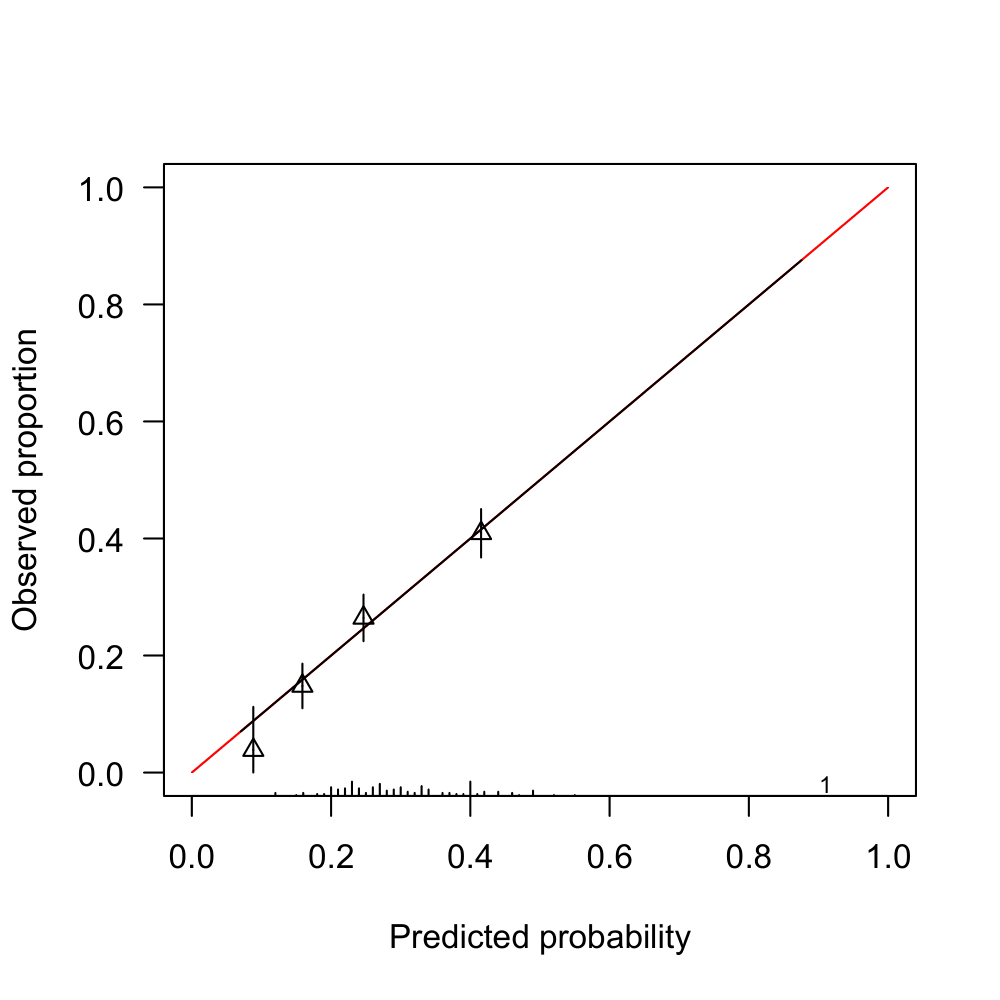 | 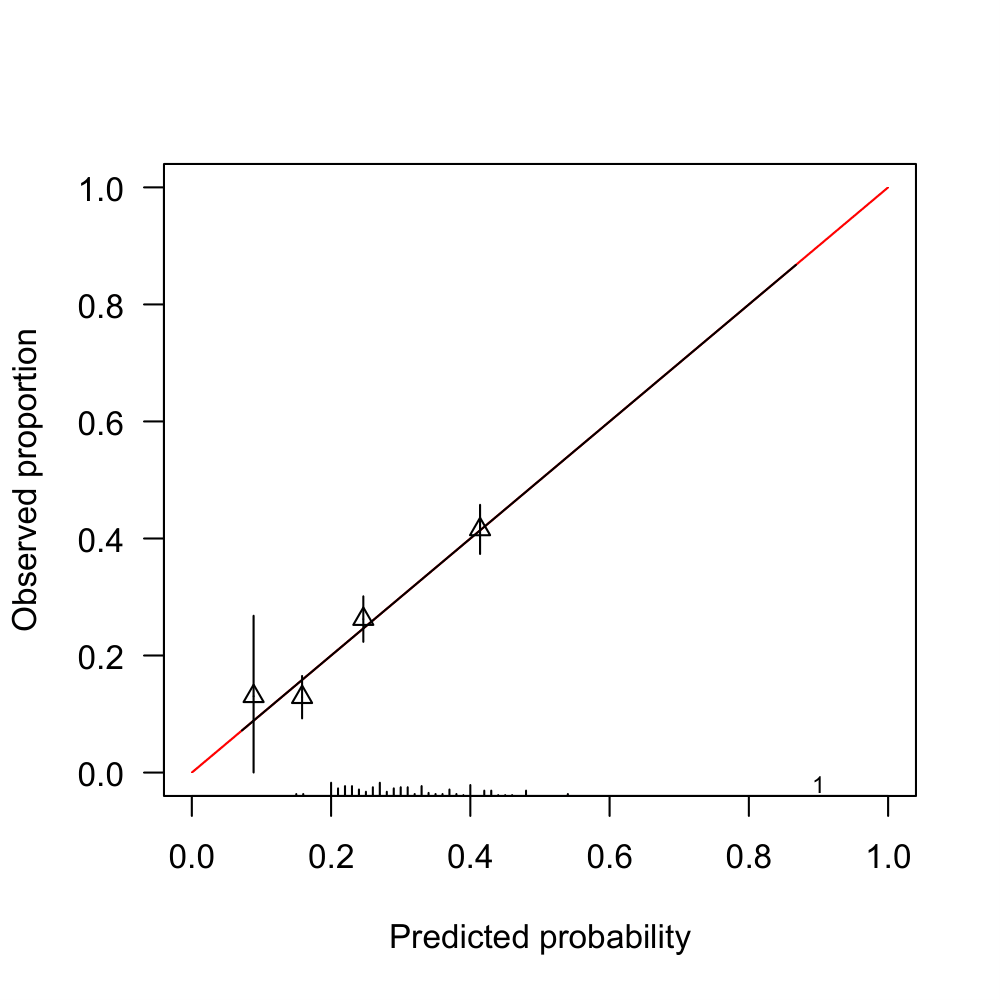 | 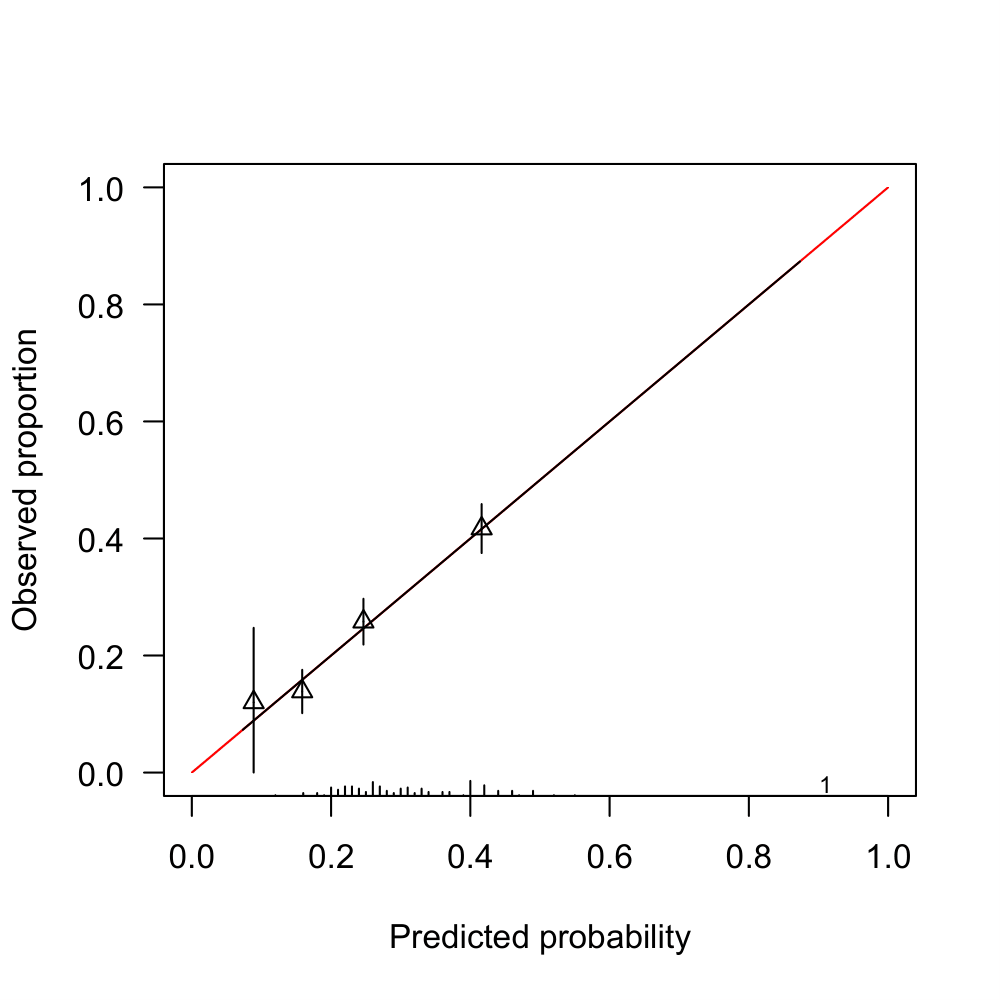 |
| 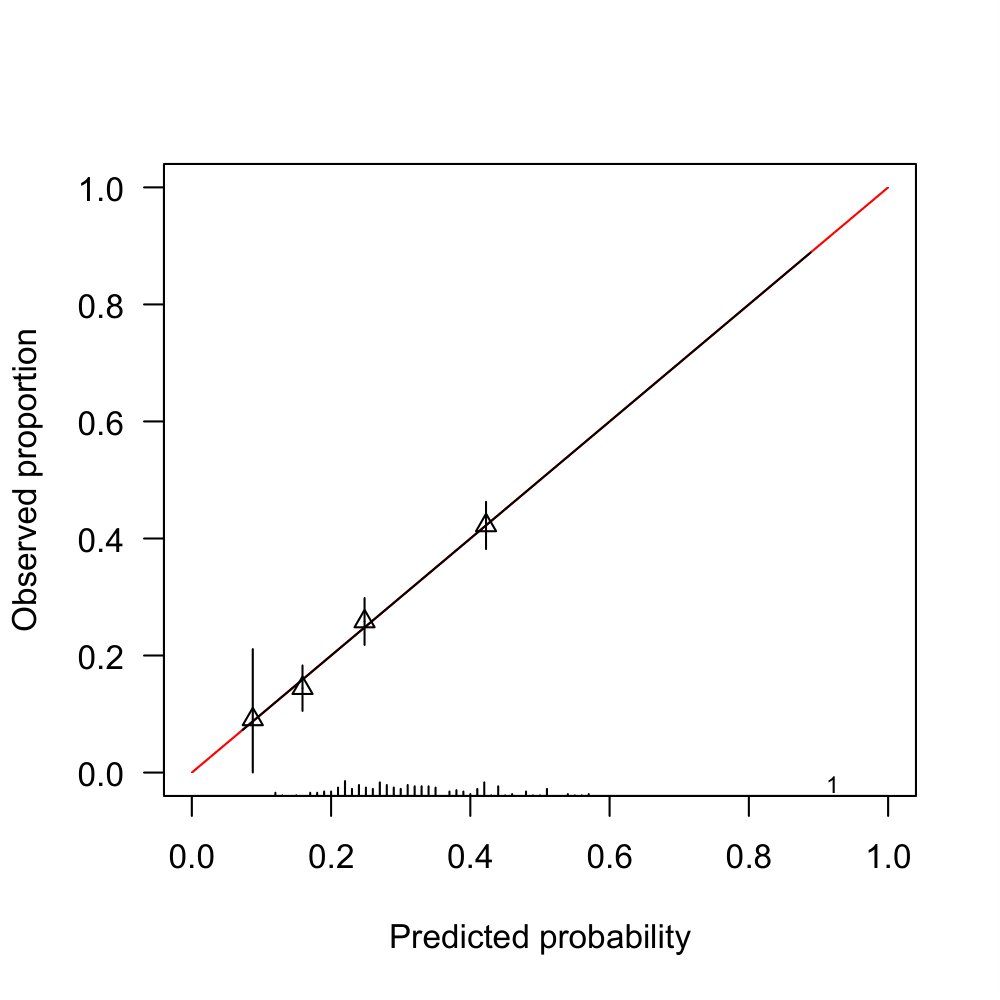 | 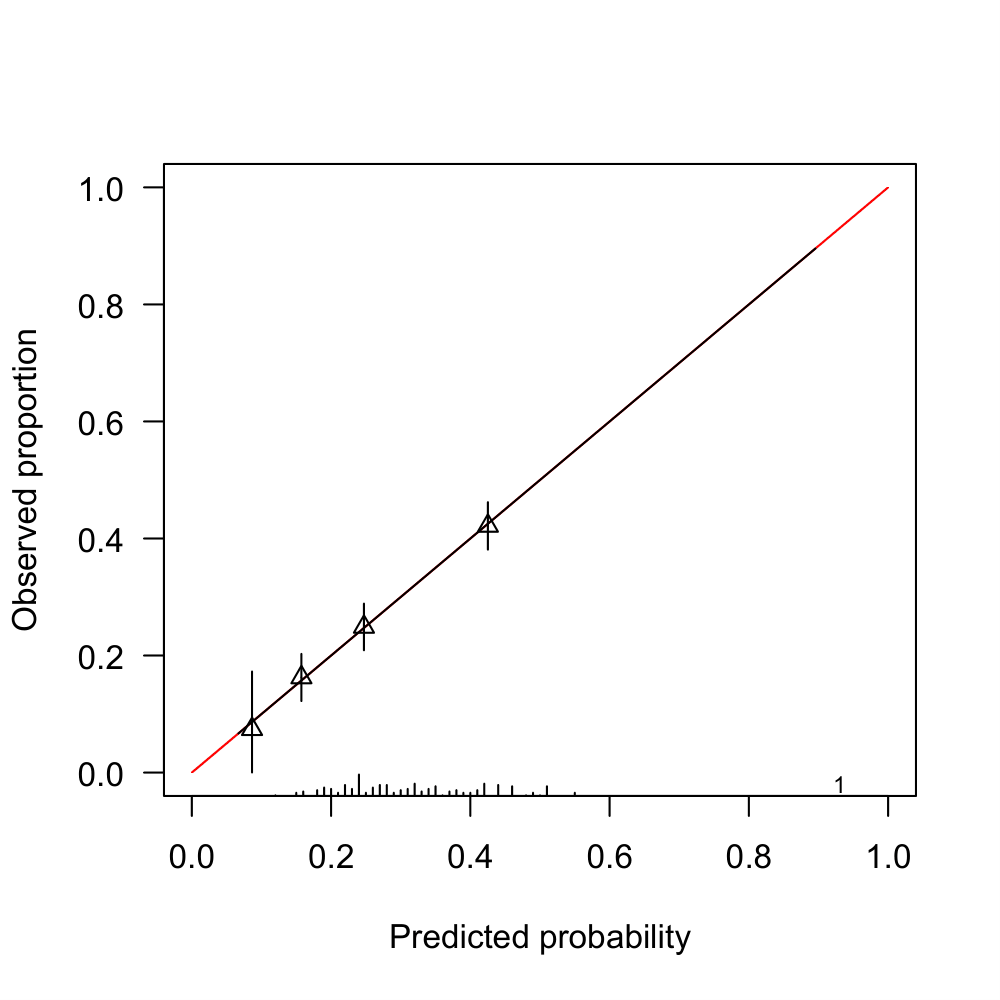 | 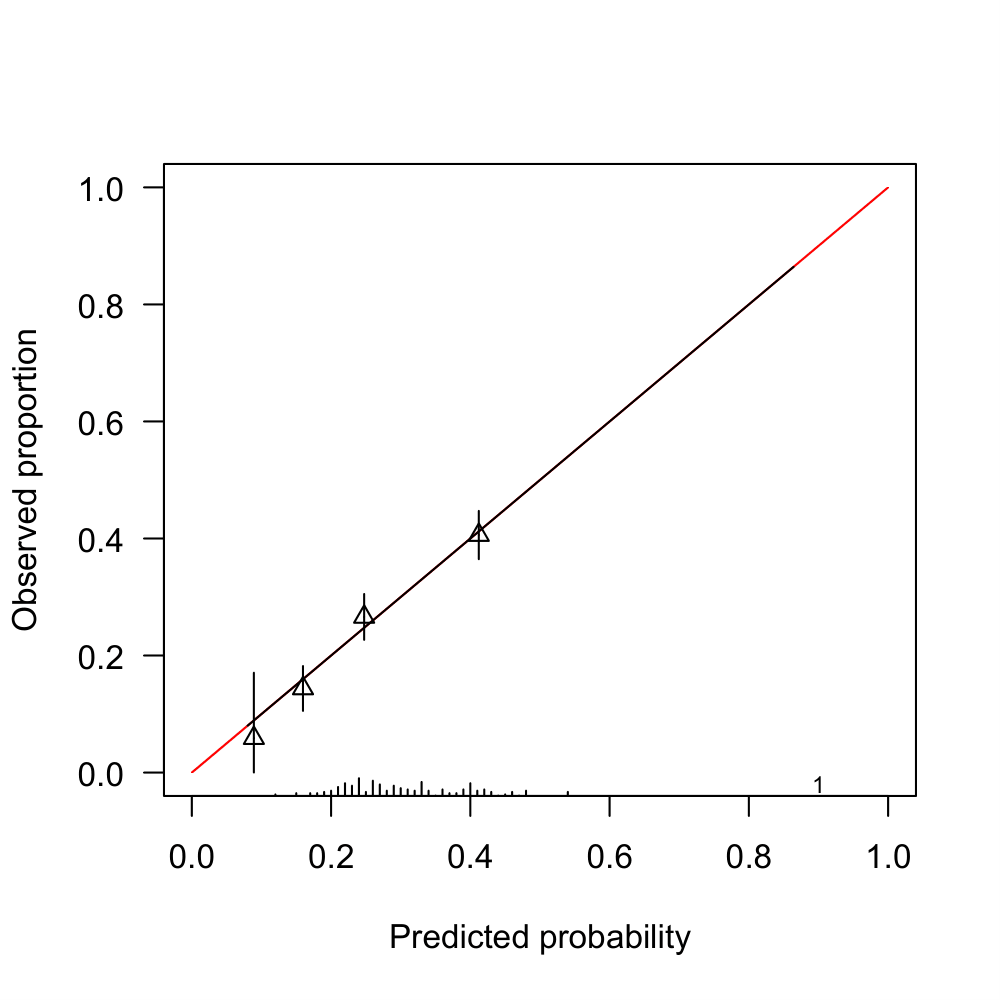 |
