# Supplementary material for: External validation and recalibration of the psychosis metabolic risk calculator (PsyMetRiC) in young adults with chronic psychotic disorders in the Netherlands
Source: Eur Psychiatry. 2026 Mar 9;69(1):e44. doi: 10.1192/j.eurpsy.2026.10179 (PMC13122530; doi:10.1192/j.eurpsy.2026.10179)
Supplement: Quadackers et al. supplementary material [file S0924933826101795sup001.zip › Supplementary Figure 5.docx]

**Supplementary Figure 5** Decision curve analysis plots for both PsyMetRiC-models (in UK- and NL-version) for the other nine imputed datasets

**DCA-plots for the full PsyMetRiC-models**

| **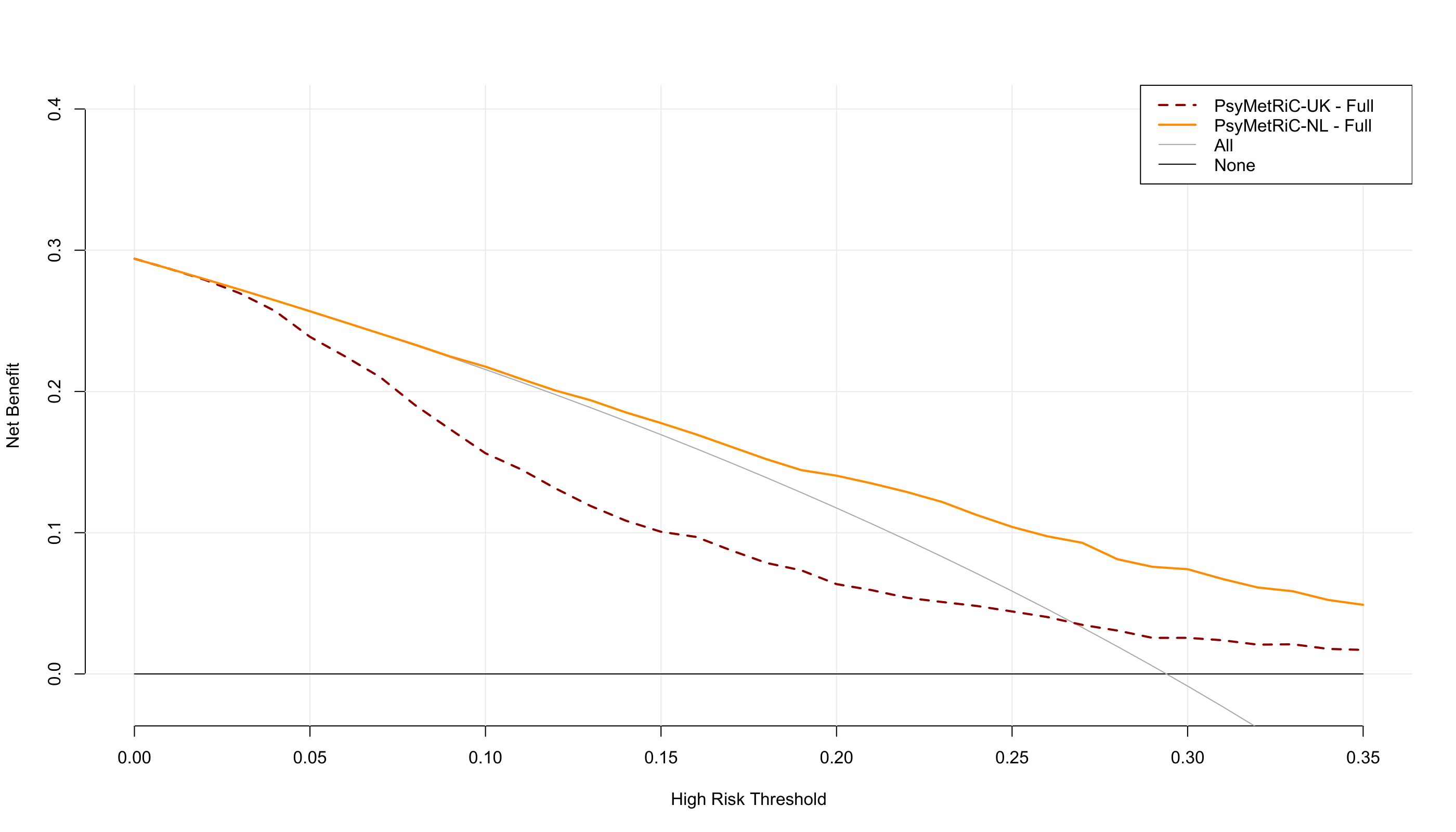** | **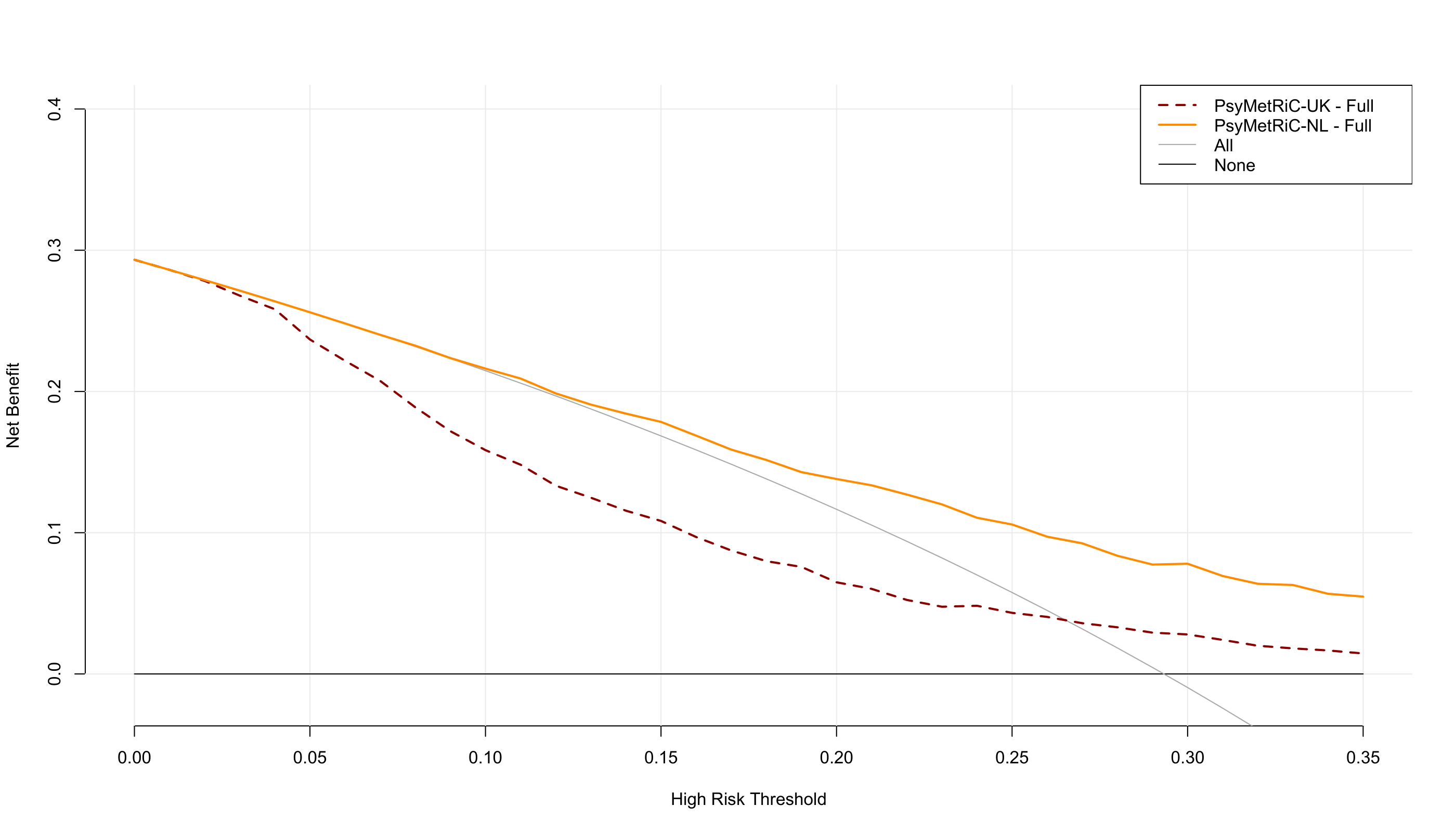** | **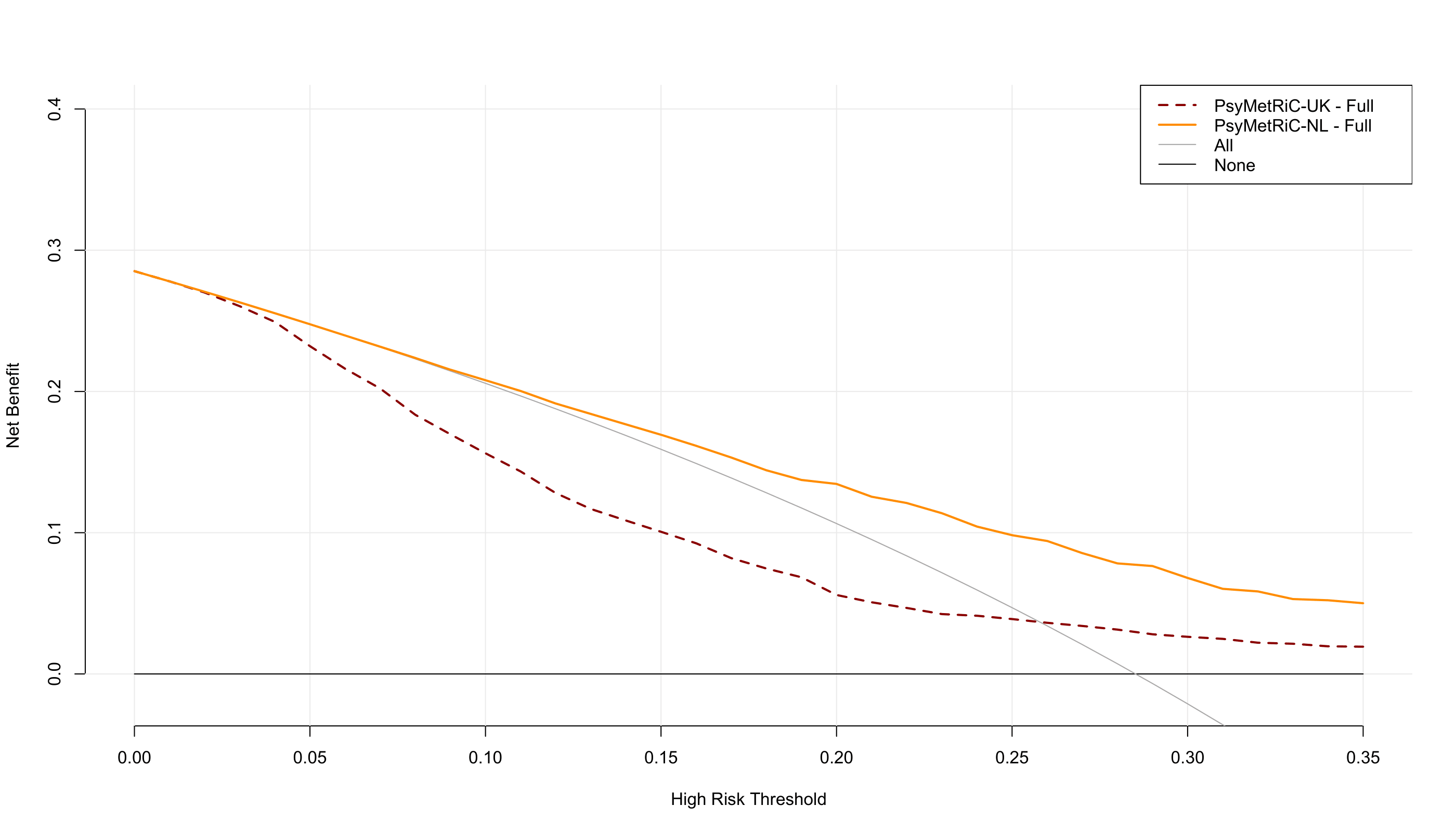** |
| --- | --- | --- |
| **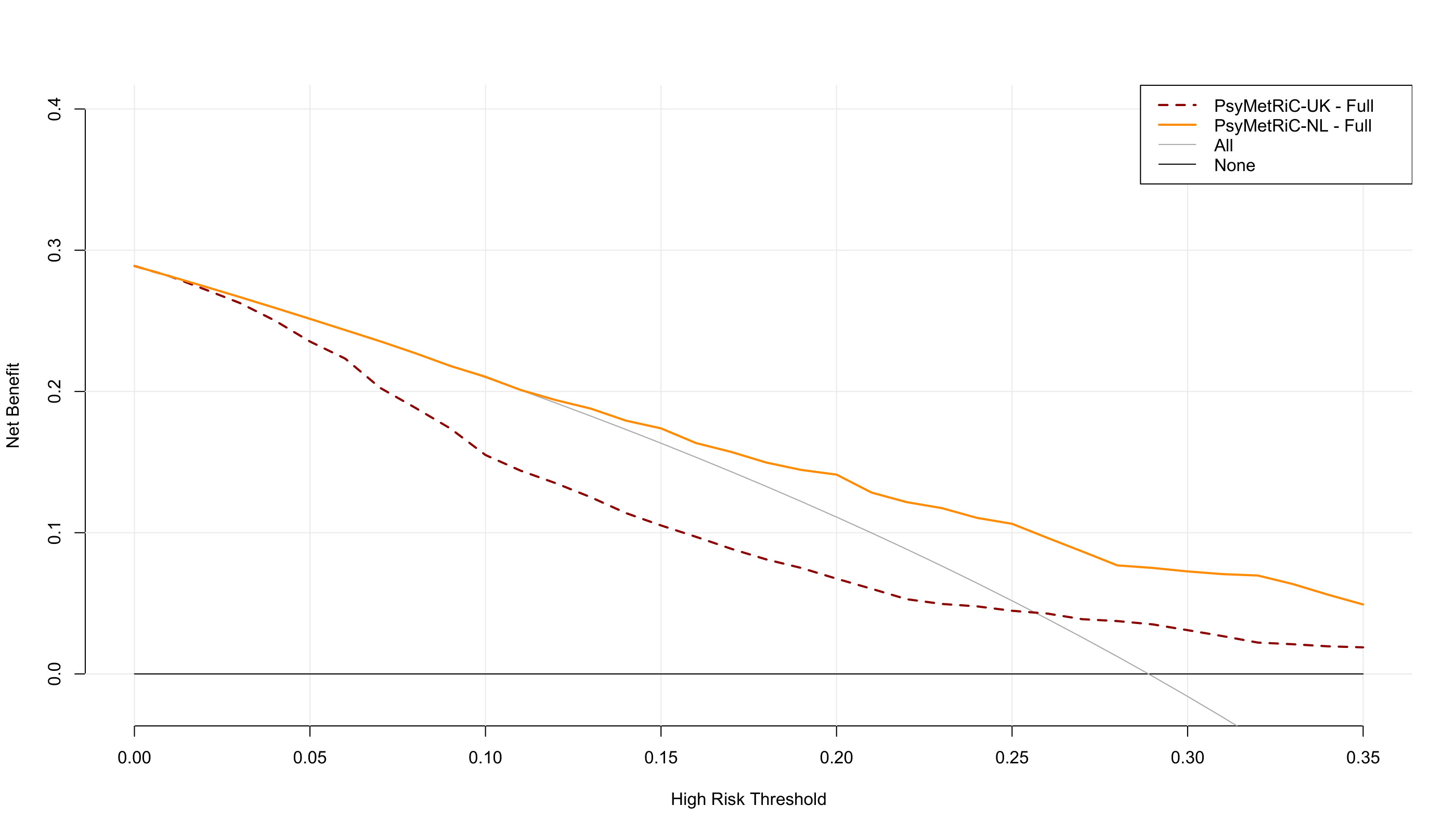** | **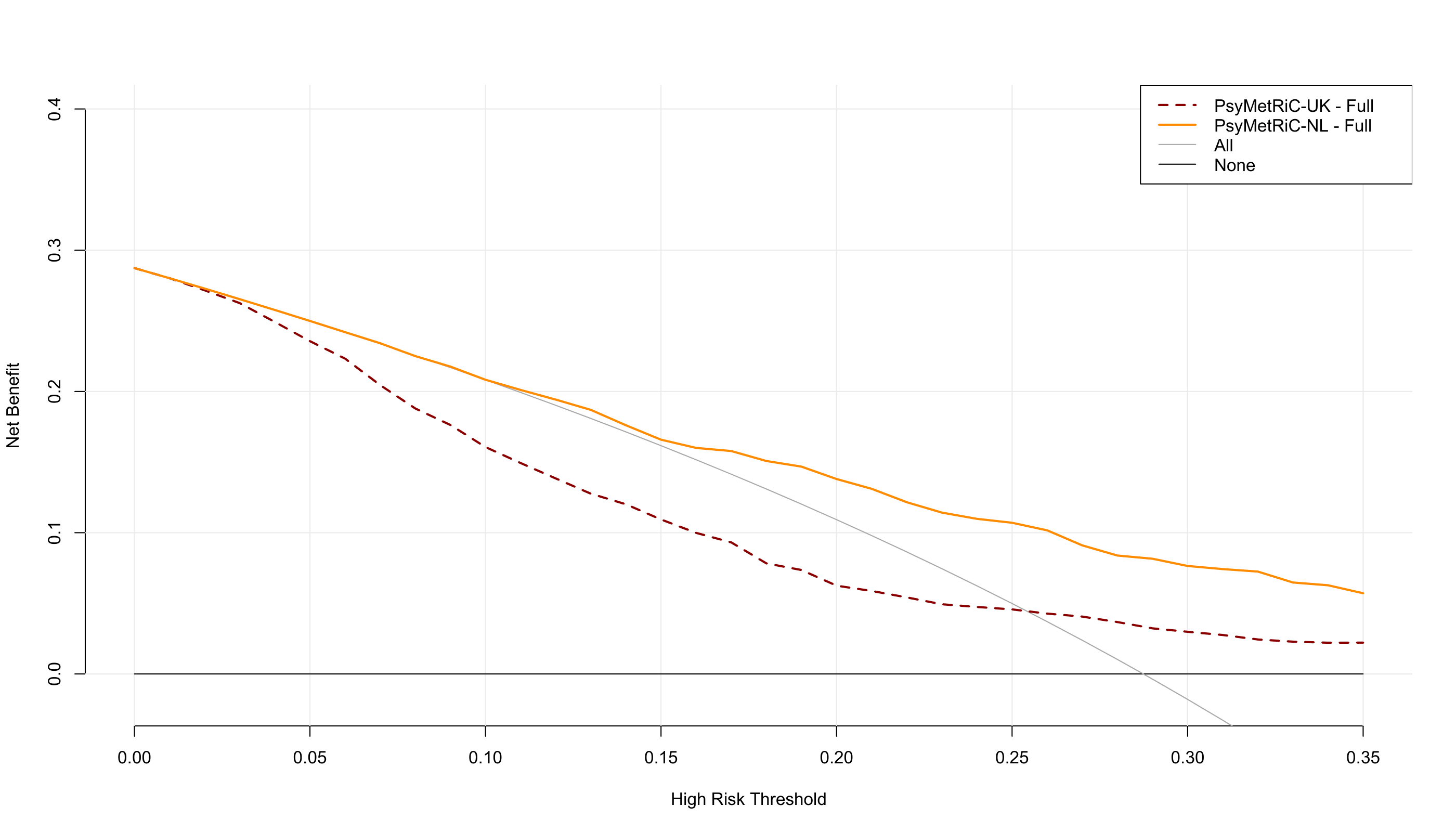** | **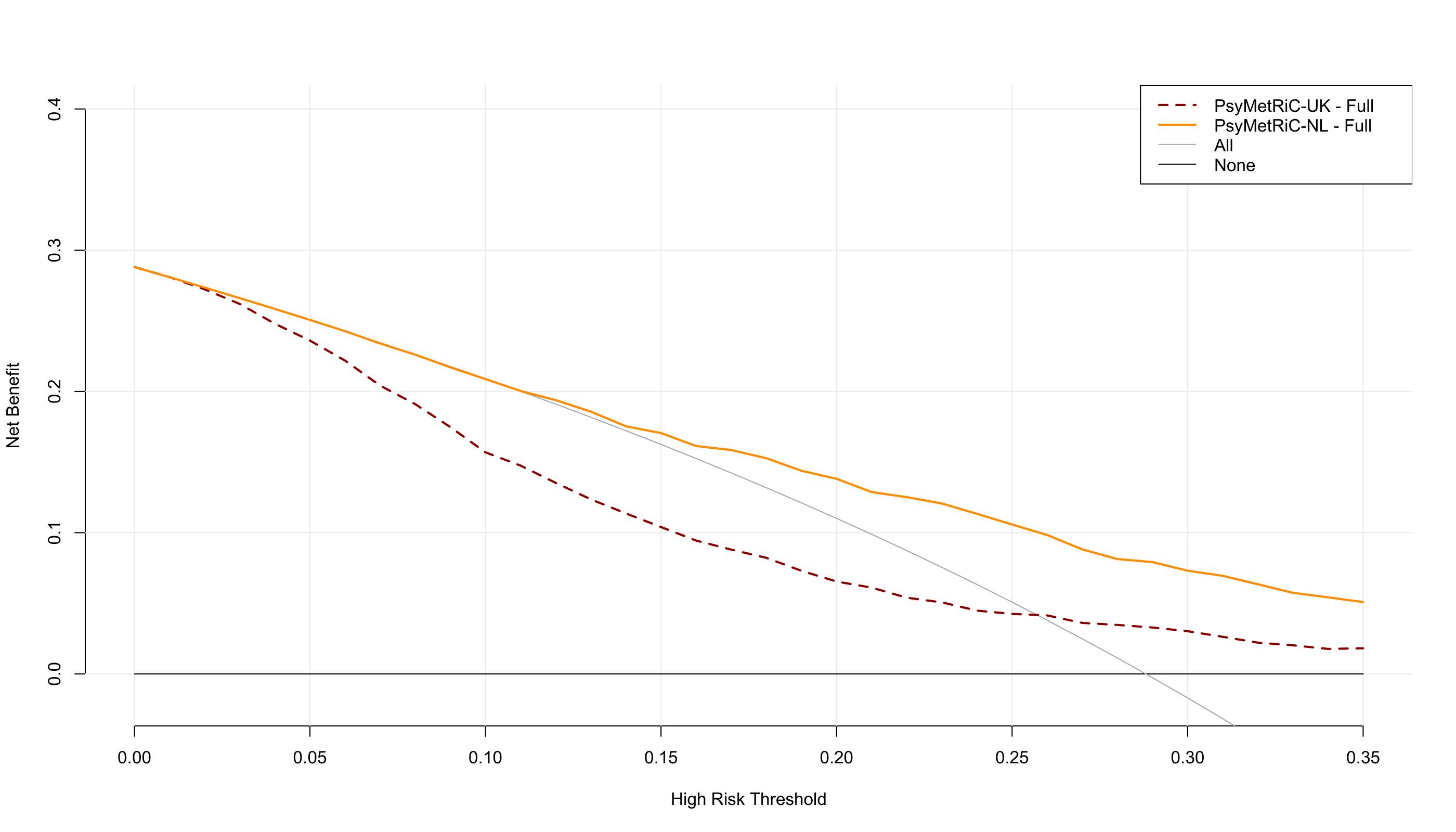** |
| **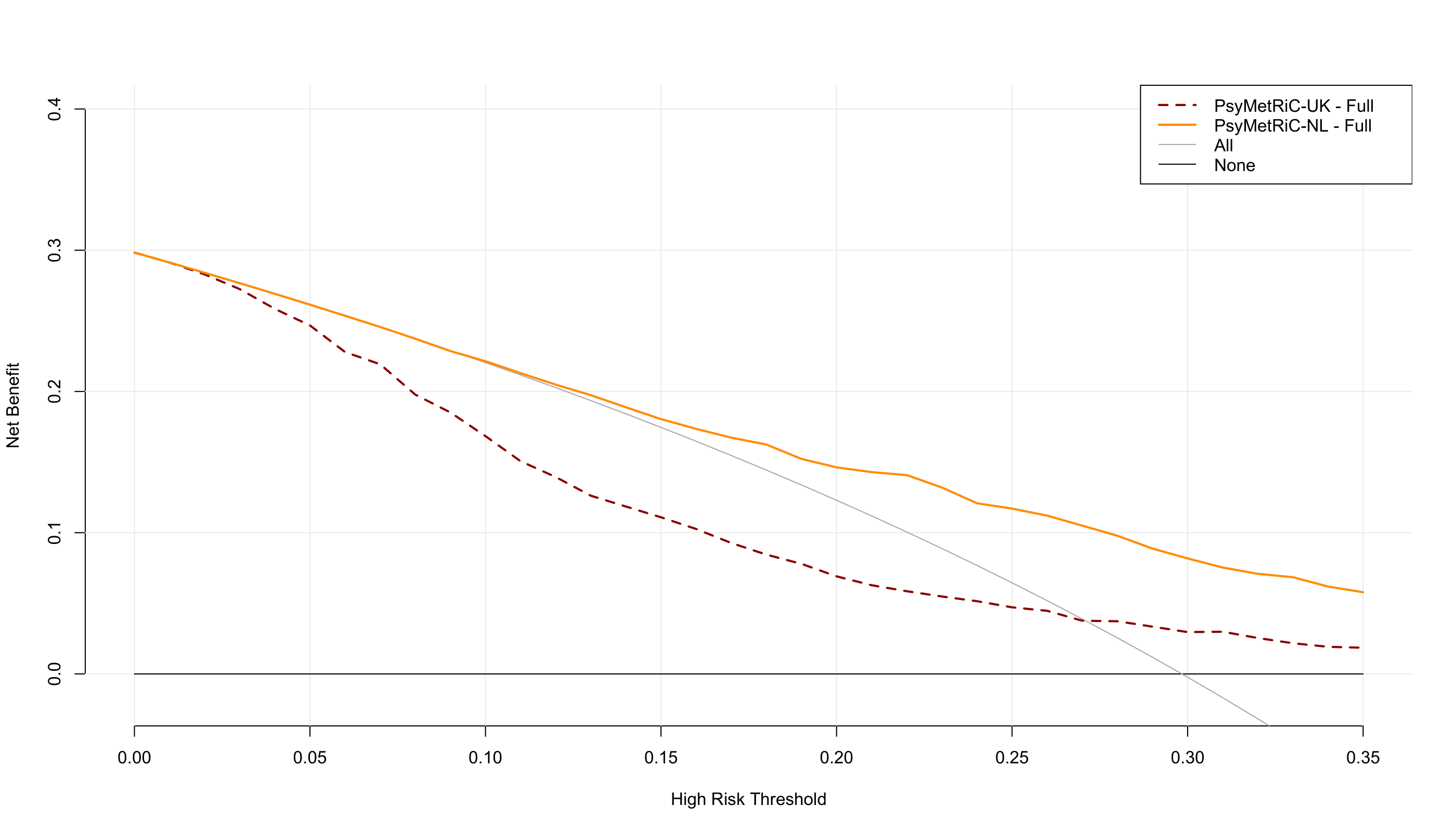** | **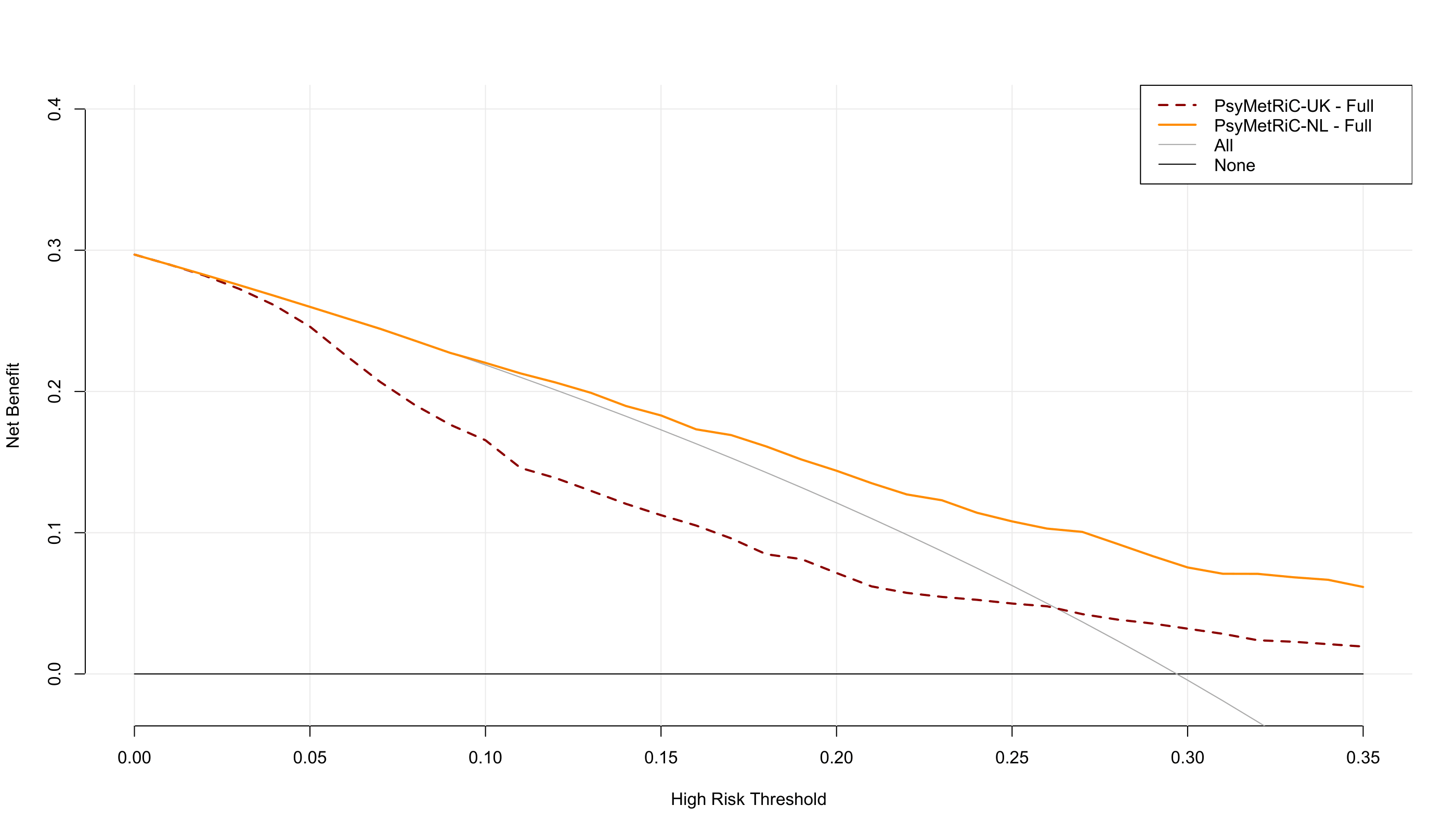** | **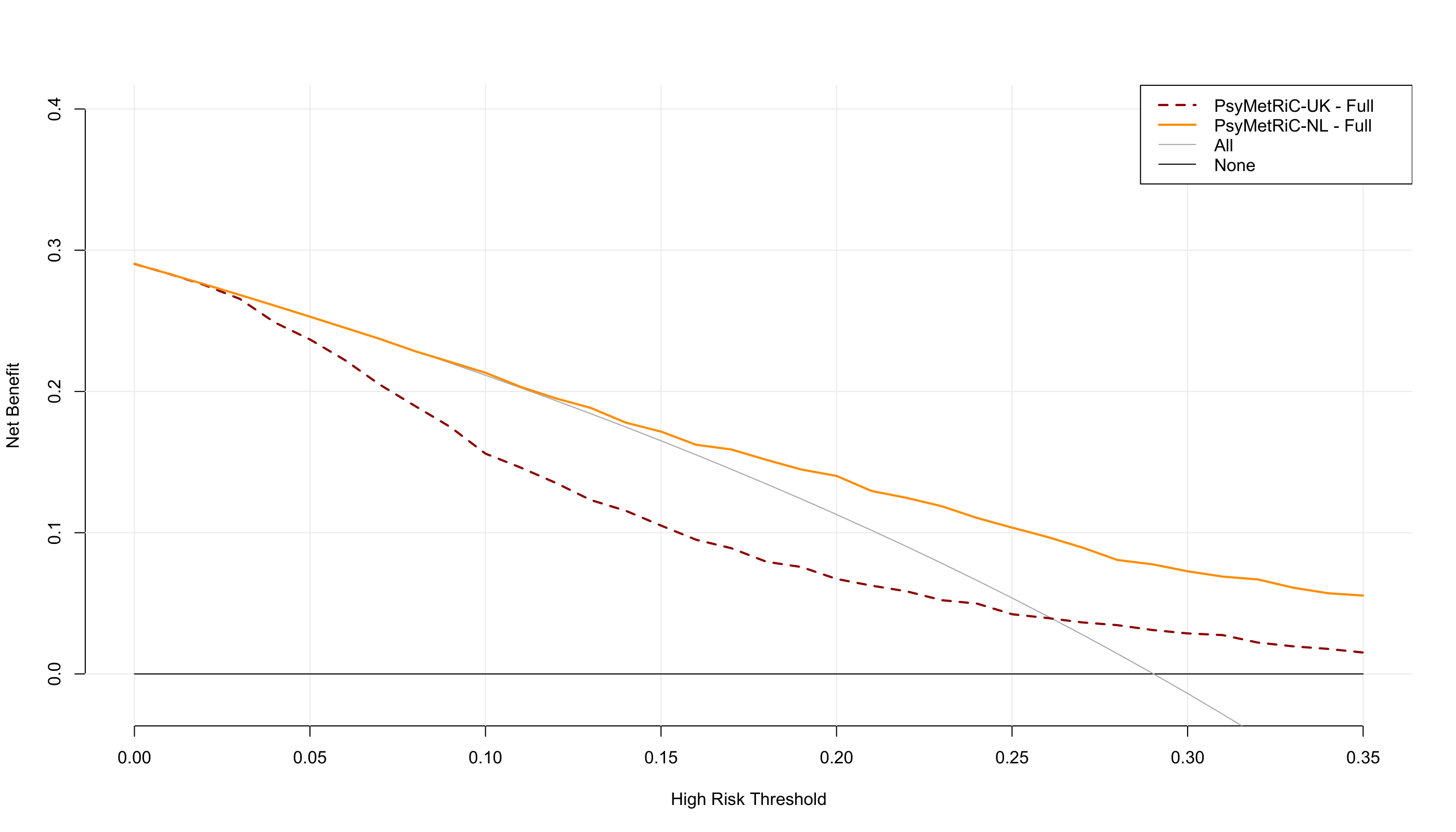** |

**DCA-plots for the partial PsyMetRiC-models**

| **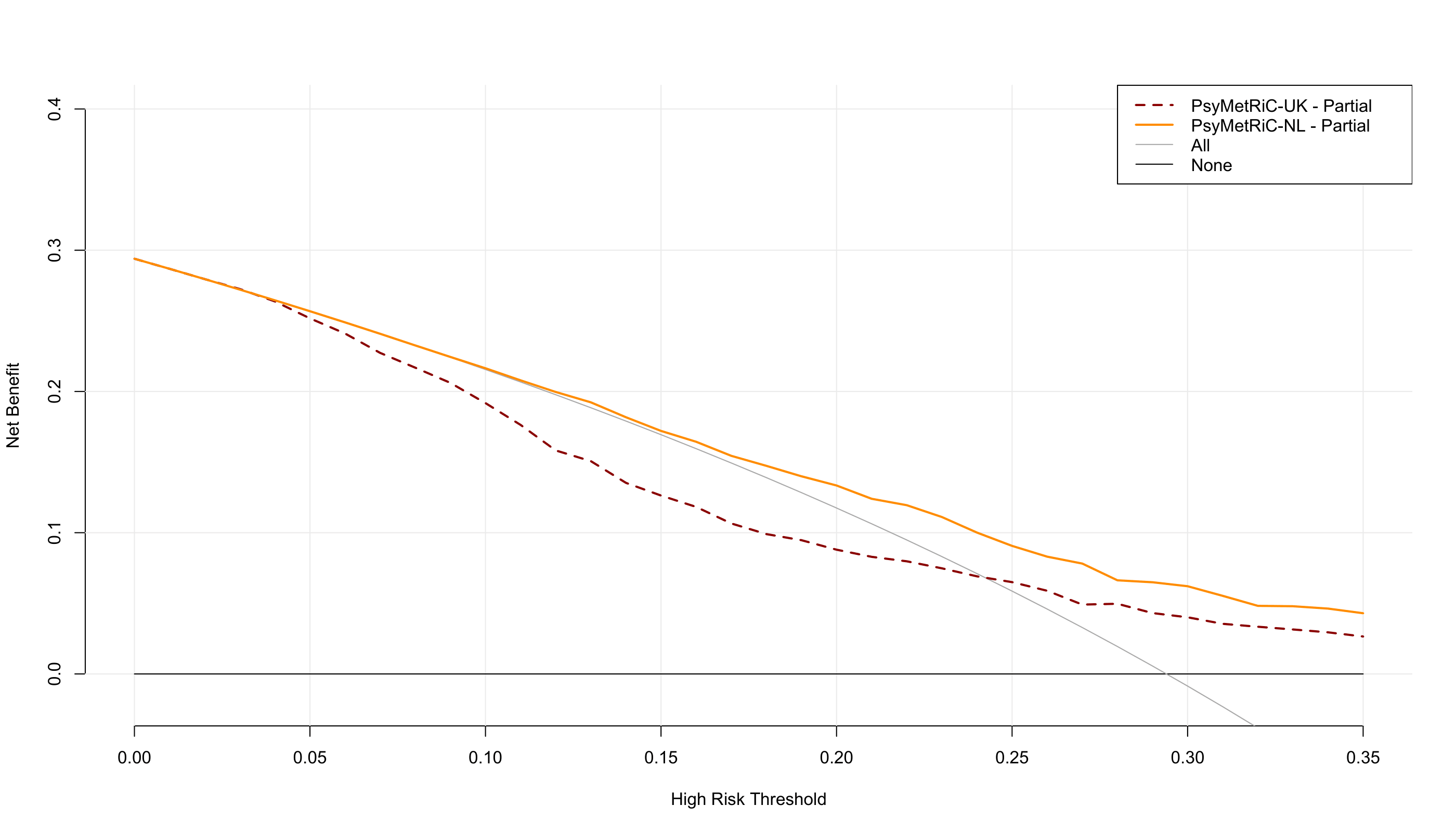** | **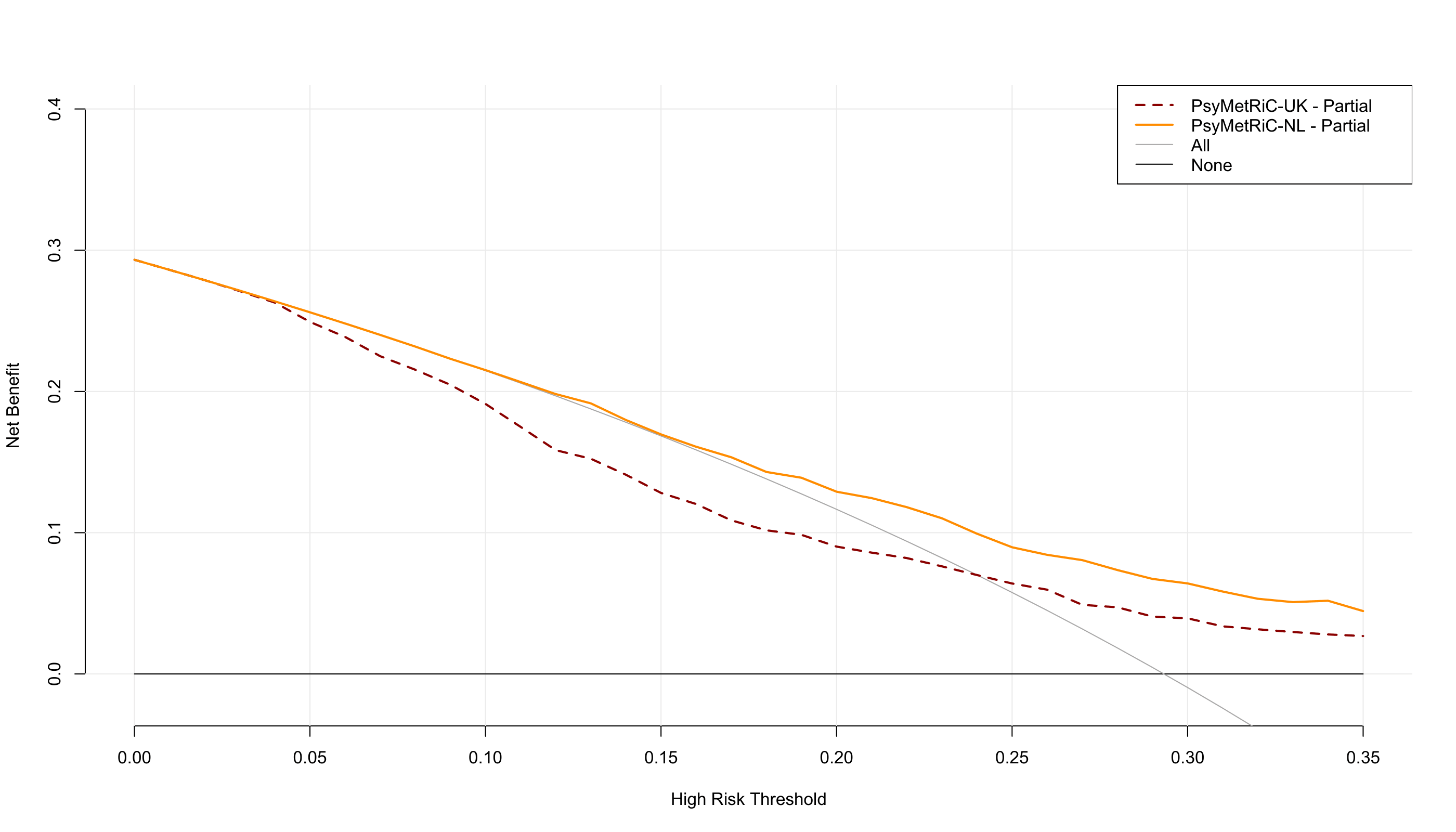** | **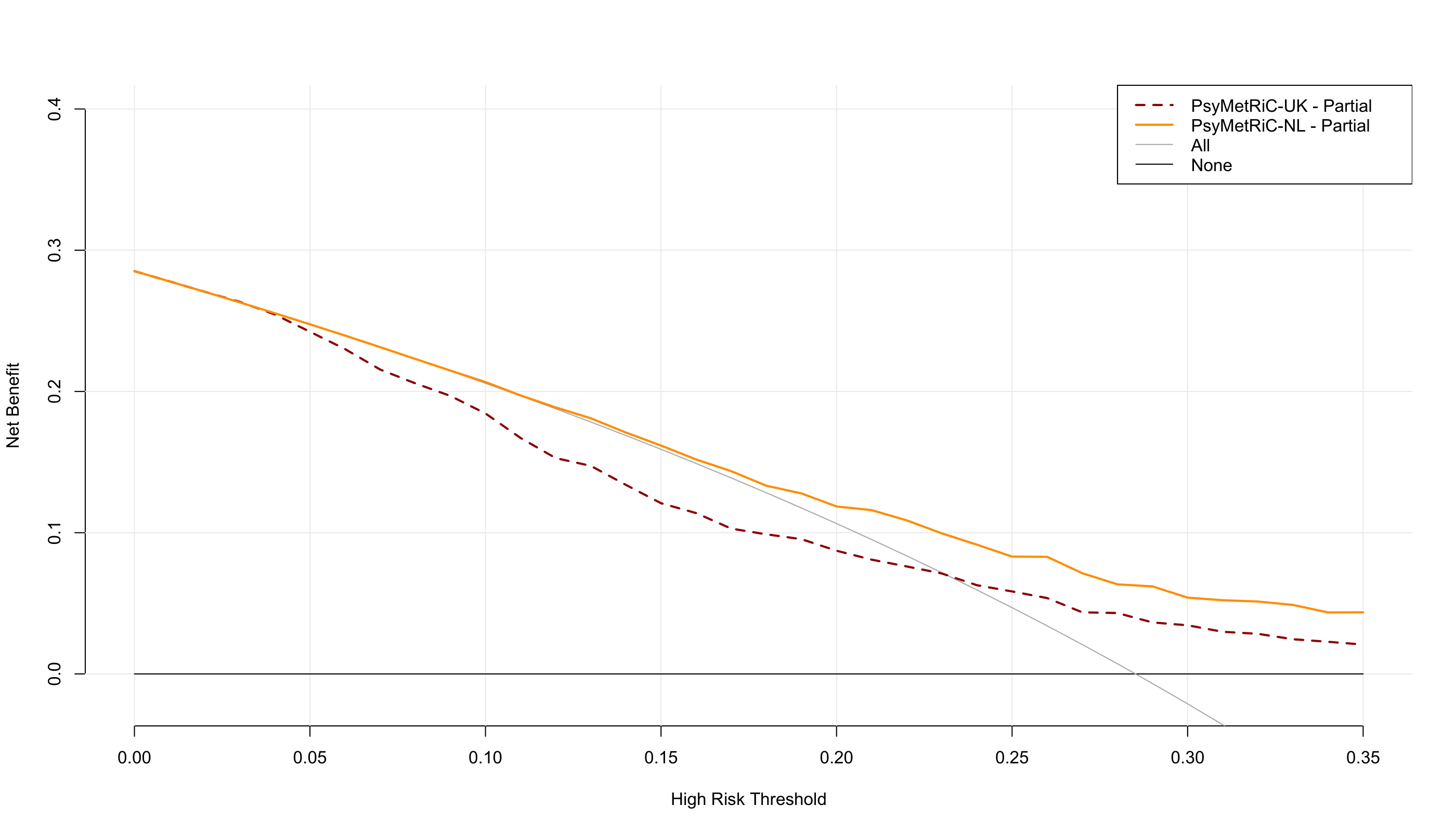** |
| --- | --- | --- |
| **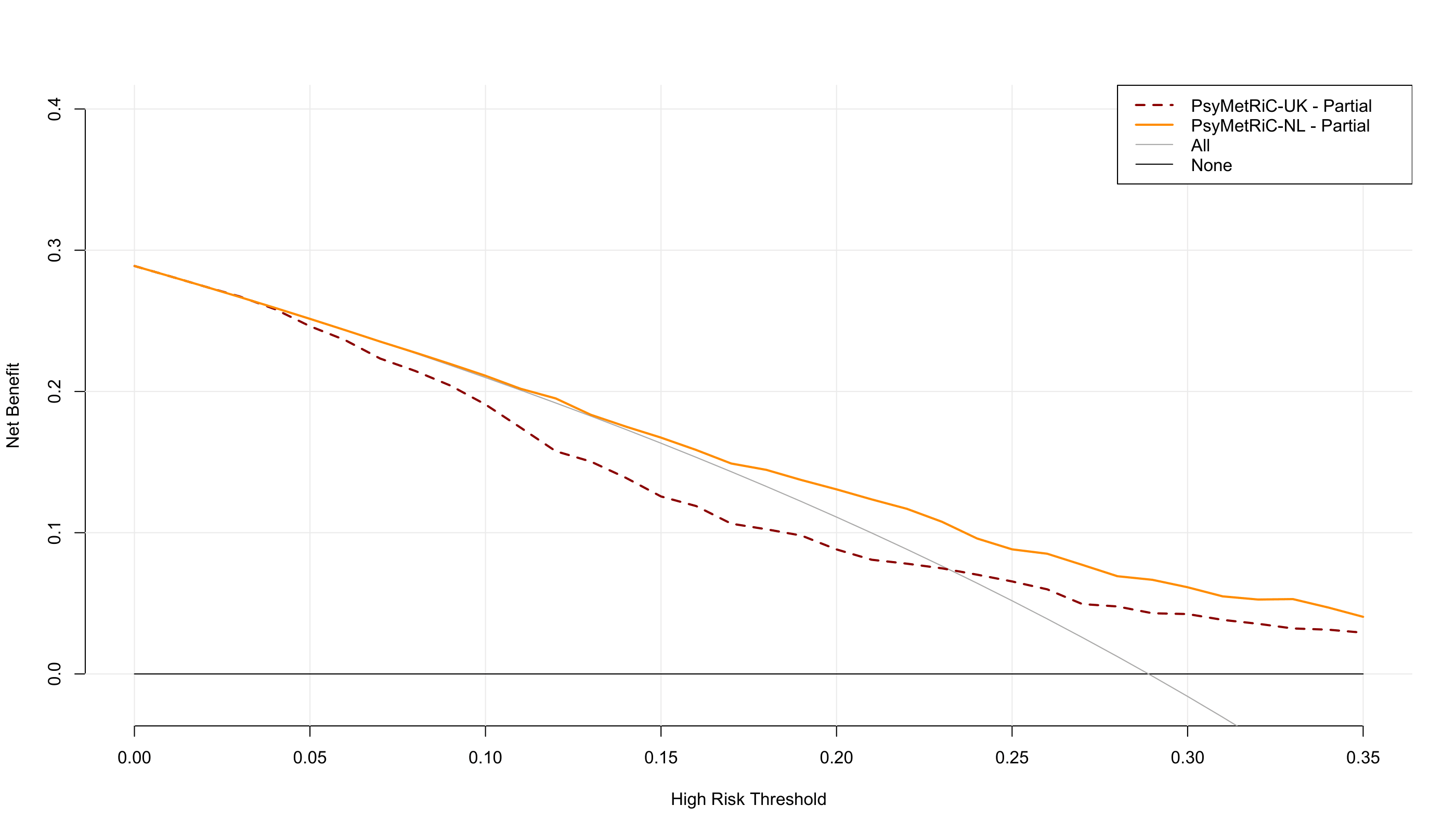** | **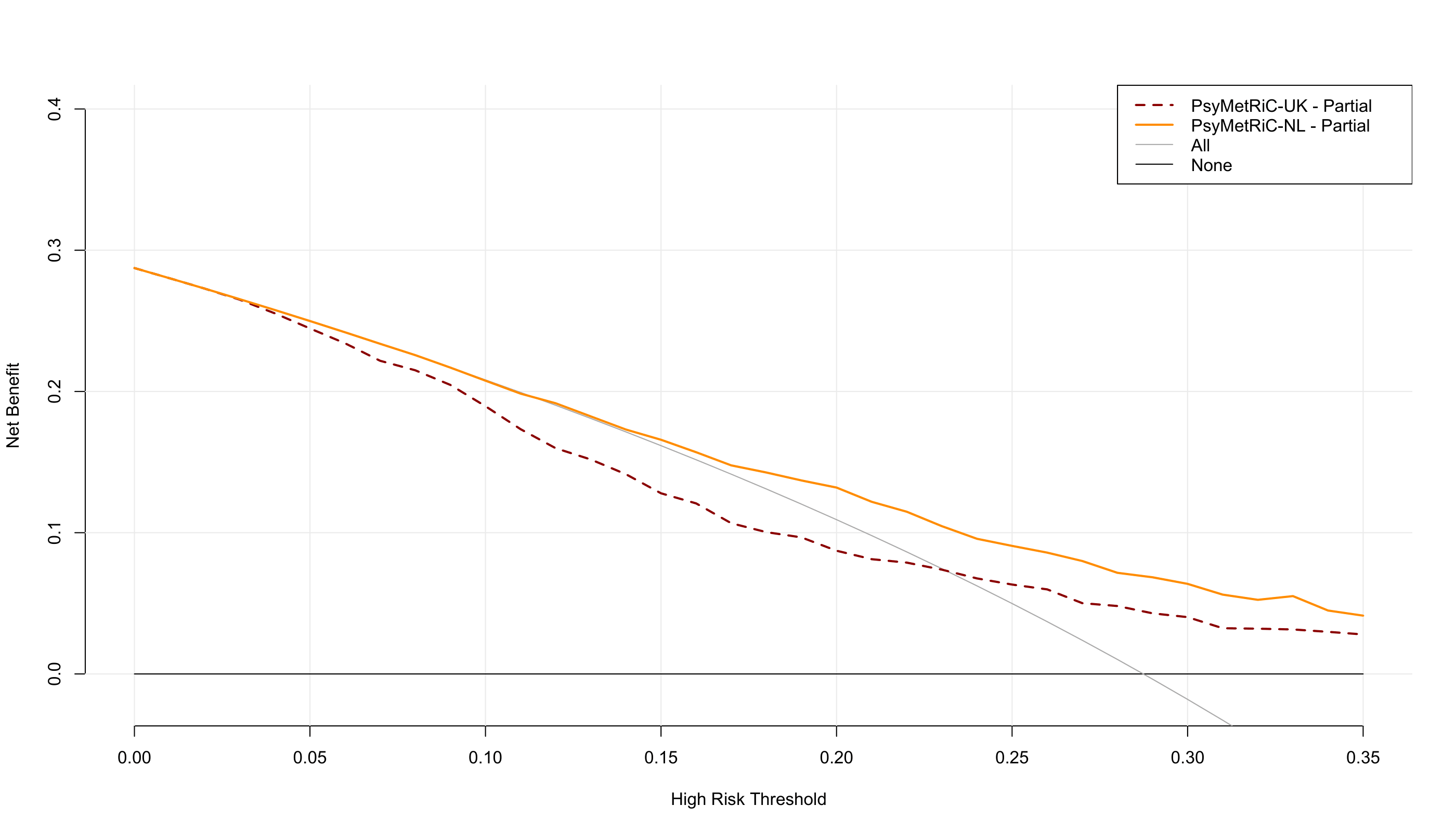** | **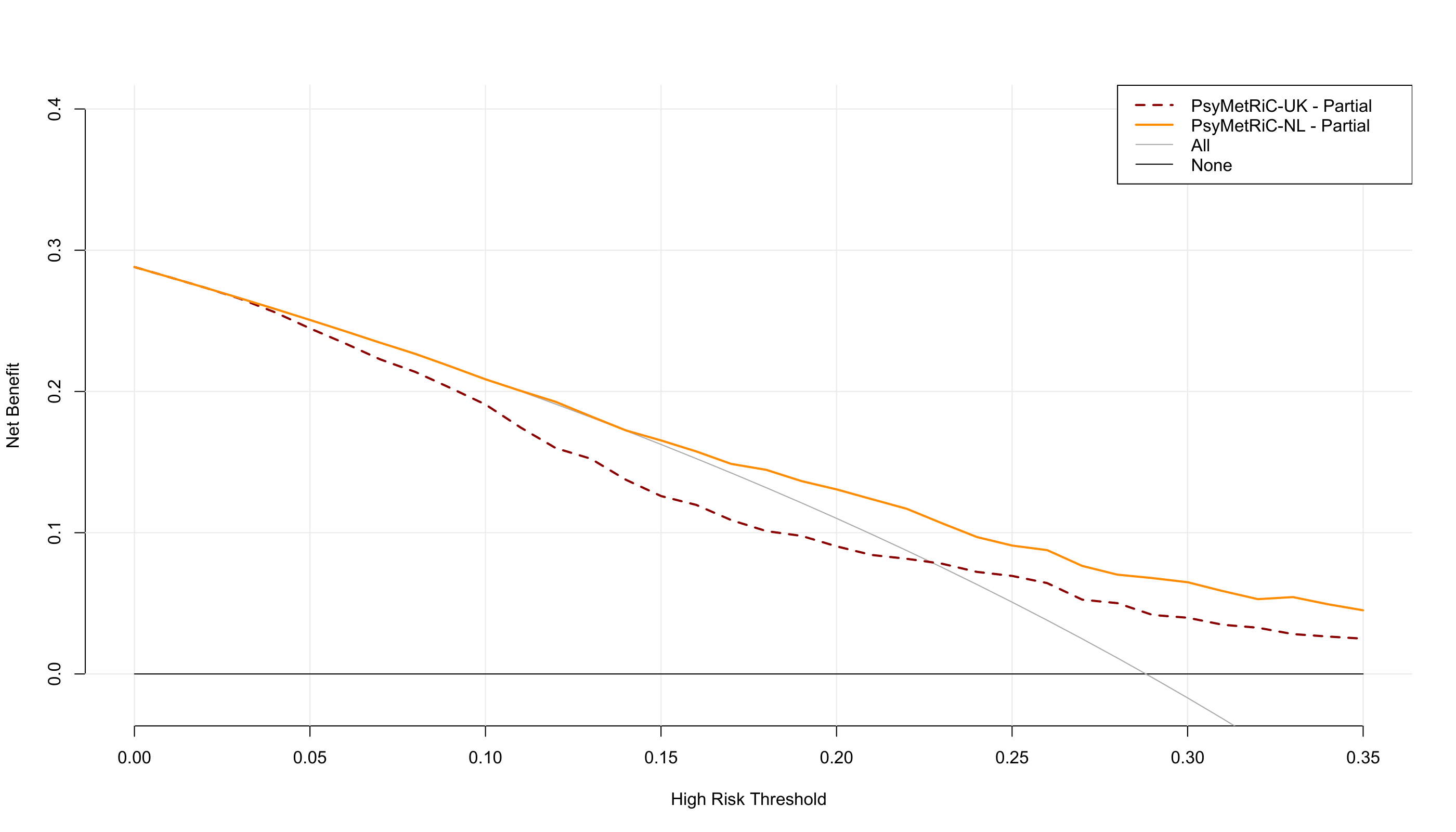** |
| **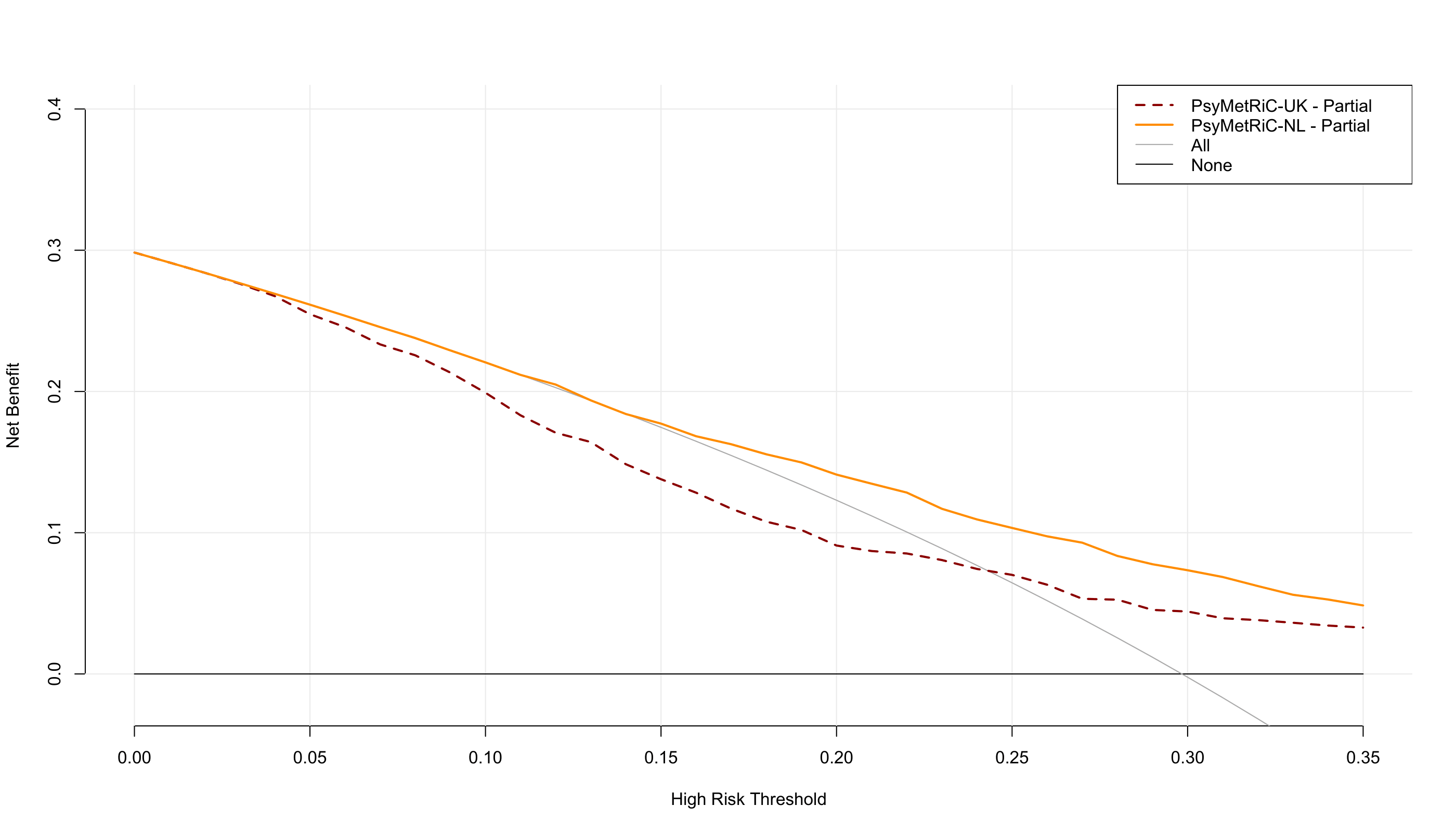** | **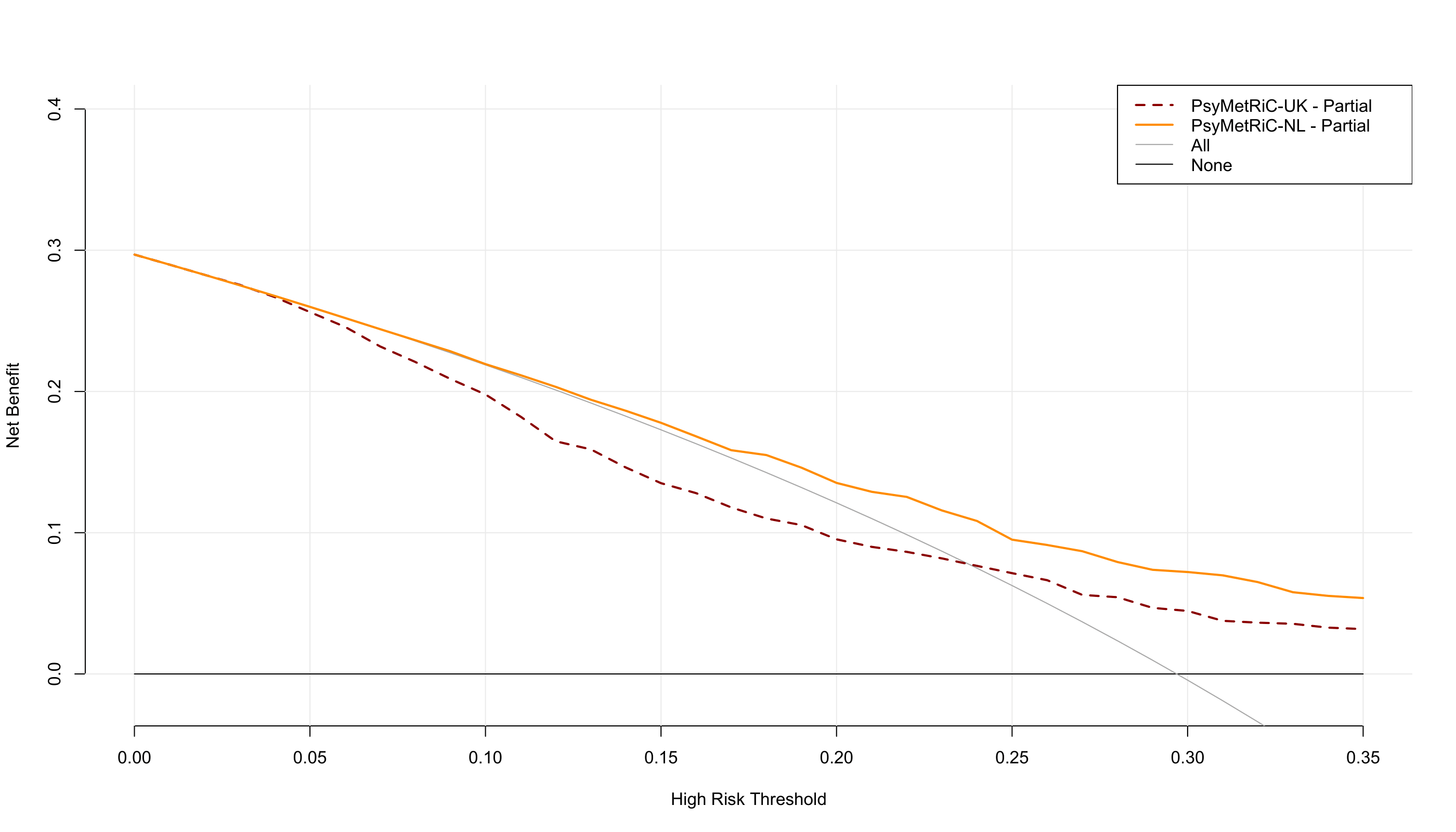** | **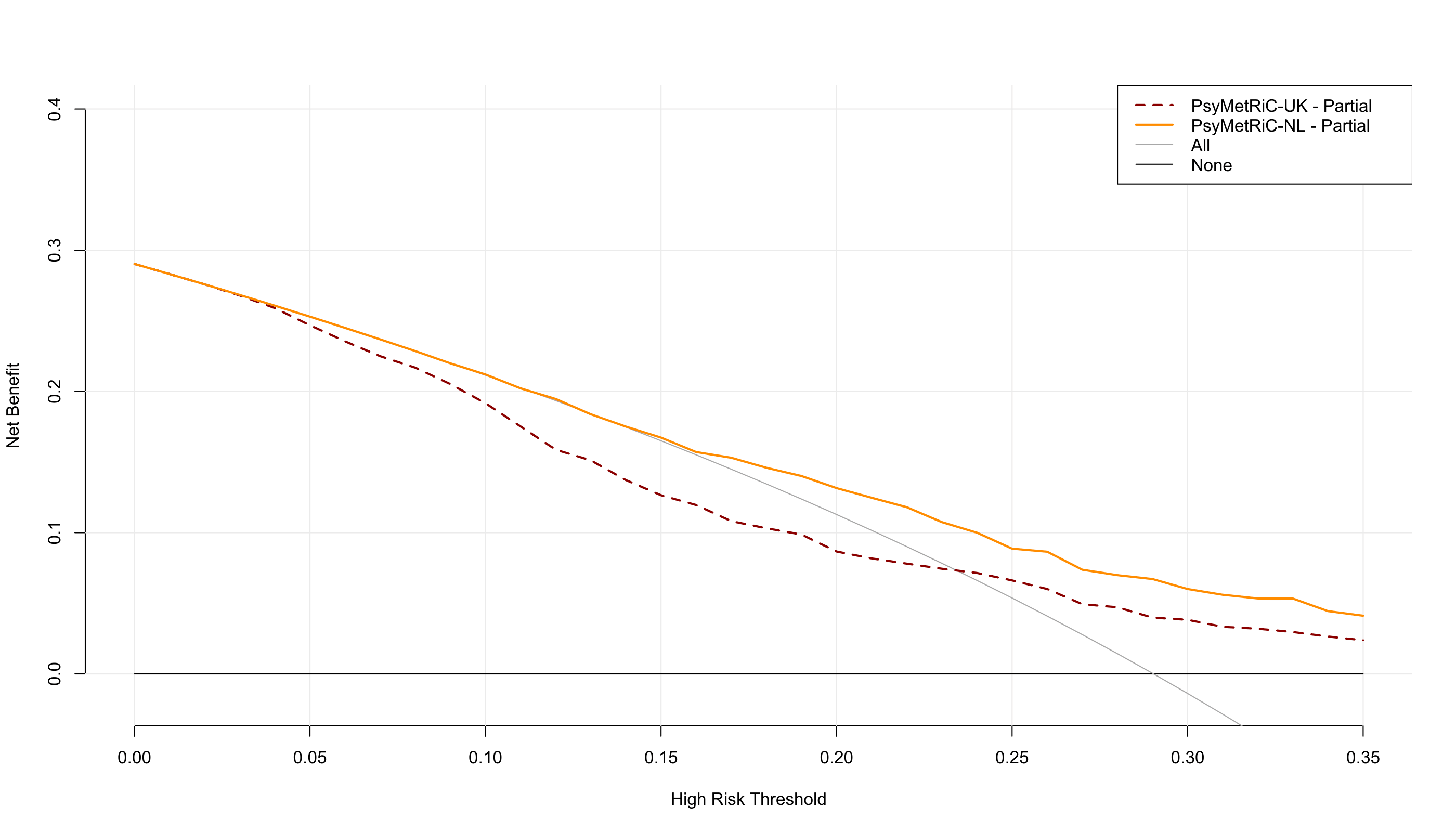** |
