# Supplementary material for: External validation and recalibration of the psychosis metabolic risk calculator (PsyMetRiC) in young adults with chronic psychotic disorders in the Netherlands
Source: Eur Psychiatry. 2026 Mar 9;69(1):e44. doi: 10.1192/j.eurpsy.2026.10179 (PMC13122530; doi:10.1192/j.eurpsy.2026.10179)
Supplement: Quadackers et al. supplementary material [file S0924933826101795sup001.zip › Supplementary Table 1.docx]

**Supplementary Table 1**

| *Category* | *Comparator* | | *Country* | | References for the Netherlands | | |  |  |
| --- | --- | --- | --- | --- | --- | --- | --- | --- | --- |
|  |  |  | United Kingdom* | The Netherlands |  |  |  |  |  |
| General | Area (km^2^) | | 243610 | 41543 | <https://www.worlddata.info/country-comparison.php?country1=GBR&country2=NLD> | | |  |  |
|  | Government Form | | Parliamentary Constitutional Monarchy | Parliamentary Constitutional Monarchy | <https://www.government.nl/topics/constitution> | | |  |  |
|  |  |  |  |  | <https://www.government.nl/government/about-the-government> | | |  |  |
| Demographics | Population, millions | | 68·35 | 17·88 | <https://www.worlddata.info/country-comparison.php?country1=GBR&country2=NLD> | | |  |  |
|  | Inhabitants/km^2^ | | 280·60 | 430·3 | <https://www.worlddata.info/country-comparison.php?country1=GBR&country2=NLD> | | |  |  |
|  | Life Expectancy (Males) (years) | | 79 | 81 | <https://www.worlddata.info/country-comparison.php?country1=GBR&country2=NLD> | | |  |  |
|  | Life Expectancy (Females) (years) | | 83 | 83 | <https://www.worlddata.info/country-comparison.php?country1=GBR&country2=NLD> | | |  |  |
|  | Quality of Life Score^1^ – Political Stability^2^ | | 78 | 84 | <https://www.worlddata.info/country-comparison.php?country1=GBR&country2=NLD> | | |  |  |
|  | Quality of Life Score^1^ – Civil Rights^3^ | | 89 | 98 | <https://www.worlddata.info/country-comparison.php?country1=GBR&country2=NLD> | | |  |  |
|  | Quality of Life Score^1^ – Health^4^ | | 78 | 84 | <https://www.worlddata.info/country-comparison.php?country1=GBR&country2=NLD> | | |  |  |
|  | Quality of Life Score^1^ – Cost of Living^5^ | | 32 | 38 | <https://www.worlddata.info/country-comparison.php?country1=GBR&country2=NLD> | | |  |  |
|  | Prevalence of Smoking (%)^6^ | | 13·0 | 15·0 | <https://www.worlddata.info/country-comparison.php?country1=GBR&country2=NLD> | | |  |  |
|  | Dominant Language (language, %) | | English (97) | Dutch (96) | <https://www.worlddata.info/country-comparison.php?country1=GBR&country2=NLD> | | |  |  |
|  | Dominant Religion (religion, %) | | Nondenominational (52) | Christianity (47) | https://www.worlddata.info/country-comparison.php?country1=GBR&country2=NLD | | |  |  |
|  | Ethnic Fractionalization Score^7^ | | 0·12 (in 2013) | 0·11 (in 2013) | <https://worldpopulationreview.com/country-rankings/most-diverse-countries> | | |  |  |
|  | Linguistic Fractionalization Score^7^ | | 0·05 | 0·51 | https://worldpopulationreview.com/country-rankings/most-diverse-countries | | |  |  |
|  | Religious Fractionalization Score^7^ | | 0·69 (in 2013) | 0·72 (in 2013) | https://worldpopulationreview.com/country-rankings/most-diverse-countries | | |  |  |
|  | Annual Net Migration per 1000 Inhabitants^8^ | | 3·9 (in 2018) | 5·00 (in 2018) | <https://data.un.org/Data.aspx?d=PopDiv&f=variableID%3A85> | | |  |  |
| Economy | Unemployment Rate (%) | | 4·1 | 3·6 | <https://www.worlddata.info/unemployment-rates.php> | | |  |  |
|  | Median income (USD) | | 39830 | 39100 | <https://longreads.cbs.nl/the-netherlands-in-numbers-2023/what-is-working-peoples-income/> | | |  |  |
|  | Corruption Index^9^ | | 78 | 82 | <https://www.transparency.org/en/cpi/2021/index/nld> | | |  |  |
|  | GDP per 1000 Inhabitants (USD, millions) | | 56·24 | 80·7 | <https://w3.unece.org/CountriesInFigures/en/Home/Index?countryCode=528> | | |  |  |
| Healthcare | Hospital Beds per 1000 | | 2·93 (in 2010) | 4·12 (in 2010) | <https://gateway.euro.who.int/en/country-profiles/netherlands/> | | |  |  |
|  | Health Expenditure (%GDP)^10^ | | 9·8 (in 2015) | 10·8 (in 2015) | <https://www.oecd.org/content/dam/oecd/en/publications/reports/2016/11/health-at-a-glance-europe-2016_g1g71832/9789264265592-en.pdf> | | |  |  |
|  | Psychiatric Inpatient Beds per 1000^11^ | | 0·61 (in 2009) | 1·39 (in 2009) | <https://gateway.euro.who.int/en/hfa-explorer/> | | |  |  |
|  | Medical Doctors per 1000 | | 3·17 (in 2025) | 3·91 (in 2025) | <https://worldpopulationreview.com/country-rankings/doctors-per-capita-by-country> | | |  |  |
|  | T2D Hospitalization per 100,000 | | 64·3 (in 2013) | 68·3 (in 2013) | <http://dx.doi.org/10.1787/888933281111> | | |  |  |
|  | CHF Hospitalization per 100,000 | | 99·4 (in 2013) | 199·4 (in 2013) | <http://dx.doi.org/10.1787/888933281105> | | |  |  |
|  | CT Scanners per 1,000,000 | | 9·46 (in 2014) | 13·34 (in 2014) | <https://www.oecd.org/en/data/indicators/computed-tomography-ct-scanners.html?oecdcontrol-00b22b2429-var3=2014> | | |  |  |
| *Reference: <https://www.thelancet.com/cms/10.1016/j.lanepe.2022.100493/attachment/bfa85584-5e94-4c1e-af48-536b8280e953/mmc3.docx> | | | | | | | | | |
| (We selected the most recent year for which data were available for both countries, based on the source cited in the 'References for the Netherlands' column) | | | | | | | | | |
|  | |  | | |  |  |  | |  |
| Information obtained from worlddata.info unless otherwise stated. | | | | |  |  |  | |  |
| See <https://www.worlddata.info/quality-of-life.php> for more information on how scores were created. | | | | | |  |  | |  |
| ^1^scored from 0 (worst) – 100 (best). | | | | |  |  |  | |  |
| ^2^A metric comprising economic inflation, government debt/deficits, gross domestic product, unemployment rate, and ratio of available money supply to currency reserves. | | | | | | | | |  |
| ^3^A metric comprising the regulatory quality of the government, democratic participation by populus, and the corruption index from Transparency.org. | | | | | | |  | |  |
| ^4^A metric comprising average life expectancy, drinking water supply, and the number of medical doctors and hospital beds. | | | | | | |  | |  |
| ^5^A metric comprising cost of living, median income, government taxation, and The World Bank ‘Ease of Doing Business’ index. | | | | | | |  | |  |
| ^6^Data obtained via The World Bank <https://data.worldbank.org>; | | | | |  |  |  | |  |
| ^7^Fractionalization scores are commonly used metrics in economics, and show the probability that two randomly drawn individuals from the population are not from the same group (e.g., ethnic, religious, linguistic). | | | | | | | | | |
| ^8^Data obtained from United Nations <https://population.un.org/>. | | | | |  |  |  | |  |
| ^9^based on scores from Transparency International Corruption Perceptions Index (<https://www.transparency.org/en/cpi/2021/index/>). | | | | | | |  | |  |
| ^10^Taken from OECD Health At a Glance: Europe 2016 Report <https://read.oecd-ilibrary.org/social-issues-migration-health/health-at-a-glance-europe-2016>. | | | | | | |  | |  |
| ^11^Taken from World Health Organization European Health Information Gateway. | | | | |  |  |  | |  |
| GDP=gross domestic product; T2D=type 2 diabetes; CHF=congestive heart failure. | | | | | | | | | |
