# Supplementary figures and images for: External validation and recalibration of the psychosis metabolic risk calculator (PsyMetRiC) in young adults with chronic psychotic disorders in the Netherlands
Source: Eur Psychiatry. 2026 Mar 9;69(1):e44. doi: 10.1192/j.eurpsy.2026.10179 (PMC13122530; doi:10.1192/j.eurpsy.2026.10179)

**Supplementary Figure 1**


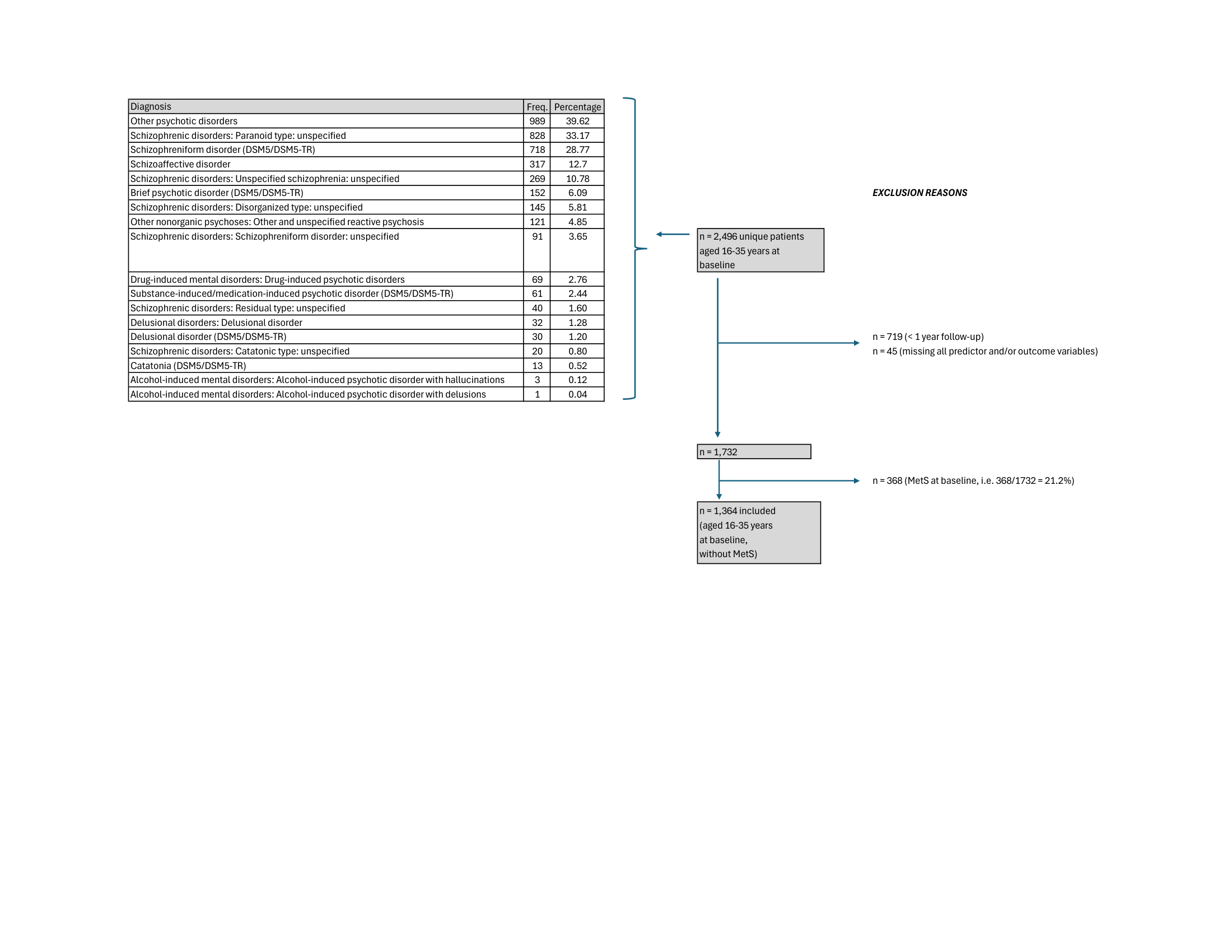


**Flowchart illustrating the inclusion process of n=1,364 study participants.**

Supplement: Quadackers et al. supplementary material [file S0924933826101795sup001.zip › Supplementary Figure 1.docx]
